# The Extract of *Ginkgo biloba* EGb 761 Reactivates a Juvenile Profile in the Skeletal Muscle of Sarcopenic Rats by Transcriptional Reprogramming

**DOI:** 10.1371/journal.pone.0007998

**Published:** 2009-11-24

**Authors:** Caroline Bidon, Joël Lachuer, Jordi Molgó, Anne Wierinckx, Sabine de la Porte, Bernadette Pignol, Yves Christen, Rolando Meloni, Herbert Koenig, Nicole Faucon Biguet, Jacques Mallet

**Affiliations:** 1 C.N.R.S., Biotechnologie & Biotherapie UMR 7225, Hôpital de la Pitié-Salpêtrière, Paris, France; 2 I.N.S.E.R.M. CRICM UMR S_975, Hôpital de la Pitié-Salpêtrière, Paris, France; 3 UPMC Univ Paris 06, Paris, France; 4 I.N.S.E.R.M. Laboratoire Neuro-oncologie et Neuro-inflammation U842, Lyon, France; 5 ProfileXpert, IFR19, Bron France; 6 Université de Lyon, Lyon1, UMR-S842, Lyon, France; 7 C.N.R.S. Institut de Neurobiologie Alfred Fessard, Laboratoire de Neurobiologie Cellulaire et Moléculaire UPR9040, Gif sur Yvette, France; 8 Ipsen Research Laboratories, Les Ulis, France; 9 Ipsen, 65 Quai Georges Gorse, Boulogne Billancourt France; University of Valencia, Spain

## Abstract

**Background:**

Sarcopenia is a major public health problem in industrialized nations, placing an increasing burden on public healthcare systems because the loss of skeletal muscle mass and strength that characterizes this affection increases the dependence and the risk of injury caused by sudden falls in elderly people. Albeit exercise and caloric restriction improve sarcopenia-associated decline of the muscular performances, a more suitable and focused pharmacological treatment is still lacking.

**Methodology/Principal Findings:**

In order to evaluate such a possible treatment, we investigated the effects of EGb 761, a *Ginkgo biloba* extract used in chronic age-dependent neurological disorders, on the function of the *soleus* muscle in aged rats. EGb 761 induced a gain in muscular mass that was associated with an improvement of the muscular performances as assessed by biochemical and electrophysiological tests. DNA microarray analysis shows that these modifications are accompanied by the transcriptional reprogramming of genes related to myogenesis through the TGFβ signaling pathway and to energy production via fatty acids and glucose oxidation. EGb 761 restored a more juvenile gene expression pattern by regenerating the aged muscle and reversing the age-related metabolic shift from lipids to glucose utilization.

**Conclusions/Significance:**

Thus, EGb 761 may represent a novel treatment for sarcopenia both more manageable and less cumbersome than exercise and caloric restriction.

## Introduction

Sarcopenia, a term coined by Rosenberg [1], is the major feature of the age-related decline in neuromuscular performances and is characterized by the loss of skeletal muscle mass as well as strength [2]. These morphological and functional modifications result from intrinsic events, such as changes in the muscle fiber type composition, mitochondrial dysfunction and oxidative damage, and from extrinsic factors including reduced physical activity and excessive and/or unbalanced nutritional intake [3]. Exercise and caloric restriction (CR), known to slow down the impairment of the aged muscle performances [4], are difficult to manage while a more specific pharmacological treatment for sarcopenia that would be more suitable for elderly people is still lacking.

Muscle wasting and weakness associated with sarcopenia may benefit from drugs that target neurodegenerative diseases, since sarcopenia shares several features with different age-related neuromuscular disorders [5]. In this perspective, one of such drugs is EGb 761, a *Ginkgo biloba* extract, which has been shown to be an effective treatment in chronic age-dependent neurological disorders such as, for example, Alzheimer's disease [6], [7]. Therefore, we treated with EGb 761 aged sarcopenic rats in order to evaluate whether this compound, similarly to exercise and CR, improves age-impaired muscular functions. Biochemical and electrophysiological analyses show that EGb 761 has a protective effect on muscular wasting and improves *soleus* muscle isometric contractile force. These effects are associated, as shown by DNA microarray analysis, with the restoration of a more juvenile transcriptional activity of genes relevant for regeneration and energy production processes in the *soleus* of aged rats. This is the first time, to our knowledge, that EGb 761 has been shown to benefit the aged muscle in mammals suggesting that it may represent a novel treatment for sarcopenia.

## Results

### EGb 761 Affects Total Body and Muscle Weights

At the beginning of the experiment, 22 month-old rats were randomly separated in two groups and were weighted at regular intervals. The first group, Aged Control (AC), did not receive any treatment, while the second group, Aged Treated (AT), received EGb 761 in the drinking water. After 5 weeks of this regimen, the mean body weight of the AC group was increased by 2.2% while the rats in the AT group lost 4% (p = 0.02) of their initial body weight, indicating that EGb 761 counteracts the weight gain naturally occurring in aged laboratory rats (Table 1).

**Table 1:** Body and *soleus* muscle weight.

Aged rats	Control (11)	Treated (12)	Young rats (12)	
*Body weight at T0 (22 months)*	605.64±24.66	594.10±13.74	*Body weight at T0 (3 months)*	493.25±7.28
*Body weight at T0 +5 weeks*	619.64±28.35	570.00±15.66	*Body weight at T0 +5 weeks*	502.27±9.98
*Soleus weight*	132.73±8.92	141.33±7.41	*Soleus weight*	152.36±3.53
*Soleus/body weight ratio*	0.22±0.02	0.25±0.02	*Soleus/body weight ratio*	0.31±0.00

Values represent the mean±S.E.M. Weights are expressed in g for total body and in mg for the *soleus* muscle. The ratio represent the muscle weight (mg) divided for the total body weight (in g) for the different rat groups.

In order to ascertain whether this effect of EGb 761 was correlated with differences in muscle anatomic-functional features, we proceeded to further analyses and focused our study on the *soleus* muscle, a slow twitch muscle (type I fiber) with predominant oxidative metabolism. The animals were euthanized and the *soleus* muscles dissected and weighted. The *soleus* mean weight was increased by 6.5% in the AT compared to the AC group of rats. Moreover, the ratio of the soleus weight reported to the total body weight was increased by 13% (Table 1).

This result suggests that EGb 761 affects body and muscle weight by increasing the muscular mass and, thus, reversing toward the juvenile values the age-related decrease of the ratio of muscle v/s body weight. In order to evaluate whether this phenomenon was also associated with variations of a biochemical index of age-related muscle wasting processes, we assessed the effect of the EGb 761 treatment on the circulating creatine kinase.

### EGb 761 Decreases Serum Creatine Kinase

We observed a striking decrease in the serum creatine kinase (CK) levels in AT rats (371±223 U/L), compared to AC rats (737±178 U/L). Interestingly, the CK value obtained in the AT rats was closely similar to the value observed in a group of young (Y) 4 month-old rats (370±53 U/L). Since CK serum levels act as a peripheral marker of muscle damage, these findings further suggest that EGb 761 has a protective effect against the damages that affect the aging muscle. In order to ascertain whether this protective effect was also associated with increased muscular performances, we compared the contractile force of the *soleus* muscle under different conditions in the AC and AT groups of rats.

### EGb 761 Enhances the Isometric Contractile Force

**Figure 1:**
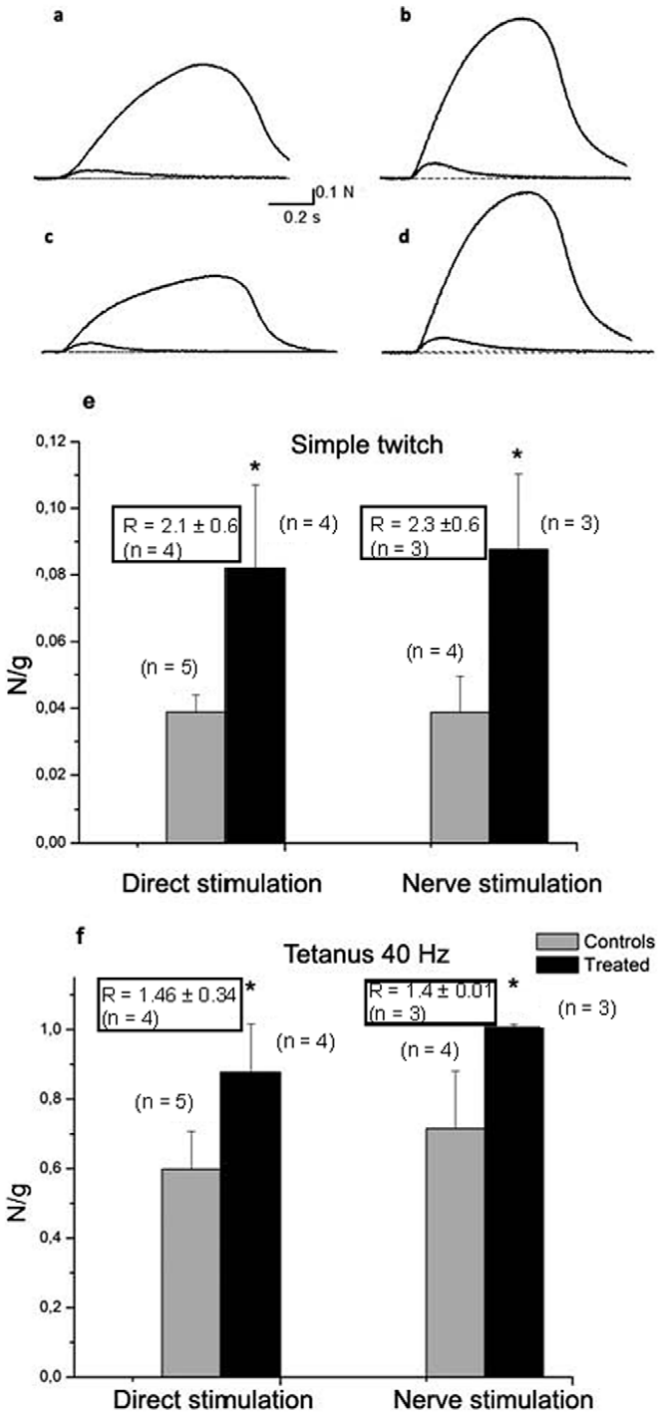
Contractility studies. Representative examples of isometric contractile force in isolated soleus muscle from aged controls (a, c), and EGb 761-treated rats (b, d). In a and b the recordings were obtained by direct muscle stimulation and in c and d by nerve stimulation. In each recording (a to d) are shown evoked twitches (0.2 Hz stimulation) and tetanic contractions (40 Hz, 600 ms stimulation). The force is expressed in Newton (N). Histograms represent mean values±S.E.M. of the force (expressed in N/g) for the single twitch (e) and for tetanic stimulation (f). Mean ratio values between treated and controls are indicated in respective boxes with number of values (n) used for calculations. * Significantly different from controls *p*<0.05.

We measured the maximal force developed by the *soleus* muscles of AC and AT rats, either during a single intermittent isometric twitch or during tetanic contractions, evoked by direct muscle stimulation and through nerve stimulation (Fig. 1, a–d). The isometric twitch and tetanus force developed by individual AT muscles, evoked by direct muscle stimulation, were compared to the mean values for the AC muscles, normalized to the weight of the muscle (Fig. 1, e, f). The mean ratio between AT versus AC was 2.1 for twitch muscle force evoked by direct stimulation (Fig. 1e) and 1.46 for tetanic contractions at 40 Hz (Fig. 1f), indicating an improvement of muscle force. For nerve-evoked contractions, similar values were obtained with a mean ratio of 2.3 for the single twitch (Fig. 1e) and 1.4 for tetanic contraction at 40 Hz (Fig. 1f).

**Figure 2:**
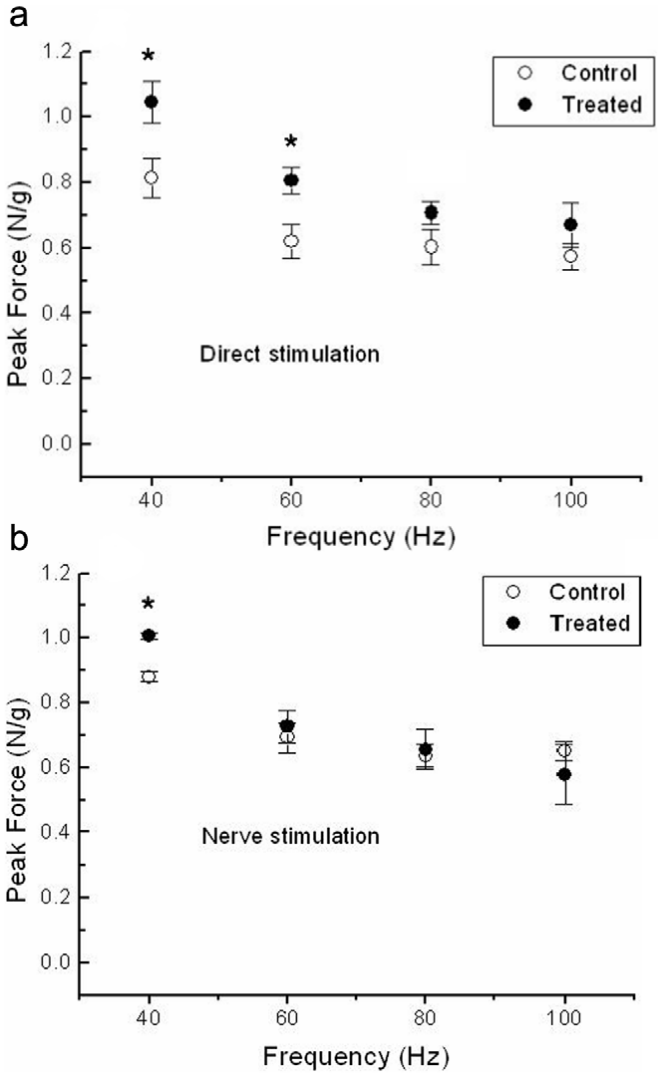
Force-frequency relationship. Peak force during isometric tetanic responses in isolated soleus muscles from aged controls (○) and EGb 761-treated rats (•) obtained by direct muscle stimulation (A) and by nerve stimulation (B) at frequencies indicated (abscissa) during trains of 600 ms duration. An interval of 30 s was used between trains. Each circle represent the mean±S.E.M. (n = 3). * Significant different from controls p<0.05.

Furthermore, analyses of the peak force-frequency relationship during direct tetanic stimulation (40–100 Hz) revealed that AT *soleus* muscles develop significantly higher peak force at 40 and 60 Hz when compared to AC muscles (Fig. 2a). Also, a significant change on nerve-evoked tetanus peak force was detected at 40 Hz in AT muscles when compared to controls (Fig. 2b). Taken together, these results indicate that isometric force developed by *soleus* muscle from EGb 761-treated aged rats is improved with respect to the same-age controls, with an enhancement dependent on the stimulation frequency since no significant improvement was detected at the higher stimulation frequencies studied.

Thereafter, in order to gain insight on the molecular mechanisms underlying these anatomical, biochemical and physiological modifications elicited by EGb 761, we analyzed the global gene expression in the rat *soleus* muscle by DNA microarray analysis. The results already obtained point out to some precise phenomena based on regeneration or energy production amelioration for the improvement of muscular functions elicited by EGb 761. Therefore, the analysis of DNA microarray experiments investigating molecular events associated with aging and with EGb 761 treatment on global gene transcription was focused on genes implicated in these processes on a limited number of rats for each group.

### EGb 761 Treatment Elicits Transcriptional Reprogramming in the *Soleus* Muscle of the Aged Rat

**Figure 3:**
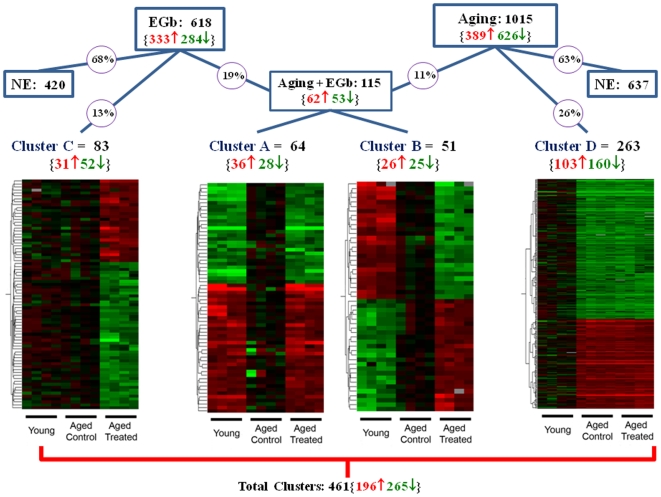
Microarray data analysis and clustering. Comparison of gene expression profiles of *soleus* muscle in young rats (Y), aged control rats (AC) and aged treated rats (AT). Genes are clustered using Pearson's correlation. Down-regulated genes are represented in green, up-regulated genes, in red, number of genes is reported in parentheses.

The DNA microarray analysis identified a total of 1518 genes, out of 9906, modulated either by aging or EGb 761 or both. Aging affected the expression of 1015 genes, 2/3 of them, for a total of 626, were down-regulated, while the remaining 389 were up-regulated, as assessed by comparing the old sarcopenic (AC) versus the young group (Y) of rats (Table S1). EGb 761 affected the expression of 618 genes in nearly equivalent proportions; 333 genes were up-regulated and 284 genes were down-regulated when comparing the treated (AT) versus the AC group of aged rats (Table S2). Among the genes modulated by EGb 761, a total of 115 were also modified by aging (Fig. 3). The DNA microarray data has been loaded into the ArrayExpress database (ArrayExpress accession: E-MEXP-1937, release date: 2009-06-30).

The microarray data was further investigated using hierarchical clustering analysis based on the overall similarity of the variations in the genes expression patterns. Permutation analysis was conducted on the three different groups of rats altogether. About 2/3 of the genes modulated by EGb 761 or Aging showed more than 30% of expression variability in each of the 3 groups. These genes were classified as non exploitable (NE) and were not used for the clustering analysis (see Methods) (Fig. 3). Using this very stringent approach, four different clusters (A to D) were identified by Pearson correlation analysis, corresponding to the different types of gene expression behavior in response to aging and/or treatment (Fig. 3). The first cluster assembles genes that are differentially expressed during aging and, then, reverted toward the young expression level by EGb 761 (Cluster A, Table S3). The second cluster contains genes that display an additive effect of aging and EGb 761 on their expression, that is genes down-regulated or up-regulated during aging and whose expression was further decreased or increased, respectively, by EGb 761 (Cluster B, Table S4). The two remaining clusters assemble genes whose expression is modified by the treatment but not by aging (cluster C, Table S5), and genes modulated by aging but not by the treatment (cluster D, Table S6). Since we were interested in the effect of EGb 761 on aging, we focused our analysis on the cluster A and B, while the clusters C and D were not further investigated.

Thus, according to the results obtained by clustering analysis, EGb 761 modified the expression of 30% (115 out of 378) of the genes affected by aging. This result stresses the impact of the *Ginkgo biloba* extract on aging since these genes represent almost 60% (115 out of 198) of the genes regulated by EGb 761.

### Validation of the DNA Microarray Results by qPCR on Selected Genes

In order to validate the DNA microarray results before further analysis, we analyzed the m-RNA expression levels of 13 selected genes having different levels of expression by real-time quantitative RT-PCR (qPCR) in the *soleus* muscle, including those used for the microarray.

**Figure 4:**
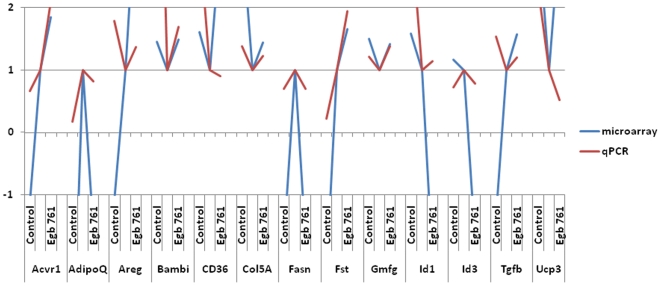
Graphic representation of gene expression variations. Comparison between variations of gene expression in the different groups of rats according either to DNA microarray analysis (blue lines) or to qPCR (red lines). Values of the AC (aged control) group are standardized to 1 for each gene.

The comparison of the pattern of expression in the three different groups of rats between the DNA microarray and the qPCR analysis established that all the genes displaying variations in their expression pattern in the transcriptomic analysis were also showing concomitant variations by qPCR analysis (Fig. 4). Moreover, among these genes *Acvr1*, *AdipoQ*, *Bambi*, *Col5A*, *Fasn*, *Fst* and *Gmfg*, had a similar pattern of variation by DNA microarray and qPCR analysis both when comparing the young with the aged group of rats as well as when comparing the aged with the aged-treated group of rats. Among the remaining genes, *Cd36*, *Id1* and *Ucp3*, displayed a similar pattern when comparing the young with the aged group of rats and *Areg*, *Id3* and *Tgf-β*, when comparing the aged with the aged-treated group of rats (Fig. 4). Therefore, a majority of 20 out of 26 (77%) of the comparison made with the 3 groups of rats for these 13 genes were confirmed by qPCR, validating for further analysis the DNA microarray data.

### The EGb 761 Affects Genes Implicated in Regeneration and Energy Production Functions

**Figure 5:**
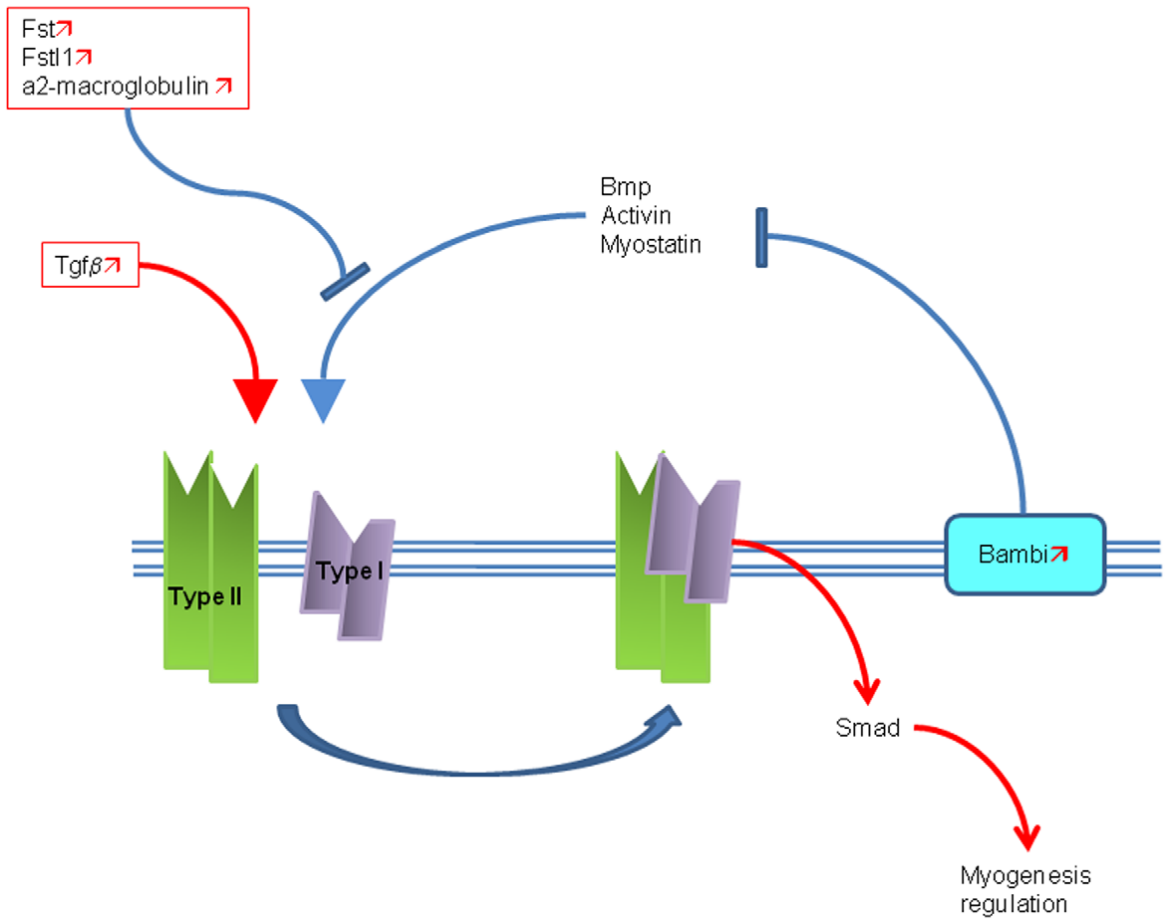
Schematic representation of the interactions between the genes regulated by EGb 761 associated to myogenesis/regeneration in the *soleus* muscle of aged rats. Regulated genes are included in frames. 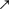 indicates up-regulation of gene expression by EGb 761. 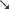 indicates down-regulation of gene expression by EGb 761. → = activation. --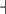 = inhibition. Activins and BMPs may be diverted into alternative pathways through interaction with soluble and membrane-bound binding proteins Activin, myostatin and BMPs signal via type II activin receptor. The access of these TGF-β2 superfamily members to their type II receptors is blocked by extracellular binding proteins (follistatine, Fstl1 and α2-macroglubulin) and membrane-bound pseudoreceptors (BAMBI for activin signaling).

We classified all the genes belonging to clusters A and B according to their biological functions, using the Ingenuity pathway analysis software and the gene ontology database. The most relevant of these functions, according to the number of genes modified both by aging and EGb 761 treatment, are related to cell cycle, cell death, growth and proliferation, as well as metabolism (Table 2). Among the most relevant genes, there are cell division control protein 42 (*Cdc42*) and transforming growth factor β2 (*Tgfb2*), which are ubiquitous factors that intervene also in the development and regeneration of the skeletal, nervous and connective tissues. Moreover, the EGb 761 treatment was also associated with the up-regulation by 50 to 70% of genes specifically implicated in skeletal muscle development such as Follistatin (*Fst*), Follistatin related protein (*Fstl1*), BMP and activin membrane-bound inhibitor (*Bambi*), α2-macroglobulin (*A2m*), Activin receptor type I (*Acvr1*), embryonic Myosin heavy chain (*Myh3*) and Ryanodine receptor 3 (*Ryr3*). Since several of these genes participates also in the TGFβ signaling pathway, which plays a relevant role in the muscle satellite cell proliferation and differentiation [8], these results suggest that the EGb 761 modifies gene expression in a manner consistent with the remodeling of the sarcopenic muscle via the modulation of the myogenesis (Fig. 5).

**Table 2:** List of the genes modulated both during aging and after EGb-treatment classified according to the Gene Ontology database.

Function	Number of Genes Regulated (up/down)	Symbol
**Cell Cycle**	**23**/11	**A2M, ACPP, AIF1, AREG, ARID1A, CDC42, CDK4, COL1A1, CREG1, FRK, LGALS3, MAPK14, MPG, P1K3R1, PAWR, PROS1, PVR, RAE1, ROCK2, SDC2, SST, TGFB2, TNC, TXNIP, XPC/**AKAP12, FASN, ID1, ID3, IGFBP5, MSH2, NOTCH1, PRKCH, PLAC8, THBS1, TOB2
**Cell Death**	**20**/16	**A2M, BIRC2, CDC42, CDH2, DSP, GCLC, HSPD1, IL18, IL6ST, MLLT3, MPG, P4HB, P4WR, RTN4, RRAS2, SDC2, SST, TGFB2, TXNIP, UCP1/**ADIPO, ANTRK2, CCL11, CCL27, COL18A1, FASN, FKBP5, ID1,IGFBP5, JAG2, PDE4, PIK3R1, PTPN13, RTN4RL1, THBS1, VTN
**Cellular Growth and Proliferation**	**43**/42	**ACPP, AIF1, AREG, ARL6IP5, BAMBI, C1QTNF3, CDC42, CDK4, CDK7, COL1A1, CREG1, CX3CL1, CXCR7, CYP1A1, DSP, EZR, FST, HSPD1, IL18, IL2RB, IL6ST, LCP1, LGALS3, LITAF, LOC643751, MAPK14, MLLT3, MT1F, NOP5/NOP58, NR1H3, PAWR, POSTN, RASA1, RHOBTB2, RTN4RRAD, SDC2, SEPT9, SST, TGFB2, TNC, TXNIP, UCP3, XPC/**ADIPOQ, AGER, AKAP12, AQP4, BCAR3, C6ORF108, CCL11, CCL27, CMA1, COL18A1, DLC1, EGR2, ETV6, FAM107A, FASN, GLA, GPX3, HK1, ID1, ID3, IFIT3, IGFBP5, IHPK2, ITGB4, LCP1, LDHA, LTBP4, NOTCH1, NTRK2, PALM, PENK, PER1, PLAC8, PRKCH, PTPN13, PTPN3, RASGRP2, RTN4RL1, SYMPK, THBS1, TIMP4, VTN
**Skeletal and Muscular System**		
Muscle development	**8**/9	**BAMBI, COL5A3, FST, IFRD1, IL6ST, MYH10, MYH3, ROCK2/**DLC1, IGFBP5, ITGB4, NOTCH1, PLAC8, RASGRP2, TAGLN, THBS1, TNNT2
Muscle contraction	**4**/5	**CASQ2, MYBPH, PDEAB, RYR3/**ADRB2, CNN1, MYBPC2, TNN12, TNNT2
Muscle Disorders	**14**/11	**AREG, C2, CD36, FCGR3A, HLA-DMA, IL18, IL2RB, IL6ST, LCP1, MAPK14, PDE4B, PRMT3, RPL32, TAP1/**ADRB2, ALAS2, CCL11, CXCL9, EGR2, FKBP5, G0S2, PDE4A, RAMP2, WNT5B, ZFP36
**Nervous System Development and Function**	**24**/23	**A2M, ARNTL, ATXN10, CDC42, CDH2, CX3CL1, EIF2B5, FRK, FST, HMGB2, IFRD1, IL6ST, LOC643751, MAPK14, MYH10, NR1H3, PAFAH1B3, RTN4, RYR3, SST, SV2B, TGFB2, TIMM8A, TNC/**ADRB2, AGER, ALDH1A7, AQP4, CNTN2, CXCL9, EGR2, GPM6A, HEYL, HTR1A, ID1, ID3, ITGB4, JAG2, NELF, NOTCH1, NTRK2, PDE4A, PER1, RAB3A, RTN4RL1, THBS1, VTN
**Connective Tissue Development and Function**	**10**/6	**CDC42, CDH2, CDK4, IFRD1, MAPK14, PVR, ROCK2, TGFB2, TNC, UCP1/**ADIPOQ, AKAP12, GCGR, IGFBP5, NPYIR, TOB2
**Lipid Metabolism**	**11**/10	**A2M, ABCA1, APOD, CD36, CDC42, CYP1A1, GPR175, MAPK14, NR1H3, PDK4, UCP3/**ACSS2, ADIPOQ, BDH2, CCL11, DGAT2, FASN, GLA, LSR, PLCD4, SMPD3
**Carbohydrate Metabolism**	**14**/18	**CD36, GCKR, GCLC, GNG2, H6PD, IL6ST, MAPK14, PDK4, RAB5A, SLC2A1, SST, UCP1, UCP3, UGP2/**ADIPOQ, ADRB2, ALG2, DCXR, DGAT2, FASN, FN3K, GCGR, GLA, HK1, IHPK2, NPY1R, PFKFB1, PFKL, PITPNM1, PLCD4, SYNJ2, THBS1

The up-regulated genes are presented in bold characters and the down-regulated genes are in plain characters.

The other most relevant effect of EGb 761 is related to the transcriptional reprogramming of genes implicated in metabolic processes in mitochondria. Namely, EGb 761 induced the down-regulation of the transcripts for the Fatty acid synthase (*Fasn*), responsible for fatty acid synthesis, the Acetyl-Coenzyme A acyltransferase2 (*Acaa2*), involved in cholesterol and lipoprotein synthesis and fatty acid elongation, the Hexokinase 1 (*Hk1*) and the Phosphofructokinase 1 (*Pfk1*), both implicated in glucose metabolism. The treatment induced as well the up-regulation of the transcripts for the Pyruvate dehydrogenase kinase 4 (*Pdk4*) and the Fatty acid translocase (*Cd36*) involved in lipid metabolism and transport, respectively, and the Uncoupling proteins 1 and 3 (*Ucp1* and *Ucp3*), relevant for energy production (Table 2).

**Figure 6:**
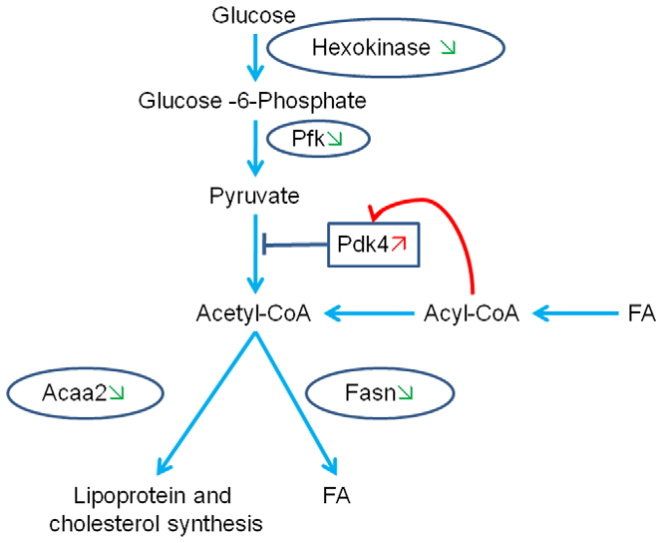
Schematic representation of the interactions between the genes regulated by EGb 761 associated with regulation of energy metabolism in mitochondria in the *soleus* muscle of aged rats. Regulated genes are included in frames. 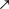 indicates up-regulation of gene expression by EGb 761. 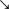 indicates down-regulation of gene expression by EGb 761. → = activation. ----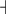 = inhibition. FA (Free Fatty Acids) are taken up by the fatty acid transporter protein Cd36 and activated to fatty acyl-coenzyme A (FA-CoA). FA-CoA oxidation would increase the ratios of acetyl-CoA/CoA and of NADH/NAD+, which inhibit the pyruvate dehydogenase (PDH) complex by activating the pyruvate dehydrogenase kinase 4 (Pdk4). Increase FA oxidation products as citrate should further inhibit phosphofructokinase (Pfk) and hexokinase. These changes would slow down oxidation of glucose and pyruvate and glycogen stock is maintained. In the same time, fatty acid synthase (Fasn) and mitochondrial 3-oxoacyl-Coenzyme A thiolase (Acaa2) are inhibited resulting in the lipid synthesis inhibition and a preferential use of these lipids for ATP synthesis.

These findings are consistent with a previous observation that the EGb 761 modifies the mitochondrial functions in several tissues [9] and suggest that EGb 761 coordinates the expression of genes that enhance mitochondrial energy production by increasing lipid beta oxidation and inhibiting glucose oxidation (Fig. 6).

## Discussion

Sarcopenia is a major public health problem in industrialized nations, placing an increasing burden on public healthcare systems. The progressive loss of muscle mass associated with advancing age that is characterized by the slowing of movement and a gradual decline in muscle strength, increase dependence and the risk of injury from sudden falls in elderly people. Although caloric restriction and physical exercise have been shown to be effective treatments for sarcopenia, their effect is limited to slowing down the progressive loss of muscle mass and functions and their implementation is problematic in elderly people. Therefore, drug treatments appear to be more appropriate for sarcopenia, albeit, until now, no pharmacological therapy has been reported apart from the suggestion that leucine-rich dietary components may help the elderly to preserve muscle mass [10]. However, since the ageing process manifests itself with similar features in different tissues, it has been suggested that drugs for age-related neurodegenerative diseases may also be beneficial for sarcopenia [5]. In this context, EGb 761(IPSEN), an extract of *Ginkgo biloba*, has been shown to extend life span of *caenorhabditis elegans* [11] and to enhance learning and longevity in rats [12]. Also, *in vivo* and *in vitro* experiments have demonstrated the efficacy of EGb 761 in protecting against age-related processes such as increase of oxidative stress, brain mitochondrial dysfunction [9] and chronic age-dependent neurological disorders [6], [7]. Here, we demonstrate that EGb 761 improves muscle performances, as assessed by electrophysiological studies, and, quite remarkably, restores a “juvenile” profile in the *soleus* muscle of old rats by modulating the transcriptional expression of genes implicated in development/regeneration and energy production pathways.

We have focused our study and the DNA microarray analysis on the *soleus* since this muscle is, in the rat and humans, almost homogeneously composed by slow twitch type I fibers. Thus, the age related changes are easier to analyze as they do not result from fiber type modifications as, for example, in the *gastrocnemius* and/or other mixed fiber type muscles.

The transcriptomic analysis revealed that EGb 761 up-regulates *TGFβ* and several other genes such as *Fst*, *Fstl1*, *Bambi* and *Acvr1* that participate to the modulation of the TGFβ signaling pathway during myogenesis. *Tgfb2* contributes, with other factors of the TGF super-family such as BMP, Activin and Myostatin, to myogenesis [13] (Fig. 5) and may participate to the regeneration process that is impaired by aging, resulting in the incomplete structural and functional recovery after muscle injuries in elderly people [14]. It is noteworthy that TGF-β2 plays a contextual role in myogenesis, since it inhibits proliferation and differentiation but, at the same time, promotes the fusion of the satellite cells depending on the presence or absence of other factors [15].

*Fst*, one of the genes up-regulated by EGb 761 and associated to the TGFβ signaling pathway, acts as a positive regulator of the muscle differentiation [16], since, by binding to myostatin, it prevents the myostatin-induced inhibition of proliferation and differentiation [17]. *Fst* expression increases with age as a protective mechanism in response to the parallel elevation of myostatin, albeit this compensatory effect is ineffective in preventing sarcopenia [18]. Accordingly, our data show an important increase (3 fold) in the expression of *Fst* in the aged rats. However, after EGb 761 the over-expression of this transcript is further boosted by 2 fold (see progression cluster). Moreover, according to our results, others transcripts such as *Bambi*, *Fstl1* and *A2m*, all inhibitors of BMPs and Activin [19], are also up-regulated by EGb 761 but not by aging, suggesting that the concomitant action of all these factors on the TGFβ pathway mediates the regenerative effects of EGb 761.

The transcriptomic analysis highlights another major effect of EGb 761 upon transcripts, such as *Fasn*, *Acaa2*, *Cd36*, *Hk1* and *Pdk4*, coding for key mitochondrial enzymes associated to lipid and glucose metabolism and involved in Krebs cycle, β2-oxidation, fatty acid transport and oxidative stress (Table 2). During aging the mitochondria shift from lipids to glucose as substrate for energy production, which results in increased intramuscular fat accumulation [20], [21]. EGb 761 decreased the expression of *Fasn*, involved in FA synthesis, and *Acaa2*, implicated in cholesterol and lipoprotein synthesis and FA elongation, and increased the expression of *Cd36*, a FA transporter present in the sarcolemma, which promotes FA entry in the muscle cell [22]. Since *Fasn* increases blood triglycerides and, with *Acaa2*, favors the accumulation of intramuscular adipose tissue, the down-regulation of these genes could instead facilitate mitochondrial lipid oxidation (Fig. 6).

The striking four-fold increase of *Ucp3* transcripts observed by the DNA microarray data analysis further stresses the effect of EGb 761 in optimizing FA utilization. UCP3 is an uncoupling protein, preferentially expressed in skeletal muscle, whose over-expression increases the activity of several key mitochondrial enzymes associated with FA oxidation, and decreases intramuscular triglyceride content [23]. Also, UCP3 lowers mitochondrial membrane potential, notably through fatty acid anion export from the matrix, and, consequently, decreases reactive oxygen species (ROS) and prevents mitochondrial damage [23]. Interestingly, the increase of UCPs activity correlates with extended lifespan in long-living avian species [24], [25] and in mice [26].

As a corollary to the facilitation of FA oxidation, EGb 761 also inhibited glucose oxidation in mitochondria by down-regulating *Hk1*, associated with glycolysis, and by up-regulating the glucokinase regulatory protein, which inhibits HK1, and *Pdk4*, which inhibits the pyruvate dehydrogenase complex (Fig. 6). Taken together these modifications contribute to the reprogramming of the “glucose-fatty acid cycle”, in which free FA compete with glucose for mitochondrial oxidation [27], toward an increased utilization of FA, resulting in the increase of energy production and the reduction of mitochondrial ROS generation and of intramuscular fat storage (Fig. 6). Other studies on gene expression that used caloric restriction (CR) or exercise training for preventing sarcopenia have reported that CR either stimulated glycolysis, by the up-regulation of enzymes such glucose-6-phosphate isomerase, and FA synthesis, by over-expressing *Fasn* [28]–[30], or, in agreement with our findings, induced a transcriptional switch toward an increase of FA metabolism and a reduction of lipid biosynthesis in the muscle [31]. Exercise training as well, similarly to EGb 761, improves mitochondrial FA uptake and oxidation, by reducing the lipid stores in the muscle [32], [33]. Thus, EGb 761 produces effects comparable to CR or exercise training and represents a more manageable option for the prevention and treatment of sarcopenia.

Besides these major effects, EGb 761 elicited also the over-expression of transcripts that are usually present during regeneration processes such as *Myh3* [34] and *Ryr3*. The Ryr3, a minor form of ryanodine receptor, regulates the resting free Ca^2+^ concentration and, thus, may favor a sustained contraction with increased force in the skeletal muscle [35]. In addition, the up-regulation of the transcripts for *Myh10* and *Mybph* also contribute to the increase of the muscle strength.

The two-fold increase of Tenascin C (*Tnc*) gives another example of how EGb 761 may modulate transcripts that are relevant for muscle strength. *Tnc*, in the adult muscles, is an extracellular matrix (ECM) molecule that localizes in the tendon along with other components of the ECM such as the collagen I and III, playing an important role in the force transmission and tissue structure maintenance. The collagen constitutes 60–85% of the tendon dry weight and is predominantly type I (∼60%) arranged in tensile-resistant fibers, types III (≤10%), IV (∼2%) [36], [37], V, and VI [38]. In particular, the type I collagen, carrying fibrous tensile strength, allows the transmission of force generated by skeletal muscle into movement [39]. Interestingly, the collagen I, V, VI expression is increased between 50% and 100% by EGb 761. Therefore, these components of the ECM may reorganize the structure of the tendon and participate to the improvement of muscle strength and body stability, whose impairment represents the main cause of falls in elderly people.

Finally, mitochondrial function improvement represents the main factor for the enhancement of muscular strength after EGb 761 treatment. Mitochondria play a central role in the production of cellular energy in the form of ATP, but are also key regulators of apoptosis, which contributes to the aging processes underlying sarcopenia. The age-related reduction in ATP production is responsible of the decrease in contractile force observed during aging. EGb 761 contributes to a better ATP production and availability by improving FA transport and utilization.

In conclusion, EGb 761 may represent a novel therapeutic intervention, endowed with a rejuvenation effect mediated by transcriptional reprogramming of a majority of genes implicated in aging, and with several advantages over CR and exercise to prevent sarcopenia in elderly people. Among these advantages there is a more easily implementation and therefore a better compliance with the treatment. Moreover, the effects of EGb 761 are mediated both by the restoration of the expression of genes that are pathologically down-regulated by aging and the boosting of the expression of genes that are physiologically up-regulated to counteract the effects of the aging process. This is another advantage over exercise and CR whose action is limited to slowing down the muscular ageing progression. It is also noteworthy that this effect may be unique to *Ginkgo biloba* extract. Indeed, *Ginkgo biloba* is one of the oldest life forms and contains many molecules (like ginkgolides) that do not exist in other organisms and are extremely difficult to synthesize. Thus, the natural extract of such a living fossil, shaped by natural selection during several millions of years, may contain several unique molecules that are less disruptive than synthetic products and better suited for fitting with biological systems [40].

## Methods

### Animals

All animal procedures were performed under authorizations of the “Direction des Services Vétérinaires de Paris” of the “Prefecture de Police de Paris” and strictly followed the guidelines for the ethical treatment of the animals set forth by French law (decree N° 87-848 dated 11/19/1987 and modified by the decree N° 2001-464 dated 5/29/2001). The study was conducted with male Wistar rats on a group of young, 4-months old, rats (Young group = Y), and two groups of aged, 23-month old, rats. The aged rats either received (Aged Treated group = AT) or did not receive (Aged Control group = AC) EGb 761 treatment.

### EGb 761 Treatment of Rats

The *Ginkgo Biloba* extract (EGb 761), provided by IPSEN laboratories (France), was administered in the water at a dose of 75 mg/Kg body weight for two months. During the 60 days of the experiment, the animals were weighed each week and the volume of drinking solution was measured. The AC and AT groups were sacrificed at 23 months of age, young rats were sacrificed at 4 months.

### Determination of Serum Creatine Kinase

Blood samples were taken from the hearts of anaesthetized rats, immediately before sacrifice. Activities of serum CK were determined using a Biomerieux kit (enzyline CK NAC optimized 10).

### Measurement of *Soleus* Contractility

Rats were anesthetized with Isoflurane (AErrane®, Baxter S.A., Lessines, Belgium) inhalation before being sacrificed by section of the carotid arteries.

*Soleus* muscles with their attached nerves were carefully removed, mounted in a silicone-lined bath filled with Krebs-Ringer solution continuously perfused with 95% O_2_ and 5% CO_2_ throughout the experiment. The Krebs-Ringer solution was maintained at 22±0.5°C instead of 37°C in order to optimize the experimental conditions, since at a temperature of 37°C the risk of nerve conduction failure is greatly increased. One of *soleus* tendons was securely anchored onto the silicone-coated bath, the other tendon was attached to an FT03 isometric force transducer (Grass Instruments, West Warwick, USA).

Muscle twitches and tetanic contractions were evoked either by stimulating the motor nerve *via* a suction microelectrode, with supramaximal current pulses of 0.15 ms duration, at frequencies indicated in the text, or by direct electrical stimulation. The resting tension was adjusted for each preparation investigated with a mobile micrometer stage (to allow incremental adjustments of muscle length) in order to obtain maximal contractile responses. Signals from the isometric transducer were amplified, collected, and digitized with the aid of a computer equipped with a DT2821 analogue to digital interface board (Data Translation, Marlboro, USA), as previously reported [41]. Data acquisition and analysis were performed with a program kindly provided by Dr. John Dempster (University of Strathclyde, Scotland). All data are expressed as the mean±SEM. The statistical significance of differences between controls and test values was assessed with Student's t-Test (two-tailed). Differences were considered significant if */P/*<0.05.

### Tissue Processing

For microarrays and qPCR experiments the *soleus* muscles from each animal were excised, snap-frozen in liquid nitrogen and stored at −80°C.

### RNA Extraction

Total RNA was isolated from an aliquot of muscle thawed on ice; and extraction was performed by a silica gel-based purification method (RNeasy Mini Kit, Qiagen). For qPCR, total RNA was subjected to DNAse treatment (Qiagen) 30 min at room temperature. Total RNA yield was measured by the OD_260_, and the quality was evaluated on nanochips with the Agilent 2100 Bioanalyzer (Agilent Technologies, Palo Alto, CA, USA).

### Microarrays and analysis

Gene expression levels were measured by using rat oligonucleotide arrays as described previously [42]. The microarrays contain 9906 different genes (CodeLink system, Uniset rat I, GE Healthcare Europe GmbH, Freiburg, Germany). Each experimental group, Aged Treated (AT), Aged Control (AC) and Young (Y) rats, was formed by 2 rats (biological replicates) and the total RNA isolated from the *soleus* muscle was hybridized in duplicate (technical replicates). Thus 4 microarrays were utilized for each group of rats.

After hybridization and washing, the slides were scanned using a Genepix 4000B scanner (Axon, Union City, USA) and Genepix software. The scanned image files were analyzed using CodeLink expression software, version 4.0 (GE Healthcare).

CodeLink software was used to normalize the raw hybridization signal. The threshold of detection was calculated using the normalized signal intensity of the 100 negative controls, spots with lower signal intensities were termed “absent”.

ESTs were analyzed by BLAST research on the NCBI nucleotides databases. The clusters were generated using Pearson correlation. Statistical comparison and filtering were performed using Genespring software 7.0 (Agilent).

A first selection of genes was performed by pair-wise comparisons between AT and AC rats (Egb761 effect) or Y and AT rats (Aging effect). Each sample from one group was compared with each sample from the other group. This permutation analysis comparing 3 different group allows for strengthening the relevance of the findings when a given differences is found across different groups. To identify significant changes, the following criteria were used: 1) p-value less than 0.05; 2) a fold change of 1.3 was used to define a set of significantly up- and down regulated genes; 3) the over 1.3 fold change should be reproducible across all comparisons. Therefore a given mRNA transcript was considered as differentially expressed in the comparison of any two samples if the difference gave a *P* value of ≤0.05 in the Welch ANOVA parametric test, using a multiple test correction (Benjamini and Hochberg False Discovery Rate). Moreover, to avoid false positive genes, in particular for genes with small variation between groups, we removed from the two lists above genes with variability ≥30% inside each of the three groups (Y, A and AT), those genes were classified as non exploitable (NE). Thus, a gene was considered for the clustering analysis only if it met the above criteria in all pairwise comparisons and if the detected signal was above the background for at least one of the compared groups, thereby carrying a statistically significant absolute call ‘present’ or ‘marginal’ in all samples.

### qPCR and Analysis

The qPCRs were performed as described previously [42] with some minor modifications. The primers were purchased from Eurogentec (Seraing, Belgium). All primers had Tms between 59 and 61°C and all products were 100 to 150 bp long. GAPDH and cyclophilin are used as internal standards to control amplification variations due to differences in the starting mRNA concentration.

## Supporting Information

Table S1 (3.03 MB PDF)

Table S2 (2.19 MB PDF)

Table S3 (1.10 MB PDF)

Table S4 (0.85 MB PDF)

Table S5 (1.12 MB PDF)

Table S6 (1.39 MB PDF)
