# Supplementary material for: The Extract of Ginkgo biloba EGb 761 Reactivates a Juvenile Profile in the Skeletal Muscle of Sarcopenic Rats by Transcriptional Reprogramming
Source: PLoS One. 2009 Nov 24;4(11):e7998. doi: 10.1371/journal.pone.0007998 (PMC2778626; doi:10.1371/journal.pone.0007998)
Supplement: Table S1 — (3.03 MB PDF) [file pone.0007998.s001.pdf]

Table S1: Aging

Accession	Young	Aged Control	Aged Treated	Symbol	Description
**AW434308**	1.00	36.90	-1.86	C12orf44	chromosome 12 open reading frame 44
**AI412189**	1.00	20.79	6.15	Igh-2	immunoglobulin heavy chain 2 (serum IgA)
**NM_017081.1**	1.00	14.95	23.06	Hsd11b2	hydroxysteroid (11-beta) dehydrogenase 2
**BF416240**	1.00	9.71	8.87	Loc283677	hypothetical LOC283677
**NM_031150.1**	1.00	8.33	2.67	Zp2	zona pellucida glycoprotein 2 (sperm receptor)
**AW251681**	1.00	7.69	7.53	Null	UI-R-BJ0-ADP-F-06-0-UI.S1 UI-R-BJ0
**AW915407**	1.00	6.45	7.68	Null	EST346711
**NM_022507.1**	1.00	6.33	3.21	Prkcz	protein kinase C, zeta
**NM_053719.1**	1.00	6.10	16.89	Emb	embigin homolog (mouse)
**NM_012676.1**	1.00	5.49	3.51	Tnnt2	troponin T type 2 (cardiac)
**BE100748**	1.00	5.29	3.16	C13orf30	chromosome 13 open reading frame 30
**AW919277**	1.00	5.03	3.83	Capds2	Ca2+-dependent activator protein for secretion 2
**BF401313**	1.00	4.98	9.01	Ppp1r14c	protein phosphatase 1, regulatory (inhibitor) subunit 14C
**NM_017325.1**	1.00	4.72	7.62	Runx1	runt-related transcription factor 1 (acute myeloid leukemia 1; aml1 oncogene)
**AW918418**	1.00	4.57	5.32	Dusp13	dual specificity phosphatase 13
**NM_019145.1**	1.00	4.57	4.85	Chrng	cholinergic receptor, nicotinic, gamma
**NM_031688.1**	1.00	4.46	9.04	Sncg	synuclein, gamma (breast cancer-specific protein 1)
**NM_031509.1**	1.00	4.31	3.91	Gsta1	glutathione S-transferase A1
**NM_017139.1**	1.00	4.31	2.73	Penk	proenkephalin
**AW915595**	1.00	3.98	2.53	Adipoq	adiponectin, C1Q and collagen domain containing
**AW918022**	1.00	3.98	2.48	Mamdc2	MAM domain containing 2
**BF419319**	1.00	3.95	5.61	Oasl1	2'-5'-oligoadenylate synthetase-like
**AF322216**	1.00	3.89	2.80	Igsf1	immunoglobulin superfamily, member 1
**BE113616**	1.00	3.73	2.84	C4orf19	chromosome 4 open reading frame 19
**NM_133424.1**	1.00	3.69	3.43	Actn3	actinin, alpha 3
**BF563467**	1.00	3.65	2.43	Ccdc92	coiled-coil domain containing 92
**BF419671**	1.00	3.64	6.59	Ay616753	glutaredoxin, cysteine rich 1
**AB000199**	1.00	3.56	4.23	Hsd3b7	hydroxy-delta-5-steroid dehydrogenase, 3 beta- and steroid delta-isomerase 7
**AI555341**	1.00	3.56	1.65	Null	UI-R-C2P-QW-E-02-0-UI.S1 UI-R-C2P
**AW918222**	1.00	3.48	2.79	Tor3a	torsin family 3, member A
**AA891949**	1.00	3.45	3.93	Ak3l1	adenylate kinase 3-like 2

Table S1: Aging

Accession	Young	Aged Control	Aged Treated	Symbol	Description
**NM_012974.1**	1.00	3.41	3.01	Lamb2	laminin, beta 2 (laminin S)
**NM_080782.1**	1.00	3.37	6.68	Cdkn1a	cyclin-dependent kinase inhibitor 1A (p21, Cip1)
**NM_024127.1**	1.00	3.36	8.23	Gadd45a	growth arrest and DNA-damage-inducible, alpha
**NM_017000.1**	1.00	3.27	2.58	Nqo1	NAD(P)H dehydrogenase, quinone 1
**NM_053922.1**	1.00	3.23	4.34	Acacb	acetyl-Coenzyme A carboxylase beta
**AA818377**	1.00	3.23	4.20	Znf385b	zinc finger protein 385B
**AI102517**	1.00	3.22	6.35	Null	EST211806 NORMALIZED RAT EMBRYO, BENTO SOARES
**NM_030875.1**	1.00	3.03	4.41	Scn1a	sodium channel, voltage-gated, type I, alpha subunit
**BF565344**	1.00	3.02	3.22	Cnnm1_predicted	cyclin M1
**NM_019298.1**	1.00	2.99	4.63	Chrnd	cholinergic receptor, nicotinic, delta
**BF412303**	1.00	2.99	2.30	Ppm1l	protein phosphatase 1 (formerly 2C)-like
**AI409738**	1.00	2.97	3.32	Null	EST238030 NORMALIZED RAT PLACENTA, BENTO SOARES
**M14050**	1.00	2.91	3.93	Hspa5	heat shock 70kDa protein 5 (glucose-regulated protein, 78kDa)
**NM_053601.1**	1.00	2.84	1.92	Nnat	neuronatin
**AI717140**	1.00	2.82	1.91	Tmem179	transmembrane protein 179
**NM_138519.1**	1.00	2.79	1.83	Dkk3	dickkopf homolog 3 (Xenopus laevis)
**NM_012861.1**	1.00	2.77	2.35	Mgmt	O-6-methylguanine-DNA methyltransferase
**NM_019315.1**	1.00	2.75	6.09	Kcnn3	potassium intermediate/small conductance calcium-activated channel, subfamily N, member 3
**NM_053669.1**	1.00	2.74	2.26	Sh2b2	SH2B adaptor protein 2
**BE113655**	1.00	2.68	1.79	Doc2g	double C2, gamma
**AI230548**	1.00	2.66	3.57	Ythdf2_predicted	YTH domain family, member 2
**NM_031143.1**	1.00	2.61	2.21	Dgkz	diacylglycerol kinase, zeta 104kDa
**NM_012874.1**	1.00	2.60	1.95	Ros1	c-ros oncogene 1 , receptor tyrosine kinase
**NM_030872.1**	1.00	2.56	2.79	Pdk2	pyruvate dehydrogenase kinase, isozyme 2
**AI011713**	1.00	2.53	3.85	Null	EST206164 NORMALIZED RAT OVARY, BENTO SOARES
**NM_017093.1**	1.00	2.53	4.13	Akt2	v-akt murine thymoma viral oncogene homolog 2
**BF288240**	1.00	2.50	3.87	Cdkn2b	cyclin-dependent kinase inhibitor 2B (p15, inhibits CDK4)
**BF404878**	1.00	2.48	1.02	Null	UI-R-CA1-BIC-G-03-0-UI.S1 UI-R-CA1
**AW143214**	1.00	2.47	3.14	Null	EST293510 NORMALIZED RAT BRAIN, BENTO SOARES
**NM_020082.1**	1.00	2.46	3.61	Rnase4	ribonuclease, RNase A family, 4
**NM_012543.1**	1.00	2.45	2.26	Dbp	D site of albumin promoter (albumin D-box) binding protein

Table S1: Aging

Accession	Young	Aged Control	Aged Treated	Symbol	Description
**AW914913**	1.00	2.44	1.62	Null	EST346217
**NM_053718.1**	1.00	2.41	4.37	Mllt3	myeloid/lymphoid or mixed-lineage leukemia
**AI639157**	1.00	2.40	1.44	Trap1	TNF receptor-associated protein 1
**AW524724**	1.00	2.39	5.81	Ryr3	ryanodine receptor 3
**NM_012827.1**	1.00	2.39	1.99	Bmp4	bone morphogenetic protein 4
**NM_017288.1**	1.00	2.39	2.14	Scn1b	sodium channel, voltage-gated, type I, beta
**AA943114**	1.00	2.38	3.21	Null	EST198613 NORMALIZED RAT BRAIN, BENTO SOARES
**AA943742**	1.00	2.37	1.81	Lsm3	LSM3 homolog, U6 small nuclear RNA associated (S. cerevisiae)
**NM_053608.1**	1.00	2.37	1.02	Kcnj13	potassium inwardly-rectifying channel, subfamily J, member 13
**BF407456**	1.00	2.35	2.57	Obfc1	oligonucleotide/oligosaccharide-binding fold containing 1
**NM_031819.1**	1.00	2.35	1.43	Fat	FAT tumor suppressor homolog 1 (Drosophila)
**NM_019205.1**	1.00	2.34	1.62	Ccl11	chemokine (C-C motif) ligand 11
**AW914045**	1.00	2.33	2.79	Eps8l2_predicted	EPS8-like 2
**NM_012846.1**	1.00	2.33	2.67	Fgf1	fibroblast growth factor 1 (acidic)
**BE100016**	1.00	2.29	1.82	Stard10	StAR-related lipid transfer (START) domain containing 10
**NM_019298.1**	1.00	2.28	2.84	Chrnd	cholinergic receptor, nicotinic, delta
**NM_012561.1**	1.00	2.27	3.75	Fst	follistatin
**BE107334**	1.00	2.26	3.17	Null	UI-R-BS1-AYS-E-09-0-UI.S1 UI-R-BS1
**NM_053977.1**	1.00	2.25	1.15	Cdh17	cadherin 17, LI cadherin (liver-intestine)
**AW917185**	1.00	2.25	2.43	Armet_predicted	arginine-rich, mutated in early stage tumors
**NM_024384.1**	1.00	2.25	2.25	Thoc6	THO complex 6 homolog (Drosophila)
**AJ277957**	1.00	2.24	2.37	Tcag7.1260	similar to aldo-keto reductase family 1, member B10
**AW919170**	1.00	2.23	2.69	Raly	RNA binding protein, autoantigenic (hnRNP-associated with lethal yellow homolog
**AW433866**	1.00	2.19	2.12	Null	UI-R-BJ0P-AFM-E-09-0-UI.S1 UI-R-BJ0P
**AI600036**	1.00	2.18	2.01	Null	EST251739 NORMALIZED RAT EMBRYO, BENTO SOARES
**NM_031712.1**	1.00	2.17	1.39	Pdzk1	PDZ domain containing 1
**AI230729**	1.00	2.17	2.15	Znf513	zinc finger protein 513
**NM_031971.1**	1.00	2.15	2.72	Hspa1b	heat shock 70kDa protein 1B
**AI137406**	1.00	2.15	1.77	Procr	protein C receptor, endothelial (EPCR)
**BF551173**	1.00	2.14	1.06	Null	UI-R-C1-JV-A-11-0-UI.R1 UI-R-C1
**NM_012627.1**	1.00	2.12	1.67	Pkib	protein kinase (cAMP-dependent, catalytic) inhibitor beta

Table S1: Aging

Accession	Young	Aged Control	Aged Treated	Symbol	Description
**NM_053612.1**	1.00	2.11	2.62	Hspb8	heat shock 22kDa protein 8
**AW143212**	1.00	2.11	2.24	Pfdn2	prefoldin subunit 2
**BF551250**	1.00	2.09	1.30	Fkbp5	FK506 binding protein 5
**AI013474**	1.00	2.09	3.43	Abhd2_predicted	abhydrolase domain containing 2
**NM_012531.1**	1.00	2.09	2.24	Comt	catechol-O-methyltransferase
**NM_020101.1**	1.00	2.09	1.96	Centa2	centaurin, alpha 2
**BE106888**	1.00	2.07	2.30	Creld2	cysteine-rich with EGF-like domains 2
**NM_030865.1**	1.00	2.07	2.05	Myoc	myocilin, trabecular meshwork inducible glucocorticoid response
**NM_013172.1**	1.00	2.07	3.14	Myf6	myogenic factor 6 (herculin)
**NM_031708.1**	1.00	2.06	1.73	Adrm1	adhesion regulating molecule 1
**BF558694**	1.00	2.05	3.17	Rab27a	RAB27A, member RAS oncogene family
**NM_017131.1**	1.00	2.02	2.93	Casq2	calsequestrin 2 (cardiac muscle)
**AI230728**	1.00	2.01	1.94	Snrpa	small nuclear ribonucleoprotein polypeptide A
**BF567803**	1.00	2.01	2.76	Null	UI-R-BO0-AHM-D-03-0-UI.R1 UI-R-BO0
**NM_057190.1**	1.00	2.01	1.05	Nelf	nasal embryonic LHRH factor
**NM_012778.1**	1.00	2.00	1.80	Aqp1	aquaporin 1 (Colton blood group)
**BE106058**	1.00	2.00	1.93	Null	UI-R-BO1-ASK-D-07-0-UI.S1 UI-R-BO1
**AA850544**	1.00	1.99	2.38	Nxx6-2	NK6 homeobox 2
**NM_017189.1**	1.00	1.98	2.18	Asgr2	asialoglycoprotein receptor 2
**BE114586**	1.00	1.96	2.50	Cdkn1a	cyclin-dependent kinase inhibitor 1A (p21, Cip1)
**AI010950**	1.00	1.96	2.19	Null	EST205401 NORMALIZED RAT MUSCLE, BENTO SOARES
**AF267197**	1.00	1.96	2.36	Tox4	TOX high mobility group box family member 4
**BE111776**	1.00	1.96	1.82	Null	UI-R-BJ1-AVX-F-10-0-UI.S1 UI-R-BJ1
**AW918650**	1.00	1.96	1.62	Chchd6_predicted	coiled-coil-helix-coiled-coil-helix domain containing 6
**D10699**	1.00	1.96	1.39	Uchl1	ubiquitin carboxyl-terminal esterase L1 (ubiquitin thiolesterase)
**U77880**	1.00	1.94	2.95	PDE7a	phosphodiesterase 7A
**AF112256**	1.00	1.94	2.28	Ryr1	ryanodine receptor 1, skeletal muscle
**AI102804**	1.00	1.94	1.99	Null	EST212093 NORMALIZED RAT EMBRYO, BENTO SOARES
**AI008969**	1.00	1.94	2.03	Null	EST203420 NORMALIZED RAT EMBRYO, BENTO SOARES
**H35261**	1.00	1.93	1.75	Null	EST104829 RAT PC-12 CELLS, UNTREATED
**NM_012604.1**	1.00	1.93	3.55	Myh3	myosin, heavy chain 3, skeletal muscle, embryonic

Table S1: Aging

Accession	Young	Aged Control	Aged Treated	Symbol	Description
**NM_017101.1**	1.00	1.93	1.97	Ppia	peptidylprolyl isomerase A (cyclophilin A)
**NM_133303.1**	1.00	1.91	1.75	Bhlhb3	basic helix-loop-helix domain containing, class B, 3
**AI233343**	1.00	1.90	1.47	Asrgl1	asparaginase like 1
**AI407409**	1.00	1.90	1.45	Xkr8	XK, Kell blood group complex subunit-related family, member 8
**AI408984**	1.00	1.89	1.89	Rfwd3	ring finger and WD repeat domain 3
**AI137488**	1.00	1.89	2.59	Pgrmc2	progesterone receptor membrane component 2
**AW143263**	1.00	1.87	2.12	Rich2	Rho-type GTPase-activating protein RICH2
**AI548760**	1.00	1.87	1.81	ngdn	neuroguidin, EIF4E binding protein
**AI007936**	1.00	1.85	1.47	Null	EST202387 NORMALIZED RAT BRAIN, BENTO SOARES
**NM_020106.1**	1.00	1.83	1.82	Or1n1	olfactory receptor, family 1, subfamily N, member 1
**BF285109**	1.00	1.83	1.37	Pck1	phosphoenolpyruvate carboxykinase 1 (soluble)
**AI104125**	1.00	1.83	1.14	Mrps27_predicted	mitochondrial ribosomal protein S27
**NM_031544.1**	1.00	1.82	3.74	Ampd3	adenosine monophosphate deaminase (isoform E)
**AA818999**	1.00	1.82	2.25	Loc680445	similar to muscleblind-like 2 isoform 1
**AA848958**	1.00	1.81	1.58	Null	EST191720 NORMALIZED RAT LUNG, BENTO SOARES
**AW252087**	1.00	1.80	2.83	Hspb7	heat shock 27kDa protein family, member 7 (cardiovascular)
**BF563077**	1.00	1.79	1.59	Null	UI-R-BO1-AIY-D-07-0-UI.R1
**NM_031677.1**	1.00	1.79	1.74	Fhl2	four and a half LIM domains 2
**NM_021863.1**	1.00	1.79	1.57	Hspa2	heat shock 70kDa protein 2
**AA892770**	1.00	1.78	2.62	Gclc	glutamate-cysteine ligase, catalytic subunit
**NM_012760.1**	1.00	1.78	2.26	Plagl1	pleiomorphic adenoma gene-like 1
**BF561727**	1.00	1.78	1.56	Null	UI-R-C0-IC-F-11-0-UI.R1
**AI232784**	1.00	1.77	-1.07	Bdh2_predicted	3-hydroxybutyrate dehydrogenase, type 2
**AW915009**	1.00	1.77	2.50	Null	EST346313
**AF411216**	1.00	1.76	2.94	Tmem49	transmembrane protein 49
**NM_017332.1**	1.00	1.76	-1.09	Fasn	fatty acid synthase
**AW917098**	1.00	1.76	2.17	Loc388135	chromosome 15 open reading frame 59
**AA946349**	1.00	1.76	1.49	Nudt3	nudix (nucleoside diphosphate linked moiety X)-type motif 3
**AW916940**	1.00	1.75	2.42	Null	EST348244
**AI410864**	1.00	1.75	1.06	Me3_predicted	malic enzyme 3, NADP(+)-dependent, mitochondrial
**AA945149**	1.00	1.75	3.03	Gstt3	glutathione S-transferase, theta 3

Table S1: Aging

Accession	Young	Aged Control	Aged Treated	Symbol	Description
**AI104235**	1.00	1.75	2.00	Sdf2l1_predicted	stromal cell-derived factor 2-like 1
**BF282282**	1.00	1.75	1.58	Psph	phosphoserine phosphatase
**NM_052807.1**	1.00	1.75	3.18	Igf1r	insulin-like growth factor 1 receptor
**BE116180**	1.00	1.74	1.65	Ylpm1	YLP motif containing 1
**NM_013168.1**	1.00	1.74	1.30	Hmbs	hydroxymethylbilane synthase
**AI502229**	1.00	1.73	1.80	Stau2	staufen, RNA binding protein, homolog 2 (Drosophila)
**AI600237**	1.00	1.73	1.70	Eef1e1_predicted	eukaryotic translation elongation factor 1 epsilon 1
**AI407490**	1.00	1.72	1.61	Yars	tyrosyl-tRNA synthetase
**NM_012848.1**	1.00	1.72	-1.46	Fth1	ferritin, heavy polypeptide 1
**AF199504**	1.00	1.72	1.72	Cblb	Cas-Br-M (murine) ecotropic retroviral transforming sequence b
**AA891839**	1.00	1.72	1.65	Mrpl50_predicted	mitochondrial ribosomal protein L50
**AW531805**	1.00	1.72	-1.32	Ifit3	interferon-induced protein with tetratricopeptide repeats 3
**AI407016**	1.00	1.72	2.34	Kiaa0152	KIAA0152
**NM_012811.1**	1.00	1.72	1.58	Mfge8	milk fat globule-EGF factor 8 protein
**M69056**	1.00	1.72	1.14	Fntb	farnesyltransferase, CAAX box, beta
**AI410050**	1.00	1.71	1.78	Med24	mediator complex subunit 24
**NM_032616.1**	1.00	1.71	1.17	Lsr	lipolysis stimulated lipoprotein receptor
**NM_133525.1**	1.00	1.71	-1.05	C6orf108	chromosome 6 open reading frame 108
**NM_031813.1**	1.00	1.71	4.74	Mybph	myosin binding protein H
**J05181**	1.00	1.71	2.22	Glclc	glutamate-cysteine ligase, catalytic subunit
**BE121438**	1.00	1.71	1.99	Null	UI-R-CA0-BAX-H-02-0-UI.S1 UI-R-CA0
**NM_030868.1**	1.00	1.71	1.84	Nov	nephroblastoma overexpressed gene
**NM_138504.1**	1.00	1.71	1.58	Osgin1	oxidative stress induced growth inhibitor 1
**AI411217**	1.00	1.70	3.34	Pycard	PYD and CARD domain containing
**AW918602**	1.00	1.70	2.18	Rbm20_predicted	RNA binding motif protein 20
**AW915236**	1.00	1.70	1.78	Atg2b	ATG2 autophagy related 2 homolog B (S. cerevisiae)
**AI411021**	1.00	1.70	1.46	Null	EST239315 NORMALIZED RAT KIDNEY, BENTO SOARES
**NM_019242.1**	1.00	1.69	2.60	Ifrd1	interferon-related developmental regulator 1
**NM_017288.1**	1.00	1.69	1.48	Scn1b	sodium channel, voltage-gated, type I, beta
**AW435315**	1.00	1.69	1.20	Gpr172b	G protein-coupled receptor 172B
**NM_031640.1**	1.00	1.69	2.51	Pgcp	plasma glutamate carboxypeptidase

Table S1: Aging

Accession	Young	Aged Control	Aged Treated	Symbol	Description
**AW918854**	1.00	1.69	1.41	Eya2	eyes absent homolog 2 (Drosophila)
**NM_024485.1**	1.00	1.69	3.64	Chrna1	cholinergic receptor, nicotinic, alpha 1 (muscle)
**NM_012661.1**	1.00	1.69	2.15	Sts	steroid sulfatase (microsomal), isozyme S
**NM_021699.1**	1.00	1.69	1.31	Mark2	MAP/microtubule affinity-regulating kinase 2
**NM_053799.1**	1.00	1.68	1.86	Dars	aspartyl-tRNA synthetase
**AI409108**	1.00	1.68	1.73	Null	EST237400 NORMALIZED RAT OVARY, BENTO SOARES
**U70050**	1.00	1.68	1.18	Jag2	jagged 2
**NM_032071.1**	1.00	1.68	-1.09	Synj2	synaptojanin 2
**NM_138548.1**	1.00	1.67	1.48	Nme1	non-metastatic cells 1, protein (NM23A) expressed in
**NM_012999.1**	1.00	1.67	1.46	Pcsk6	proprotein convertase subtilisin/kexin type 6
**U81160**	1.00	1.67	1.54	Vsp45a; MGC93104	vacuolar protein sorting 45 homolog (S. cerevisiae)
**AI412736**	1.00	1.66	1.65	Tomm34_predicted	translocase of outer mitochondrial membrane 34
**AW919062**	1.00	1.66	1.44	Null	EST350366 RAT GENE INDEX, NORMALIZED RAT, NORVEGICUS, BENTO SOARES
**AW918108**	1.00	1.66	2.03	Btaf1	BTAF1 RNA polymerase II, B-TFIID transcription factor-associated
**AA945771**	1.00	1.66	1.80	Null	EST201270 NORMALIZED RAT LUNG, BENTO SOARES
**AI176515**	1.00	1.66	2.38	Filip1	filamin A interacting protein 1
**AI231450**	1.00	1.66	1.79	LOC309957	similar to myocyte enhancer factor 2C
**AI407982**	1.00	1.66	2.25	Lrrfip1	leucine rich repeat (in FLII) interacting protein 1
**AI145869**	1.00	1.65	1.33	Tceal3	transcription elongation factor A (SII)-like 3
**AA946485**	1.00	1.65	6.16	Tgif1	TGFB-induced factor homeobox 1
**AA818571**	1.00	1.65	2.07	Null	UI-R-A0-AW-H-09-0-UI.S1 UI-R-A0
**AW520823**	1.00	1.65	1.56	Amotl1_predicted	angiomotin like 1
**NM_021578.1**	1.00	1.64	1.71	Tgfb1	transforming growth factor, beta 1
**NM_012939.1**	1.00	1.64	1.51	Ctsh	cathepsin H
**BF545958**	1.00	1.64	2.84	Sf3a1	splicing factor 3a, subunit 1, 120kDa
**AI233288**	1.00	1.64	1.71	Wipi1_predicted	WD repeat domain, phosphoinositide interacting 1
**NM_031139.1**	1.00	1.64	1.68	Usf2	upstream transcription factor 2, c-fos interacting
**AI105450**	1.00	1.63	1.91	C9orf3	chromosome 9 open reading frame 3
**BF393949**	1.00	1.63	1.77	Sec13	SEC13 homolog (S. cerevisiae)
**BF420447**	1.00	1.63	1.36	Null	UI-R-BJ2-BPW-E-12-0-UI.S1 UI-R-BJ2
**NM_017275.1**	1.00	1.63	1.35	Pnck	pregnancy up-regulated non-ubiquitously expressed CaM kinase

Table S1: Aging

Accession	Young	Aged Control	Aged Treated	Symbol	Description
**AW921399**	1.00	1.63	2.24	c5orf15	chromosome 5 open reading frame 15
**AI716516**	1.00	1.63	1.47	Null	UI-R-Y0-ABI-A-06-0-UI.S1 UI-R-Y0
**AJ245648**	1.00	1.63	1.52	Pola2	polymerase (DNA directed), alpha 2 (70kD subunit)
**M18336**	1.00	1.63	1.41	Cyp2c18	cytochrome P450, family 2, subfamily C, polypeptide 18
**AA944162**	1.00	1.63	1.69	Null	EST199661 NORMALIZED RAT EMBRYO, BENTO SOARES
**NM_021739.1**	1.00	1.62	2.22	Camk2b	calcium/calmodulin-dependent protein kinase (CaM kinase) II beta
**AI231792**	1.00	1.62	2.19	Bag3	BCL2-associated athanogene 3
**AA848834**	1.00	1.62	2.10	Dhdds	dehydrodolichyl diphosphate synthase
**BE107747**	1.00	1.62	1.29	Traf3ip3	TRAF3 interacting protein 3
**AW920818**	1.00	1.62	1.47	Pofut2_predicted	protein O-fucosyltransferase 2
**NM_012874.1**	1.00	1.62	1.16	Ros1	c-ros oncogene 1 , receptor tyrosine kinase
**NM_031967.1**	1.00	1.61	1.94	Ndrg4	NDRG family member 4
**NM_031814.1**	1.00	1.61	1.15	Git1	G protein-coupled receptor kinase interactor 1
**NM_031549.1**	1.00	1.61	-1.15	Tagln	transgelin
**AI407991**	1.00	1.61	1.72	Null	EST236281 NORMALIZED RAT PLACENTA, BENTO SOARES
**AF156878**	1.00	1.61	1.66	Ogfr	opioid growth factor receptor
**BE109381**	1.00	1.60	2.75	H6pd	hexose-6-phosphate dehydrogenase (glucose 1-dehydrogenase)
**BF555924**	1.00	1.60	1.66	Thap6	THAP domain containing 6
**AI230758**	1.00	1.60	1.06	M6prbp1	mannose-6-phosphate receptor binding protein 1
**AI008390**	1.00	1.60	2.42	Vash2	vasohibin 2
**NM_053959.1**	1.00	1.60	1.57	Bin1	bridging integrator 1
**AW143395**	1.00	1.59	1.24	Rcan2	regulator of calcineurin 2
**AW143082**	1.00	1.59	1.82	Null	EST293378
**AA818392**	1.00	1.59	1.52	Tnfaip8	tumor necrosis factor, alpha-induced protein 8
**M92340**	1.00	1.58	2.73	Il6st	interleukin 6 signal transducer (gp130, oncostatin M receptor)
**AA799515**	1.00	1.58	1.65	Wsb2	WD repeat and SOCS box-containing 2
**AI104857**	1.00	1.58	1.08	Ankrd37	ankyrin repeat domain 37
**BE096387**	1.00	1.58	1.80	LOC288526	similar to DNA segment on chromosome X and Y 155 expressed sequence isoform 1
**AW920343**	1.00	1.58	1.52	Null	EST351647 RAT GENE INDEX, NORMALIZED RAT, NORVEGICUS, BENTO SOARES
**AW915076**	1.00	1.57	1.93	Gpr146	G protein-coupled receptor 146
**BF283742**	1.00	1.57	1.68	Null	EST448333 RAT GENE INDEX, NORMALIZED RAT,

Table S1: Aging

Accession	Young	Aged Control	Aged Treated	Symbol	Description
**BF524281**	1.00	1.57	1.21	Null	UI-R-AG0-XA-D-02-0-UI.R1
**NM_013008.1**	1.00	1.57	-1.15	Pou1f1	POU class 1 homeobox 1
**AW918153**	1.00	1.57	1.75	Null	EST349457
**BE113005**	1.00	1.57	1.49	Null	UI-R-BJ1-AWF-C-10-0-UI.S1 UI-R-BJ1
**BE097102**	1.00	1.57	-1.07	Null	UI-R-BO1-APT-B-12-0-UI.S1 UI-R-BO1
**AW528864**	1.00	1.57	-1.79	Null	UI-R-BT1-AKJ-E-03-0-UI.S1 UI-R-BT1
**AW528874**	1.00	1.57	1.61	Hirip3	HIRA interacting protein 3
**AA818120**	1.00	1.56	2.12	Sln	sarcolipin
**AW920179**	1.00	1.55	1.61	Faim3	Fas apoptotic inhibitory molecule 3
**M61142**	1.00	1.55	1.43	Thop1	thimet oligopeptidase 1
**NM_019230.1**	1.00	1.55	2.38	Slc22a3	solute carrier family 22 (extraneuronal monoamine transporter), member 3
**NM_017346.1**	1.00	1.55	1.83	Cacnb1	calcium channel, voltage-dependent, beta 1 subunit
**NM_033230.1**	1.00	1.55	1.71	Akt1	v-akt murine thymoma viral oncogene homolog 1
**AW918441**	1.00	1.55	2.01	Null	EST349745
**AI232716**	1.00	1.55	1.14	Inmt	indolethylamine N-methyltransferase
**BF551283**	1.00	1.54	1.82	Them4	thioesterase superfamily member 4
**AA891944**	1.00	1.54	1.32	Null	EST195747 NORMALIZED RAT KIDNEY, BENTO SOARES
**BE101435**	1.00	1.54	1.81	Null	UI-R-BJ1-AUJ-A-02-0-UI.S1 UI-R-BJ1
**BE128566**	1.00	1.54	1.60	Copz2_predicted	coatomer protein complex, subunit zeta 2
**U78517**	1.00	1.54	1.50	Rapgef4	Rap guanine nucleotide exchange factor (GEF) 4
**AI101120**	1.00	1.54	-1.23	Null	EST210409 NORMALIZED RAT BRAIN, BENTO SOARES
**AB020022**	1.00	1.54	1.89	Prpf19	PRP19/PSO4 pre-mRNA processing factor 19 homolog (S. cerevisiae)
**BF568015**	1.00	1.54	1.81	Ebpl_predicted	emopamil binding protein-like
**AI411991**	1.00	1.54	1.59	Ndrg1	N-myc downstream regulated gene 1
**AW523642**	1.00	1.53	1.26	Suv420h1_predicted	suppressor of variegation 4-20 homolog 1 (Drosophila)
**AF059530**	1.00	1.53	2.29	Prmt3	protein arginine methyltransferase 3
**AA819192**	1.00	1.53	1.73	Null	UI-R-A0-AB-H-08-0-UI.S2 UI-R-A0
**AW253263**	1.00	1.53	1.82	Rtel1	regulator of telomere elongation helicase 1
**NM_017117.1**	1.00	1.53	1.58	Capn3	calpain 3, (p94)
**AW917674**	1.00	1.53	0.66	Prpf38b	PRP38 pre-mRNA processing factor 38 (yeast) domain containing B
**NM_053543.1**	1.00	1.52	1.58	Ncdn	neurochondrin

Table S1: Aging

Accession	Young	Aged Control	Aged Treated	Symbol	Description
**AI406310**	1.00	1.52	1.67	Null	EST234596 NORMALIZED RAT BRAIN, BENTO SOARES
**BF393902**	1.00	1.52	1.88	Rpia	ribose 5-phosphate isomerase A (ribose 5-phosphate epimerase)
**NM_080886.1**	1.00	1.52	1.77	Sc4mol	sterol-C4-methyl oxidase-like
**BE109665**	1.00	1.52	1.60	Null	UI-R-BJ1-AVQ-G-04-0-UI.S1 UI-R-BJ1
**BF420074**	1.00	1.52	1.01	Null	UI-R-BJ2-BPM-F-05-0-UI.S1 UI-R-BJ2
**NM_012591.1**	1.00	1.51	1.47	Irf1	interferon regulatory factor 1
**AW252152**	1.00	1.51	1.41	Null	UI-R-BJ0-AEE-G-12-0-UI.S1 UI-R-BJ0
**BF554744**	1.00	1.51	1.07	Lrg1	leucine-rich alpha-2-glycoprotein 1
**AI235252**	1.00	1.51	2.01	Null	EST231814 NORMALIZED RAT OVARY, BENTO SOARES
**AW918419**	1.00	1.51	1.18	1110012n22rik	RIKEN cDNA 1110012N22 gene
**BF283382**	1.00	1.50	1.95	Pgrmc2	progesterone receptor membrane component 2
**AA858572**	1.00	1.50	1.10	Loc100131801	similar to hCG2036585
**BE119991**	1.00	1.50	1.47	Frmpd1_predicted	FERM and PDZ domain containing 1
**NM_053947.1**	1.00	1.50	1.36	Mark1	MAP/microtubule affinity-regulating kinase 1
**BF550271**	1.00	1.49	1.53	Null	UI-R-A1-EC-F-09-0-UI.R1
**BE100771**	1.00	1.49	1.29	Tppp3	tubulin polymerization-promoting protein family member 3
**BF398684**	1.00	1.49	1.55	Null	UI-R-BS2-BER-F-08-0-UI.S1 UI-R-BS2
**BF388434**	1.00	1.48	2.05	Fam49b	family with sequence similarity 49, member B
**AA850317**	1.00	1.48	1.35	Ehd4	EH-domain containing 4
**AI180081**	1.00	1.48	1.24	Setd5	SET domain containing 5
**NM_032080.1**	1.00	1.48	2.18	Gsk3b	glycogen synthase kinase 3 beta
**AW915241**	1.00	1.48	1.64	Null	EST346545
**AW915499**	1.00	1.48	1.25	Vac14	Vac14 homolog (S. cerevisiae)
**NM_012498.1**	1.00	1.48	1.17	Akr1b1	aldo-keto reductase family 1, member B1 (aldose reductase)
**AI231309**	1.00	1.48	1.92	Aig1	androgen-induced 1
**AW915012**	1.00	1.48	1.37	Null	EST346316
**NM_013069.1**	1.00	1.47	1.78	Cd74	CD74 molecule, major histocompatibility complex, class II invariant chain
**BE100918**	1.00	1.47	1.89	Rufy1	RUN and FYVE domain containing 1
**NM_053563.1**	1.00	1.47	1.56	Ddx39	DEAD (Asp-Glu-Ala-Asp) box polypeptide 39
**NM_032079.1**	1.00	1.46	1.57	Dnaja2	DnaJ (Hsp40) homolog, subfamily A, member 2
**AA800364**	1.00	1.46	1.53	C19orf56	chromosome 19 open reading frame 56

Table S1: Aging

Accession	Young	Aged Control	Aged Treated	Symbol	Description
**BF282876**	1.00	1.46	1.50	Rab11fip3	RAB11 family interacting protein 3 (class II)
**NM_053929.1**	1.00	1.46	1.46	Slc7a9	solute carrier family 7 (cationic amino acid transporter, y+ system), member 9
**AI012460**	1.00	1.46	1.31	Ahcyl2	S-adenosylhomocysteine hydrolase-like 2
**AA891734**	1.00	1.46	2.20	Null	EST195537 NORMALIZED RAT KIDNEY, BENTO SOARES
**Y16774**	1.00	1.45	2.27	Slc30a4	solute carrier family 30 (zinc transporter), member 4
**AA818759**	1.00	1.45	1.65	Commd6	COMM domain containing 6
**Y15054**	1.00	1.45	-1.07	Coro7	coronin 7
**AI168965**	1.00	1.45	1.82	Fbxo28	F-box protein 28
**AI172302**	1.00	1.45	1.74	Srxn1	sulfiredoxin 1 homolog (S. cerevisiae)
**AI180337**	1.00	1.45	1.33	Cd2bp2_predicted	CD2 (cytoplasmic tail) binding protein 2
**AI030179**	1.00	1.45	-1.04	Atp6v0e2	ATPase, H+ transporting V0 subunit e2
**AW914939**	1.00	1.45	-1.32	Pqlc1	PQ loop repeat containing 1
**NM_019387.1**	1.00	1.44	1.53	Hgs	hepatocyte growth factor-regulated tyrosine kinase substrate
**BE098848**	1.00	1.44	1.41	Null	UI-R-BJ1-ATE-E-10-0-UI.S1 UI-R-BJ1
**AI227919**	1.00	1.44	1.42	Null	EST224614 NORMALIZED RAT BRAIN, BENTO SOARES
**BE109586**	1.00	1.44	1.38	Chmp6_predicted	chromatin modifying protein 6
**AA799476**	1.00	1.43	1.71	Txndc14	thioredoxin domain containing 14
**BF282415**	1.00	1.43	1.63	Null	EST446918 RAT GENE INDEX, NORMALIZED RAT,
**AW144039**	1.00	1.43	1.42	Txndc12	thioredoxin domain containing 12 (endoplasmic reticulum)
**BF564263**	1.00	1.43	1.44	Ift81	intraflagellar transport 81 homolog (Chlamydomonas)
**AI410895**	1.00	1.43	1.36	Null	EST239188 NORMALIZED RAT HEART, BENTO SOARES
**AI235446**	1.00	1.43	1.84	Null	EST232008 NORMALIZED RAT OVARY, BENTO SOARES
**AW441131**	1.00	1.42	1.69	Mycbp2	MYC binding protein 2
**AW916138**	1.00	1.42	1.56	Loc688393	similar to XPA binding protein 1
**AA859631**	1.00	1.42	1.34	Znf775	zinc finger protein 775
**AI010272**	1.00	1.42	1.71	Null	EST204723 NORMALIZED RAT LUNG, BENTO SOARES
**Z18877**	1.00	1.42	1.25	Oas1	2',5'-oligoadenylate synthetase 1, 40/46kDa
**AW915585**	1.00	1.42	-1.38	Cdc42ep1_predicted	CDC42 effector protein (Rho GTPase binding) 1
**AI233232**	1.00	1.41	1.73	Ccdc51	coiled-coil domain containing 51
**AI177089**	1.00	1.41	1.40	Gzf1	GDNF-inducible zinc finger protein 1
**AA892364**	1.00	1.41	1.62	Wbp11	WW domain binding protein 11

Table S1: Aging

Accession	Young	Aged Control	Aged Treated	Symbol	Description
**BE111296**	1.00	1.41	1.07	Tmcc2	transmembrane and coiled-coil domain family 2
**NM_017125.1**	1.00	1.40	1.70	Cd63	CD63 molecule
**AI175383**	1.00	1.40	1.48	Ccdc43	coiled-coil domain containing 43
**AW143855**	1.00	1.40	1.37	tmem167b	transmembrane protein 167B
**AI407930**	1.00	1.40	1.29	Pacsin2	protein kinase C and casein kinase substrate in neurons 2
**NM_012924.2**	1.00	1.40	1.99	Cd44	CD44 molecule (Indian blood group)
**NM_017236.1**	1.00	1.40	1.22	Pebp1	phosphatidylethanolamine binding protein 1
**NM_017076.1**	1.00	1.40	3.59	Pvr	poliovirus receptor
**BE113057**	1.00	1.40	1.61	Traf3	TNF receptor-associated factor 3
**AW918024**	1.00	1.39	1.72	Nif3l1	NIF3 NGG1 interacting factor 3-like 1 (S. pombe)
**AW918610**	1.00	1.39	1.58	Npepps	aminopeptidase puromycin sensitive
**AA943734**	1.00	1.39	1.24	Mtp18	mitochondrial protein 18 kDa
**NM_133419.1**	1.00	1.39	1.56	Dkc1	dyskeratosis congenita 1, dyskerin
**BE103359**	1.00	1.39	1.41	Kiaa0368	KIAA0368
**AI232065**	1.00	1.39	1.59	Arhgap18_predicted	Rho GTPase activating protein 18
**BF407203**	1.00	1.39	1.39	Gmpr2	guanosine monophosphate reductase 2
**BE096047**	1.00	1.39	1.33	Null	UI-R-BU0-APK-G-07-0-UI.S1 UI-R-BU0
**NM_017059.1**	1.00	1.38	1.85	Bax	BCL2-associated X protein
**BE100014**	1.00	1.38	1.26	Snx18	sorting nexin 18
**AA800241**	1.00	1.38	1.94	Arl6ip5	ADP-ribosylation-like factor 6 interacting protein 5
**NM_031051.1**	1.00	1.38	1.42	Mif	macrophage migration inhibitory factor (glycosylation-inhibiting factor)
**NM_019289.1**	1.00	1.38	1.23	Arpc1b	actin related protein 2/3 complex, subunit 1B, 41kDa
**NM_017274.1**	1.00	1.38	1.73	Gpam	glycerol-3-phosphate acyltransferase, mitochondrial
**AW917461**	1.00	1.38	1.12	Peli1	pellino homolog 1 (Drosophila)
**BI291916**	1.00	1.37	1.12	Null	UI-R-DN0-CIU-M-03-0-UI.S1
**BF289240**	1.00	1.37	1.22	Null	EST453831 RAT GENE INDEX, NORMALIZED RAT,
**BE103482**	1.00	1.37	1.06	Tbx1_predicted	T-box 1
**BE109520**	1.00	1.37	2.16	Fam134b	family with sequence similarity 134, member B
**NM_031337.1**	1.00	1.37	1.31	St3gal5	ST3 beta-galactoside alpha-2,3-sialyltransferase 5
**BE113312**	1.00	1.36	1.41	Ap3b1_predicted	adaptor-related protein complex 3, beta 1 subunit
**NM_019904.1**	1.00	1.36	1.25	Lgals1	lectin, galactoside-binding, soluble, 1 (galectin 1)

Table S1: Aging

Accession	Young	Aged Control	Aged Treated	Symbol	Description
**AA851241**	1.00	1.36	1.29	Brms1	breast cancer metastasis suppressor 1
**AI412090**	1.00	1.36	1.09	Flj14154	hypothetical protein FLJ14154
**NM_053373.1**	1.00	1.36	1.50	Pglyrp1	peptidoglycan recognition protein 1
**BG666918**	1.00	1.35	1.42	Gart	phosphoribosylglycinamide formyltransferase,
**AI029460**	1.00	1.35	1.01	Null	UI-R-C0-IR-G-06-0-UI.S1 UI-R-C0
**AI600037**	1.00	1.34	1.27	Galntl1	UDP-N-acetyl-alpha-D-galactosamine:polypeptide N-acetylgalactosaminyltransferase-like 1
**BF395080**	1.00	1.34	-2.09	Null	UI-R-CM0-BJI-E-05-0-UI.S1 UI-R-CM0
**AA800179**	1.00	1.34	1.55	Cox4nb	COX4 neighbor
**NM_053372.1**	1.00	1.34	1.18	Slpi	secretory leukocyte peptidase inhibitor
**BF284939**	1.00	1.34	1.13	C9orf69	chromosome 9 open reading frame 69
**NM_022266.1**	1.00	1.33	1.96	Ctgf	connective tissue growth factor
**AI232337**	1.00	1.33	1.10	Znf706	zinc finger protein 706
**AI412931**	1.00	1.32	1.67	Null	EST241231 NORMALIZED RAT KIDNEY, BENTO SOARES
**AW253642**	1.00	1.32	1.17	Ssna1_predicted	Sjogren syndrome nuclear autoantigen 1
**NM_024484.1**	1.00	1.32	1.34	Alas1	aminolevulinate, delta-, synthase 1
**BE108780**	1.00	1.31	1.22	Null	UI-R-BS1-AYY-D-02-0-UI.S1 UI-R-BS1
**AW916721**	1.00	-1.27	-1.24	Fam116a	family with sequence similarity 116, member A
**BF283250**	1.00	-1.31	1.05	Xylb	xylulokinase homolog (H. influenzae)
**AW143980**	1.00	-1.32	-1.19	Rgd1560888 Predicted	similar to Cell division protein kinase 8 (Protein kinase K35)
**AW525344**	1.00	-1.32	-1.38	Mnt_predicted	MAX binding protein
**AW530272**	1.00	-1.32	-1.67	Egfl8	EGF-like-domain, multiple 8
**NM_031773.1**	1.00	-1.32	-1.21	Polr1b	polymerase (RNA) I polypeptide B, 128kDa
**AI233194**	1.00	-1.33	-1.58	Null	EST229882 NORMALIZED RAT KIDNEY, BENTO SOARES
**AA875261**	1.00	-1.33	-1.86	Fblim1	filamin binding LIM protein 1
**BF282287**	1.00	-1.33	-1.22	Null	EST446966 RAT GENE INDEX, NORMALIZED RAT,
**NM_017142.1**	1.00	-1.33	-1.93	Adcy8	adenylate cyclase 8 (brain)
**BE106513**	1.00	-1.33	-1.36	Null	UI-R-BO1-ASO-H-06-0-UI.S1 UI-R-BO1
**BE098853**	1.00	-1.34	-1.92	Afap1l1	actin filament associated protein 1-like 1
**BE109108**	1.00	-1.34	-1.69	Patz1	POZ (BTB) and AT hook containing zinc finger 1
**NM_053820.1**	1.00	-1.34	-1.34	Ebf1	early B-cell factor 1
**AI407555**	1.00	-1.35	-1.60	Fbxw9	F-box and WD repeat domain containing 9

Table S1: Aging

Accession	Young	Aged Control	Aged Treated	Symbol	Description
**AW917738**	1.00	-1.35	-1.24	C21orf91	chromosome 21 open reading frame 91
**BE107737**	1.00	-1.35	-1.43	Crocc_predicted	ciliary rootlet coiled-coil, rootletin
**AI008952**	1.00	-1.35	-1.69	Eml4_predicted	echinoderm microtubule associated protein like 4
**NM_012816.1**	1.00	-1.35	-1.40	Amacr	alpha-methylacyl-CoA racemase
**BE098045**	1.00	-1.36	-1.37	Null	UI-R-BJ1-ASW-C-01-0-UI.S1 UI-R-BJ1
**BF549379**	1.00	-1.36	1.12	Null	UI-R-A0-BC-D-02-0-UI.R1
**NM_131907.1**	1.00	-1.36	1.30	Atp2c1	ATPase, Ca++ transporting, type 2C, member 1
**AI111863**	1.00	-1.36	-2.40	Null	UI-R-Y0-MP-F-02-0-UI.S1 UI-R-Y0
**NM_133619.1**	1.00	-1.37	-1.35	Gpha2	glycoprotein hormone alpha 2
**BF417386**	1.00	-1.37	-1.37	Null	UI-R-CN0-BLD-E-11-0-UI.S1 UI-R-CN0
**NM_013145.1**	1.00	-1.37	-1.61	Gnai1	guanine nucleotide binding protein (G protein), alpha inhibiting activity polypeptide 1
**NM_133618.1**	1.00	-1.37	1.37	Hadhb	hydroxyacyl-Coenzyme A dehydrogenase, beta subunit
**NM_016991.1**	1.00	-1.37	-1.65	Adra1b	adrenergic, alpha-1B-, receptor
**AI013475**	1.00	-1.37	-1.92	Sort1	sortilin 1
**NM_078619.1**	1.00	-1.38	-1.04	Slc8a2	solute carrier family 8 (sodium-calcium exchanger), member 2
**BE104961**	1.00	-1.38	-1.36	Cenpj_predicted	centromere protein J
**NM_133386.1**	1.00	-1.38	-1.45	Sphk1	sphingosine kinase 1
**M94040**	1.00	-1.38	-1.27	Bckdhb	branched chain keto acid dehydrogenase E1, beta polypeptide
**NM_022522.2**	1.00	-1.38	-1.03	Casp2	caspase 2, apoptosis-related cysteine peptidase
**NM_012588.1**	1.00	-1.38	-1.57	Igfbp3	insulin-like growth factor binding protein 3
**AI556402**	1.00	-1.38	-1.46	Null	UI-R-C2P-RH-B-09-0-UI.S1 UI-R-C2P
**BF396218**	1.00	-1.39	-1.42	C1orf131	chromosome 1 open reading frame 131
**NM_052808.1**	1.00	-1.39	-1.58	C20orf70	chromosome 20 open reading frame 70
**BF400766**	1.00	-1.39	-1.70	Lrrc46	leucine rich repeat containing 46
**AW919683**	1.00	-1.39	-1.88	Null	EST350987
**NM_053777.1**	1.00	-1.39	-1.85	Mapk8ip1	mitogen-activated protein kinase 8 interacting protein 1
**NM_019243.1**	1.00	-1.39	-1.08	Ptgfrn	prostaglandin F2 receptor negative regulator
**AW529588**	1.00	-1.39	-1.37	Null	UI-R-BT1-AKN-A-06-0-UI.S1 UI-R-BT1
**BF281969**	1.00	-1.39	-1.67	C10orf57	chromosome 10 open reading frame 57
**AI101373**	1.00	-1.39	-1.86	Zcchc24	zinc finger, CCHC domain containing 24
**NM_012896.1**	1.00	-1.39	-1.24	Adora3	adenosine A3 receptor

Table S1: Aging

Accession	Young	Aged Control	Aged Treated	Symbol	Description
**Z78279**	1.00	-1.39	-1.40	COLIA1	collagen, type I, alpha 1
**AI233752**	1.00	-1.40	-1.23	Slc4a11_predicted	solute carrier family 4, sodium borate transporter, member 11
**AW141192**	1.00	-1.40	-1.25	Tjp1_predicted	tight junction protein 1 (zona occludens 1)
**NM_080581.1**	1.00	-1.40	-1.77	Abcc3	ATP-binding cassette, sub-family C (CFTR/MRP), member 3
**BE111850**	1.00	-1.40	-1.44	Cnot7_predicted	CCR4-NOT transcription complex, subunit 7
**AI407500**	1.00	-1.40	-1.55	Mgc94725	similar to methionine adenosyltransferase II, beta
**AB019366**	1.00	-1.40	-1.35	Parg	poly (ADP-ribose) glycohydrolase
**AW144399**	1.00	-1.40	-1.21	C12orf35	chromosome 12 open reading frame 35
**BE111762**	1.00	-1.41	-1.36	Null	UI-R-BJ1-AVX-E-07-0-UI.S1 UI-R-BJ1
**NM_012651.1**	1.00	-1.41	-1.10	Slc4a1	solute carrier family 4, anion exchanger, member 1
**BF389120**	1.00	-1.41	-1.32	LOC302495	hypothetical LOC302495
**AI411225**	1.00	-1.42	-1.82	Itgav_predicted	integrin, alpha V (vitronectin receptor, alpha polypeptide, antigen CD51)
**NM_019136.1**	1.00	-1.42	-1.04	Avpr2	arginine vasopressin receptor 2 (nephrogenic diabetes insipidus)
**AA891858**	1.00	-1.42	-1.16	Cdh16	cadherin 16, KSP-cadherin
**NM_030838.1**	1.00	-1.42	-1.24	Slco1a5	solute carrier organic anion transporter family, member 1a5
**NM_017102.1**	1.00	-1.42	-1.27	Slc2a3	solute carrier family 2 (facilitated glucose transporter), member 3
**AI179236**	1.00	-1.42	-1.80	Null	EST222927 NORMALIZED RAT SPLEEN, BENTO SOARES
**NM_017145.1**	1.00	-1.43	-1.05	Mcpt1	mast cell protease 1
**NM_021751.1**	1.00	-1.43	-1.55	Prom1	prominin 1
**AI411077**	1.00	-1.43	-2.38	Zbtb5	zinc finger and BTB domain containing 5
**AA900400**	1.00	-1.43	-1.16	Wtap	Wilms tumor 1 associated protein
**U67139**	1.00	-1.43	-1.20	Dlgap3	discs, large (Drosophila) homolog-associated protein 3
**NM_133596.1**	1.00	-1.43	-1.41	Ugcgl1	UDP-glucose ceramide glucosyltransferase-like 1
**BF420720**	1.00	-1.43	-1.76	Arl5c	ADP-ribosylation factor-like 5C
**NM_021653.1**	1.00	-1.43	-1.25	Dio1	deiodinase, iodothyronine, type I
**AA892081**	1.00	-1.43	-1.71	C18orf19	chromosome 18 open reading frame 19
**AA858925**	1.00	-1.43	-1.81	Epb4.1l5	erythrocyte membrane protein band 4.1 like 5
**L09752**	1.00	-1.43	-1.64	Ccnd2	cyclin D2
**AI170763**	1.00	-1.43	-1.67	Agl_predicted	amylo-1, 6-glucosidase, 4-alpha-glucanotransferase
**AI178257**	1.00	-1.43	-1.71	Null	EST221922 NORMALIZED RAT PLACENTA, BENTO SOARES
**AI060055**	1.00	-1.43	-1.19	Uba6	ubiquitin-like modifier activating enzyme 6

Table S1: Aging

Accession	Young	Aged Control	Aged Treated	Symbol	Description
**AW535722**	1.00	-1.43	-1.42	Null	UI-R-BS0-AOD-C-06-0-UI.S1 UI-R-BS0
**NM_053623.1**	1.00	-1.44	-1.22	Acsl4	acyl-CoA synthetase long-chain family member 4
**BG673065**	1.00	-1.44	-1.33	Null	DRNBIE11 RAT DRG LIBRARY
**AW915681**	1.00	-1.44	-1.34	Ppapdc2	phosphatidic acid phosphatase type 2 domain containing 2
**AI406342**	1.00	-1.44	-1.18	Spock1	sparc/osteonectin, cwcv and kazal-like domains proteoglycan (testican) 1
**AI710879**	1.00	-1.44	-1.67	Null	UI-R-AE1-ZH-D-03-0-UI.S1 UI-R-AE1
**NM_013179.1**	1.00	-1.44	-1.27	Hcrt	hypocretin (orexin) neuropeptide precursor
**AF268593**	1.00	-1.44	-1.48	Itgam	integrin, alpha M (complement component 3 receptor 3 subunit)
**AW144391**	1.00	-1.44	-1.16	Pgm2l1	phosphoglucomutase 2-like 1
**NM_017063.1**	1.00	-1.44	-1.03	Kpnb1	karyopherin (importin) beta 1
**AI175048**	1.00	-1.44	-1.38	Six1	SIX homeobox 1
**NM_053477.1**	1.00	-1.45	-1.27	Mlycd	malonyl-CoA decarboxylase
**X69523**	1.00	-1.45	-1.43	Rbp3	retinol binding protein 3, interstitial
**AF387513**	1.00	-1.45	1.02	Bambi	BMP and activin membrane-bound inhibitor homolog
**NM_013173.1**	1.00	-1.45	-1.54	Slc11a2	solute carrier family 11 (proton-coupled divalent metal ion transporters), member 2
**AW919008**	1.00	-1.45	-1.39	Nudt8_predicted	nudix (nucleoside diphosphate linked moiety X)-type motif 8
**AA893184**	1.00	-1.45	-1.32	Pdhx	pyruvate dehydrogenase complex, component X
**NM_012816.1**	1.00	-1.45	-1.59	Amacr	alpha-methylacyl-CoA racemase
**AW254375**	1.00	-1.46	-1.48	Null	UI-R-BJ0-AEI-B-08-0-UI.S1 UI-R-BJ0
**NM_053758.1**	1.00	-1.46	-1.17	Plce1	phospholipase C, epsilon 1
**AI176848**	1.00	-1.46	-1.48	Null	EST220451 NORMALIZED RAT OVARY, BENTO SOARES
**AI408954**	1.00	-1.46	-1.03	Null	EST237245 NORMALIZED RAT KIDNEY, BENTO SOARES
**BE110545**	1.00	-1.46	-1.79	Osbp_predicted	oxysterol binding protein
**AA799358**	1.00	-1.46	-1.44	Null	EST188855 NORMALIZED RAT HEART, BENTO SOARES
**AI406341**	1.00	-1.46	-1.55	Null	EST234627 NORMALIZED RAT BRAIN, BENTO SOARES
**AW520781**	1.00	-1.46	-1.62	Asb8_predicted	ankyrin repeat and SOCS box-containing 8
**NM_134418.1**	1.00	-1.46	-1.23	Gp2	glycoprotein 2 (zymogen granule membrane)
**BE114154**	1.00	-1.46	-1.44	Null	UI-R-BJ1-AWJ-G-01-0-UI.S1 UI-R-BJ1
**BF282314**	1.00	-1.46	-1.48	C18orf8	chromosome 18 open reading frame 8
**AI411297**	1.00	-1.46	-1.81	Znf691	zinc finger protein 691
**BF284065**	1.00	-1.46	-2.03	Null	EST448656 RAT GENE INDEX, NORMALIZED RAT,

Table S1: Aging

Accession	Young	Aged Control	Aged Treated	Symbol	Description
**AI170067**	1.00	-1.46	-1.18	Null	EST215983 NORMALIZED RAT LUNG, BENTO SOARES
**AI578745**	1.00	-1.46	-1.74	Null	UI-R-AA0-WQ-F-11-0-UI.S1 UI-R-AA0
**NM_017027.1**	1.00	-1.46	-2.82	Mpz	myelin protein zero (Charcot-Marie-Tooth neuropathy 1B)
**BF567649**	1.00	-1.47	-1.42	Null	UI-R-BO0-AGS-G-07-0-UI.R1
**AI170769**	1.00	-1.47	-1.53	C9orf46	chromosome 9 open reading frame 46
**NM_057107.1**	1.00	-1.47	-1.18	Acsl3	acyl-CoA synthetase long-chain family member 3
**BF548006**	1.00	-1.47	-1.58	Anapc1_predicted	anaphase promoting complex subunit 1
**NM_017041.1**	1.00	-1.48	-1.13	Ppp3ca	protein phosphatase 3 (formerly 2B), catalytic subunit, alpha isoform
**BF556273**	1.00	-1.48	-1.65	Vezf1_predicted	vascular endothelial zinc finger 1
**Y00697.1**	1.00	-1.48	1.10	Ctsl2	cathepsin L2
**AF003598**	1.00	-1.48	-1.84	Itgb7	integrin, beta 7
**BF399633**	1.00	-1.48	-1.92	Kiaa0922	KIAA0922
**AI103954**	1.00	-1.48	-1.92	Null	EST213243 NORMALIZED RAT HEART, BENTO SOARES
**BF550302**	1.00	-1.48	-1.08	Null	UI-R-A1-EF-C-03-0-UI.R1 UI-R-A1
**NM_138506.1**	1.00	-1.48	-1.18	Adra2c	adrenergic, alpha-2C-, receptor
**AW144346**	1.00	-1.48	-1.57	Null	EST294642
**AA894233**	1.00	-1.49	-1.83	Aifm2	apoptosis-inducing factor, mitochondrion-associated, 2
**BE113034**	1.00	-1.49	-1.48	Isca1l	iron-sulfur cluster assembly 1 homolog (S. cerevisiae)-like
**BF564940**	1.00	-1.49	-1.80	Ifit2	interferon-induced protein with tetratricopeptide repeats 2
**NM_030998.1**	1.00	-1.49	-3.71	Amhr2	anti-Mullerian hormone receptor, type II
**AB052846**	1.00	-1.49	-1.57	Sc5dl	sterol-C5-desaturase (ERG3 delta-5-desaturase homolog, S. cerevisiae)-like
**NM_022533.1**	1.00	-1.49	-1.12	Pllp	plasma membrane proteolipid (plasmolipin)
**BF403712**	1.00	-1.49	-1.23	Zfyve1_predicted	zinc finger, FYVE domain containing 1
**NM_017110.1**	1.00	-1.49	-1.52	Cartpt	CART prepropeptide
**AA801220**	1.00	-1.49	-1.48	Null	EST190717 NORMALIZED RAT PLACENTA, BENTO SOARES
**BF523098**	1.00	-1.49	-1.22	Null	UI-R-C2P-RR-D-03-0-UI.R1 UI-R-C2P
**AW251401**	1.00	-1.49	-1.26	Null	UI-R-BJ0-ADH-G-01-0-UI.S1 UI-R-BJ0
**AW531891**	1.00	-1.49	-1.54	Bcl9	B-cell CLL/lymphoma 9
**NM_031693.1**	1.00	-1.49	-1.20	Syt4	synaptotagmin IV
**AW524559**	1.00	-1.49	-1.69	Null	UI-R-BO0-AHZ-G-12-0-UI.S1 UI-R-BO0
**NM_012621.1**	1.00	-1.49	-3.39	Pfkfb1	6-phosphofructo-2-kinase/fructose-2,6-biphosphatase 1

Table S1: Aging

Accession	Young	Aged Control	Aged Treated	Symbol	Description
**AI103645**	1.00	-1.50	-1.30	Melk_predicted	maternal embryonic leucine zipper kinase
**AI176838**	1.00	-1.50	-1.51	Null	EST220440 NORMALIZED RAT OVARY, BENTO SOARES
**BF395067**	1.00	-1.50	-2.07	Kiaa0922	KIAA0922
**AA997458**	1.00	-1.50	-1.31	Csrp2bp_predicted	CSRP2 binding protein
**D10655**	1.00	-1.50	-1.38	Dlat	dihydrolipoamide S-acetyltransferase
**AW918640**	1.00	-1.50	-1.86	Ivns1abp_predicted	influenza virus NS1A binding protein
**AW531735**	1.00	-1.50	-1.58	Null	UI-R-C4-ALK-E-03-0-UI.S1 UI-R-C4
**X53232**	1.00	-1.50	-1.87	Kiaa1688	KIAA1688 protein
**BE113966**	1.00	-1.50	-1.07	Gmfg	glia maturation factor, gamma
**AW918358**	1.00	-1.50	-1.15	C13orf1	chromosome 13 open reading frame 1
**BE113157**	1.00	-1.50	-1.52	Null	UI-R-BJ1-AWC-B-10-0-UI.S1 UI-R-BJ1
**AA817813**	1.00	-1.50	-1.64	Null	UI-R-A0-AE-C-09-0-UI.S1 UI-R-A0
**AA874838**	1.00	-1.50	1.36	Null	UI-R-E0-CG-G-03-0-UI.S1
**X13722**	1.00	-1.51	-1.22	LDLR	low density lipoprotein receptor (familial hypercholesterolemia)
**AA957047**	1.00	-1.51	-1.36	Loc302495	hypothetical LOC302495
**AW918376**	1.00	-1.51	-1.94	Loc680128	similar to phospholipase C-like 2
**NM_080481.1**	1.00	-1.51	-1.29	Atp5i	ATP synthase, H+ transporting, mitochondrial F0 complex, subunit E
**AA848536**	1.00	-1.51	-1.58	Null	EST191296 NORMALIZED RAT KIDNEY, BENTO SOARES
**BE118465**	1.00	-1.51	-1.39	Null	UI-R-BJ1-AZP-H-08-0-UI.S1 UI-R-BJ1
**AW915015**	1.00	-1.51	-1.45	Null	EST346319
**NM_012580.1**	1.00	-1.51	-1.04	Hmox1	heme oxygenase (decycling) 1
**NM_013090.1**	1.00	-1.51	-1.39	Vamp1	vesicle-associated membrane protein 1 (synaptobrevin 1)
**L19699**	1.00	-1.51	-1.68	Ralb	v-ral simian leukemia viral oncogene homolog B
**NM_013135.1**	1.00	-1.51	1.04	Rasa1	RAS p21 protein activator (GTPase activating protein) 1
**NM_053844.1**	1.00	-1.51	-1.42	Tff2	trefoil factor 2 (spasmolytic protein 1)
**NM_031573.1**	1.00	-1.51	-1.59	Phkg1	phosphorylase kinase, gamma 1 (muscle)
**U04933**	1.00	-1.52	-1.90	Slc8a1	solute carrier family 8 (sodium/calcium exchanger), member 1
**K02816**	1.00	-1.52	-1.47	Sub1	SUB1 homolog (S. cerevisiae)
**BE104321**	1.00	-1.52	-1.45	Ankrd13c	ankyrin repeat domain 13C
**BF400697**	1.00	-1.52	-1.61	Zrnb3	zinc finger, RAN-binding domain containing 3
**AA818602**	1.00	-1.52	-1.48	Rit2	Ras-like without CAAX 2

Table S1: Aging

Accession	Young	Aged Control	Aged Treated	Symbol	Description
**NM_017192.1**	1.00	-1.52	-1.56	S1pr2	sphingosine-1-phosphate receptor 2
**AI235934**	1.00	-1.52	-1.59	Jarid1b	jumonji, AT rich interactive domain 1B
**NM_053904.1**	1.00	-1.52	-2.22	Oplah	5-oxoprolinase (ATP-hydrolysing)
**BF282899**	1.00	-1.52	-1.31	Cdkn2c	cyclin-dependent kinase inhibitor 2C (p18, inhibits CDK4)
**BE109712**	1.00	-1.52	-1.94	Flj27352	hypothetical LOC145788
**BE113053**	1.00	-1.52	-1.78	Siat7F	ST6 -N-acetylgalactosaminide alpha-2,6-sialyltransferase 6
**BF288328**	1.00	-1.53	-1.16	Sp1	Sp1 transcription factor
**BF405581**	1.00	-1.53	-1.53	Phf3_predicted	PHD finger protein 3
**BF551118**	1.00	-1.53	-1.71	Sox17_predicted	SRY (sex determining region Y)-box 17
**AA925922**	1.00	-1.53	-1.47	Null	UI-R-A1-ES-G-09-0-UI.S1 UI-R-A1
**NM_019164.1**	1.00	-1.53	-1.22	HAD (Includes EG:1264	chondroadherin
**AI231460**	1.00	-1.53	-1.36	Null	EST228148 NORMALIZED RAT EMBRYO, BENTO SOARES
**NM_019237.1**	1.00	-1.53	-1.34	Pcolce	procollagen C-endopeptidase enhancer
**NM_013105.1**	1.00	-1.53	-1.57	Cyp3a4	cytochrome P450, family 3, subfamily A, polypeptide 4
**L14776**	1.00	-1.53	-1.78	Ctsj	cathepsin J
**NM_133585.1**	1.00	-1.53	-1.31	Opa1	optic atrophy 1 (autosomal dominant)
**NM_019239.1**	1.00	-1.53	-1.34	Mgat3	mannosyl (beta-1,4-)-glycoprotein beta-1,4-N-acetylglucosaminyltransferase
**AI407130**	1.00	-1.53	-1.63	Null	EST235418 NORMALIZED RAT OVARY, BENTO SOARES
**AI102799**	1.00	-1.53	-1.74	Vamp5	vesicle-associated membrane protein 5 (myobrevin)
**AA943126**	1.00	-1.54	-1.72	Fam115a	family with sequence similarity 115, member A
**AI176056**	1.00	-1.54	-2.33	Null	EST219632 NORMALIZED RAT OVARY, BENTO SOARES
**AW916756**	1.00	-1.54	-1.65	Null	EST348164
**BF389244**	1.00	-1.54	-1.57	Gins1	GINS complex subunit 1 (Psf1 homolog)
**AI101458**	1.00	-1.54	-1.63	Fam110b	family with sequence similarity 110, member B
**BE112781**	1.00	-1.54	-1.07	C10orf78	chromosome 10 open reading frame 78
**NM_133422.1**	1.00	-1.54	-1.23	Znf483	zinc finger protein 483
**M94043**	1.00	-1.54	-1.34	Rab38	RAB38, member RAS oncogene family
**NM_030860.1**	1.00	-1.54	-1.39	Mef2d	myocyte enhancer factor 2D
**AW254369**	1.00	-1.54	-1.46	Plekhb2_predicted	pleckstrin homology domain containing, family B (evectins) member 2
**AA800298**	1.00	-1.54	-1.98	Col15a1	collagen, type XV, alpha 1
**BE109604**	1.00	-1.54	-2.48	Otub2	OTU domain, ubiquitin aldehyde binding 2

Table S1: Aging

Accession	Young	Aged Control	Aged Treated	Symbol	Description
**D26113**	1.00	-1.55	-2.25	Fas	Fas (TNF receptor superfamily, member 6)
**AA957492**	1.00	-1.55	-1.31	Null	UI-R-E1-GB-E-01-0-UI.S1 UI-R-E1
**NM_019343.1**	1.00	-1.55	-1.39	Rgs7	regulator of G-protein signaling 7
**AI948410**	1.00	-1.55	-1.43	Null	612-7 RATTUS NORVEGICUS CDNA
**BG672160**	1.00	-1.55	-1.46	Tspan12	tetraspanin 12
**AI010267**	1.00	-1.55	-1.71	Null	EST204718 NORMALIZED RAT LUNG, BENTO SOARES
**AI411412**	1.00	-1.55	-1.46	Mosc1	MOCO sulphurase C-terminal domain containing 1
**BE099063**	1.00	-1.55	-1.75	Nradd	neurotrophin receptor associated death domain
**BE112921**	1.00	-1.55	-1.96	LOC500700	similar to chromosome 14 open reading frame 145
**AI235503**	1.00	-1.55	1.16	Null	EST232065 NORMALIZED RAT OVARY, BENTO SOARES
**BE111727**	1.00	-1.55	-1.70	Lass4_predicted	LAG1 homolog, ceramide synthase 4
**NM_053463.1**	1.00	-1.55	-2.21	Nucb1	nucleobindin 1
**BG666843**	1.00	-1.56	-1.44	Null	DRACNC05
**AW918105**	1.00	-1.56	-1.83	Strn	striatin, calmodulin binding protein
**AW915554**	1.00	-1.56	-1.19	Ascc3_predicted	activating signal cointegrator 1 complex subunit 3
**BF548520**	1.00	-1.56	-1.86	Null	UI-R-A0-AQ-B-01-0-UI.R1 UI-R-A0
**AI703715**	1.00	-1.56	-1.99	Prkd2	protein kinase D2
**BF409371**	1.00	-1.56	-1.93	Null	UI-R-CA1-BLF-C-06-0-UI.S1 UI-R-CA1
**BF288273**	1.00	-1.56	-1.38	Maml2	mastermind-like 2 (Drosophila)
**AW917849**	1.00	-1.56	-1.64	Null	EST349153
**BF400636**	1.00	-1.57	-1.60	Hspc159	galectin-related protein
**AI233916**	1.00	-1.57	-1.76	Hltf	helicase-like transcription factor
**AW915996**	1.00	-1.57	-2.47	Adamtsl2	ADAMTS-like 2
**NM_053346.1**	1.00	-1.57	-1.50	Nrn1	neuritin 1
**AI012474**	1.00	-1.57	-1.79	Agpat2_predicted	1-acylglycerol-3-phosphate O-acyltransferase 2
**X14788**	1.00	-1.57	-1.73	Creb1	cAMP responsive element binding protein 1
**AF230638**	1.00	-1.57	-1.33	Slc14a2	solute carrier family 14 (urea transporter), member 2
**NM_021850.1**	1.00	-1.57	-1.49	Bcl2l2	BCL2-like 2
**AW523888**	1.00	-1.57	-1.63	Null	UI-R-BO0-AIE-B-09-0-UI.S1 UI-R-BO0
**AI233865**	1.00	-1.57	-1.80	Null	EST230553 NORMALIZED RAT LUNG, BENTO SOARES
**AW527165**	1.00	-1.57	-1.96	Null	UI-R-BO1-AJM-C-06-0-UI.S1 UI-R-BO1

Table S1: Aging

Accession	Young	Aged Control	Aged Treated	Symbol	Description
**BF558902**	1.00	-1.57	-1.74	Nid2	nidogen 2 (osteonidogen)
**AI170351**	1.00	-1.57	-1.49	Dus4l_predicted	dihydrouridine synthase 4-like (S. cerevisiae)
**NM_019311.1**	1.00	-1.57	-1.54	Inpp5d	inositol polyphosphate-5-phosphatase, 145kDa
**NM_019296.1**	1.00	-1.57	-1.61	Cdc2	cell division cycle 2, G1 to S and G2 to M
**BF284067**	1.00	-1.57	-2.42	Rap1ga1	RAP1 GTPase activating protein
**AI112577**	1.00	-1.58	-1.41	Gng3	guanine nucleotide binding protein (G protein), gamma 3
**NM_053350.1**	1.00	-1.58	-1.56	Agps	alkylglycerone phosphate synthase
**NM_012797.1**	1.00	-1.58	-3.27	Id1	inhibitor of DNA binding 1, dominant negative helix-loop-helix protein
**AI413051**	1.00	-1.58	-1.48	Laptm4b	lysosomal associated protein transmembrane 4 beta
**AI317813**	1.00	-1.58	-1.63	Null	EST234484 PC12 CELLS, UNTREATED, PT7T3PAC, TIGR
**AW528454**	1.00	-1.58	-1.80	Isg20l1_predicted	apoptosis enhancing nuclease
**NM_053842.1**	1.00	-1.58	-1.50	Mapk1	mitogen-activated protein kinase 1
**BF391522**	1.00	-1.58	-1.31	Rnf139	ring finger protein 139
**NM_053404.1**	1.00	-1.58	-1.09	Dctn4	dynactin 4 (p62)
**NM_021701.1**	1.00	-1.58	-1.97	Ppp3r2	protein phosphatase 3 (formerly 2B), regulatory subunit B, beta isoform
**NM_130433.1**	1.00	-1.58	-2.38	Acaa2	acetyl-Coenzyme A acyltransferase 2
**BF394030**	1.00	-1.58	-1.34	Null	UI-R-CA0-BGY-B-01-0-UI.S1 UI-R-CA0
**AI179316**	1.00	-1.58	-1.78	Ankmy2_predicted	ankyrin repeat and MYND domain containing 2
**NM_031758.1**	1.00	-1.59	-1.37	Mchr1	melanin-concentrating hormone receptor 1
**AB005052**	1.00	-1.59	-1.28	Hip1r	huntingtin interacting protein 1 related
**AI227742**	1.00	-1.59	-1.68	Bok	BCL2-related ovarian killer
**AI176713**	1.00	-1.59	-2.75	Dlc1	deleted in liver cancer 1
**BE095842**	1.00	-1.60	1.14	Cyb561d1	cytochrome b-561 domain containing 1
**BF285164**	1.00	-1.60	-1.23	Popdc2	popeye domain containing 2
**AI411742**	1.00	-1.60	-1.65	Sesn1_predicted	sestrin 1
**AW531404**	1.00	-1.60	-2.05	Atpaf1	ATP synthase mitochondrial F1 complex assembly factor 1
**AW435463**	1.00	-1.60	-1.53	Null	UI-R-BJ0P-AFU-G-06-0-UI.S1 UI-R-BJ0P
**M33296**	1.00	-1.60	-1.76	Cyp2g1p	cytochrome P450, family 2, subfamily G, polypeptide 1 pseudogene
**BF410170**	1.00	-1.60	-1.76	Null	UI-R-CA1-BJU-C-11-0-UI.S1 UI-R-CA1
**AA858695**	1.00	-1.60	-1.80	Mrps33_predicted	mitochondrial ribosomal protein S33
**NM_012868.1**	1.00	-1.60	-3.00	Npr3	natriuretic peptide receptor C/guanylate cyclase C (atrionatriuretic peptide receptor C)

Table S1: Aging

Accession	Young	Aged Control	Aged Treated	Symbol	Description
**AA892273**	1.00	-1.60	-1.43	Rfx5	regulatory factor X, 5 (influences HLA class II expression)
**AI171651**	1.00	-1.61	-1.51	Tmem123	transmembrane protein 123
**BF282483**	1.00	-1.61	1.07	Null	EST447074 RAT GENE INDEX, NORMALIZED RAT,
**NM_053986.1**	1.00	-1.61	-1.66	Myo1b	myosin IB
**NM_031561.1**	1.00	-1.61	1.90	Cd36	CD36 molecule (thrombospondin receptor)
**AA894262**	1.00	-1.61	-1.45	Sept1	septin 1
**BF551328**	1.00	-1.61	-1.17	Klhl7	kelch-like 7 (Drosophila)
**NM_013015.1**	1.00	-1.61	-1.49	Ptgds	prostaglandin D2 synthase 21kDa (brain)
**AI406525**	1.00	-1.61	-2.09	Null	EST234811 NORMALIZED RAT BRAIN, BENTO SOARES
**NM_053310.1**	1.00	-1.62	-1.38	Homer3	homer homolog 3 (Drosophila)
**AW525071**	1.00	-1.62	-1.20	Slc44a4	solute carrier family 44, member 4
**AW142654**	1.00	-1.62	1.01	Null	EST292906 NORMALIZED RAT EMBRYO, BENTO SOARES
**BE107051**	1.00	-1.62	-1.47	Null	UI-R-BS1-AYP-C-04-0-UI.S1 UI-R-BS1
**AI044340**	1.00	-1.62	-1.68	Psrc1	proline/serine-rich coiled-coil 1
**AF079864**	1.00	-1.62	-1.96	Or51e2	olfactory receptor, family 51, subfamily E, member 2
**NM_012793.1**	1.00	-1.62	-2.02	Gamt	guanidinoacetate N-methyltransferase
**NM_013198.1**	1.00	-1.63	-1.50	Maob	monoamine oxidase B
**AI233257**	1.00	-1.63	-1.89	Null	EST229945 NORMALIZED RAT KIDNEY, BENTO SOARES
**BF550847**	1.00	-1.63	-1.71	NAJB3 (Includes EG:155	DnaJ (Hsp40) homolog, subfamily B, member 3
**AW915886**	1.00	-1.63	-1.37	Ptpla	protein tyrosine phosphatase-like , member A
**AI010423**	1.00	-1.63	-1.72	Null	EST204874 NORMALIZED RAT LUNG, BENTO SOARES
**AW915738**	1.00	-1.63	-1.60	Null	EST347042
**BE108249**	1.00	-1.63	-1.66	Ptpn14	protein tyrosine phosphatase, non-receptor type 14
**AA946474**	1.00	-1.63	-1.61	Cast	calpastatin
**AW253928**	1.00	-1.63	-1.78	Etv4_predicted	ets variant gene 4 (E1A enhancer binding protein, E1AF)
**BE107159**	1.00	-1.63	-1.78	Psip1	PC4 and SFRS1 interacting protein 1
**AW142369**	1.00	-1.64	-1.32	Heatr5b	HEAT repeat containing 5B
**BF409313**	1.00	-1.64	-1.66	Hddc2_predicted	HD domain containing 2
**BF386302**	1.00	-1.64	-1.78	Null	UI-R-CA1-BBC-H-10-0-UI.S1 UI-R-CA1
**BF414947**	1.00	-1.64	-1.79	Dusp14_predicted	dual specificity phosphatase 14
**BE110547**	1.00	-1.64	-2.06	Null	UI-R-BJ1-AVT-C-08-0-UI.S1 UI-R-BJ1

Table S1: Aging

Accession	Young	Aged Control	Aged Treated	Symbol	Description
**AW528057**	1.00	-1.64	-2.28	Null	UI-R-BT1-AKH-B-11-0-UI.S1 UI-R-BT1
**BF283694**	1.00	-1.64	-2.42	Null	EST448285 RAT GENE INDEX, NORMALIZED RAT,
**NM_012853.1**	1.00	-1.64	-1.43	Htr4	5-hydroxytryptamine (serotonin) receptor 4
**AW915107**	1.00	-1.64	-1.52	Null	EST346411
**BF282686**	1.00	-1.65	-1.78	Kiaa1826	KIAA1826
**BE113228**	1.00	-1.65	-2.19	C4orf29	chromosome 4 open reading frame 29
**NM_017032.1**	1.00	-1.65	-1.53	Pde4d	phosphodiesterase 4d, cAMP-specific
**AI598371**	1.00	-1.65	-1.75	Null	EST250074 NORMALIZED RAT EMBRYO, BENTO SOARES
**AI012390**	1.00	-1.65	-2.01	Null	EST206841 NORMALIZED RAT PLACENTA, BENTO SOARES
**NM_031559.1**	1.00	-1.65	-1.90	Cpt1a	carnitine palmitoyltransferase 1A (liver)
**AW917761**	1.00	-1.65	-2.50	Fam126b	family with sequence similarity 126, member B
**NM_052798.1**	1.00	-1.65	-1.28	Znf354a	zinc finger protein 354A
**BE102400**	1.00	-1.66	-1.54	Tln2	talin 2
**NM_031347.1**	1.00	-1.66	-1.76	Ppargc1a	peroxisome proliferator-activated receptor gamma, coactivator 1 alpha
**AW919132**	1.00	-1.66	-1.52	Null	EST350436
**BE113384**	1.00	-1.66	-1.61	Nsd1	nuclear receptor binding SET domain protein 1
**AA945750**	1.00	-1.66	-2.72	Null	EST201249 NORMALIZED RAT LUNG, BENTO SOARES
**NM_053321.1**	1.00	-1.66	-1.24	Ptafr	platelet-activating factor receptor
**BF398537**	1.00	-1.66	-1.50	Null	UI-R-BS2-BEP-H-11-0-UI.S1 UI-R-BS2
**AI407992**	1.00	-1.66	-1.71	Null	EST236282 NORMALIZED RAT PLACENTA, BENTO SOARES
**NM_012733.1**	1.00	-1.66	-1.23	Rbp1	retinol binding protein 1, cellular
**BF393884**	1.00	-1.66	-1.47	Null	UI-R-CA0-BGW-B-06-0-UI.S1 UI-R-CA0
**BF418913**	1.00	-1.67	-1.49	Wwtr1	WW domain containing transcription regulator 1
**BF545957**	1.00	-1.67	-1.78	Null	UI-R-C2P-RD-B-05-0-UI.R1 UI-R-C2P
**NM_017299.1**	1.00	-1.67	-1.63	Slc19a1	solute carrier family 19 (folate transporter), member 1
**NM_031601.1**	1.00	-1.67	-1.63	Cacna1g	calcium channel, voltage-dependent, T type, alpha 1G subunit
**AI180458**	1.00	-1.67	-1.39	Psme4	proteasome (prosome, macropain) activator subunit 4
**NM_022943.1**	1.00	-1.67	-2.04	Mertk	c-mer proto-oncogene tyrosine kinase
**U30789**	1.00	-1.67	1.30	Txnip	thioredoxin interacting protein
**AW141926**	1.00	-1.68	-1.08	Null	EST292041
**BF562347**	1.00	-1.68	-1.80	Null	UI-R-BU0-ANC-G-01-0-UI.R1

Table S1: Aging

Accession	Young	Aged Control	Aged Treated	Symbol	Description
**NM_017188.1**	1.00	-1.68	-2.49	Unc119	unc-119 homolog (C. elegans)
**AA891470**	1.00	-1.68	-1.73	Gpihbp1	glycosylphosphatidylinositol anchored high density lipoprotein binding protein 1
**BF282288**	1.00	-1.69	-1.33	Null	EST446967 RAT GENE INDEX, NORMALIZED RAT,
**AA998252**	1.00	-1.69	-1.57	Null	UI-R-C0-IE-F-02-0-UI.S1 UI-R-C0
**BF551318**	1.00	-1.69	1.61	Herc4	hect domain and RLD 4
**AI102009**	1.00	-1.69	-2.48	Prkra	protein kinase, interferon-inducible double stranded RNA dependent activator
**BE108201**	1.00	-1.69	-1.52	Ralgps2	Ral GEF with PH domain and SH3 binding motif 2
**NM_012659.1**	1.00	-1.69	-1.15	Sst	somatostatin
**AI233769**	1.00	-1.69	-1.79	Null	EST230457 NORMALIZED RAT KIDNEY, BENTO SOARES
**NM_053878.1**	1.00	-1.70	-1.75	Cplx2	complexin 2
**AI169596**	1.00	-1.70	-1.86	C15orf29	chromosome 15 open reading frame 29
**AA817752**	1.00	-1.70	-1.50	Snx2_predicted	sorting nexin 2
**AI178196**	1.00	-1.70	-1.84	Bahd1	bromo adjacent homology domain containing 1
**NM_013176.1**	1.00	-1.70	-1.26	Tcf12	transcription factor 12 (HTF4, helix-loop-helix transcription factors 4)
**AA946467**	1.00	-1.70	-1.99	Null	EST201966 NORMALIZED RAT OVARY, BENTO SOARES
**BE116848**	1.00	-1.71	-1.43	Null	UI-R-BS1-AZG-A-01-0-UI.S1 UI-R-BS1
**NM_080690.1**	1.00	-1.71	-1.45	Caskin1	CASK interacting protein 1
**AW915120**	1.00	-1.71	-1.42	Denndac	DENN/MADD domain containing 4C
**AI233199**	1.00	-1.71	-2.09	Null	EST229887 NORMALIZED RAT KIDNEY, BENTO SOARES
**AA963367**	1.00	-1.71	-1.59	Tm9sf3	transmembrane 9 superfamily member 3
**AF016047**	1.00	-1.72	-1.13	Pafah1b3	platelet-activating factor acetylhydrolase, isoform Ib, gamma subunit 29kDa
**AA859141**	1.00	-1.72	-1.27	Papd4	PAP associated domain containing 4
**AI555337**	1.00	-1.72	-1.22	Klhl11_predicted	kelch-like 11 (Drosophila)
**NM_012504.1**	1.00	-1.72	-2.16	Atp1a1	ATPase, Na+/K+ transporting, alpha 1 polypeptide
**NM_134453.1**	1.00	-1.72	-1.61	Lbr	lamin B receptor
**AA858649**	1.00	-1.72	-1.77	Vps36_predicted	vacuolar protein sorting 36 homolog (S. cerevisiae)
**X61677**	1.00	-1.72	-1.58	Itpr2	inositol 1,4,5-triphosphate receptor, type 2
**NM_013129.1**	1.00	-1.72	-1.72	Il15	interleukin 15
**NM_030994.1**	1.00	-1.73	-1.20	Itga1	integrin, alpha 1
**AI010721**	1.00	-1.73	-1.62	C14orf94	chromosome 14 open reading frame 94
**NM_030834.1**	1.00	-1.73	-2.12	Slc16a3	solute carrier family 16, member 3 (monocarboxylic acid transporter 4)

Table S1: Aging

Accession	Young	Aged Control	Aged Treated	Symbol	Description
**NM_017241.1**	1.00	-1.73	-2.43	Grik1	glutamate receptor, ionotropic, kainate 1
**AA849966**	1.00	-1.73	-2.00	Null	EST192733 NORMALIZED RAT MUSCLE, BENTO SOARES
**BF411381**	1.00	-1.73	-1.30	Null	UI-R-CN0-BMJ-G-04-0-UI.S1 UI-R-CN0
**AI555457**	1.00	-1.73	-1.86	Ctnnal1_predicted	catenin (cadherin-associated protein), alpha-like 1
**AI171975**	1.00	-1.73	-2.13	Sec24a	SEC24 related gene family, member A (S. cerevisiae)
**AI171775**	1.00	-1.73	-1.73	Null	EST217757 NORMALIZED RAT MUSCLE, BENTO SOARES
**BE108162**	1.00	-1.73	-1.80	Glcci1	glucocorticoid induced transcript 1
**AI599232**	1.00	-1.73	-2.44	Null	EST250935 NORMALIZED RAT EMBRYO, BENTO SOARES
**BF405996**	1.00	-1.74	-2.28	Cacna2d3	calcium channel, voltage-dependent, alpha 2/delta subunit 3
**NM_053965.1**	1.00	-1.74	-1.03	Slc25a20	solute carrier family 25 (carnitine/acylcarnitine translocase), member 20
**NM_012789.1**	1.00	-1.74	-1.66	Dpp4	dipeptidyl-peptidase 4
**BF410183**	1.00	-1.74	-1.86	Null	UI-R-CA1-BJU-D-12-0-UI.S1 UI-R-CA1
**AA892824**	1.00	-1.75	1.15	Tnc	tenascin C (hexabrachion)
**X76996**	1.00	-1.75	-1.77	Gzmb	granzyme B (granzyme 2, cytotoxic T-lymphocyte-associated serine esterase 1)
**NM_019216.1**	1.00	-1.75	-1.42	Gdf15	growth differentiation factor 15
**AI411809**	1.00	-1.75	-2.17	Null	EST240103 NORMALIZED RAT KIDNEY, BENTO SOARES
**AF030377**	1.00	-1.75	-1.19	Tie1	tyrosine kinase with immunoglobulin-like and EGF-like domains 1
**AA799503**	1.00	-1.75	-2.61	Null	EST189000 NORMALIZED RAT HEART, BENTO SOARES
**NM_080582.1**	1.00	-1.75	-1.70	Abcb6	ATP-binding cassette, sub-family B (MDR/TAP), member 6
**NM_054006.1**	1.00	-1.76	-1.75	Csde1	cold shock domain containing E1, RNA-binding
**BG671331**	1.00	-1.76	-2.29	Null	DRNBPB03
**M87067**	1.00	-1.76	-1.95	Acvr2b	activin A receptor, type IIB
**BE098855**	1.00	-1.76	-2.01	Ctdspl	CTD (carboxy-terminal domain) small phosphatase-like
**AW142170**	1.00	-1.76	-1.92	Rev3l	REV3-like, catalytic subunit of DNA polymerase zeta (yeast)
**AI070025**	1.00	-1.76	-1.44	Null	UI-R-C1-LN-B-09-0-UI.S1
**BE111638**	1.00	-1.76	-1.24	Galnt2_predicted	UDP-N-acetyl-alpha-D-galactosamine:polypeptide N-acetylgalactosaminyltransferase 2
**NM_080888.1**	1.00	-1.76	-1.65	Bnip3l	BCL2/adenovirus E1B 19kDa interacting protein 3-like
**AI178923**	1.00	-1.77	-1.94	Glcci1	glucocorticoid induced transcript 1
**BF281544**	1.00	-1.77	-1.90	Reln	reelin
**AW917390**	1.00	-1.77	-1.38	Null	EST348694
**AI406896**	1.00	-1.77	-1.61	c19orf2	chromosome 19 open reading frame 2

Table S1: Aging

Accession	Young	Aged Control	Aged Treated	Symbol	Description
**AW532687**	1.00	-1.78	-1.26	Null	UI-R-BS0-AMK-H-11-0-UI.S1 UI-R-BS0
**AI407827**	1.00	-1.78	-1.21	Pik3cd_predicted	phosphoinositide-3-kinase, catalytic, delta polypeptide
**NM_017161.1**	1.00	-1.78	-1.59	Adora2b	adenosine A2b receptor
**AI598307**	1.00	-1.78	-2.73	Slc45a3_predicted	solute carrier family 45, member 3
**AW251360**	1.00	-1.78	-2.11	C1qtnf6	C1q and tumor necrosis factor related protein 6
**NM_053605.1**	1.00	-1.79	-2.89	Smpd3	sphingomyelin phosphodiesterase 3, neutral membrane
**AI411227**	1.00	-1.79	-2.28	Atrnl1	attractin-like 1
**BF404027**	1.00	-1.79	-2.12	Null	UI-R-CA1-BJD-I-24-0-UI.S1 UI-R-CA1
**AI412662**	1.00	-1.79	-2.05	Efnb3_predicted	ephrin-B3
**NM_016986.1**	1.00	-1.80	-2.61	Acadm	acyl-Coenzyme A dehydrogenase, C-4 to C-12 straight chain
**AW523755**	1.00	-1.80	-2.16	Ppp1r1a	protein phosphatase 1, regulatory (inhibitor) subunit 1A
**BF405086**	1.00	-1.80	-1.61	Null	UI-R-CA1-BIP-D-11-0-UI.S1 UI-R-CA1
**AW252878**	1.00	-1.80	-1.66	Pxdn	peroxidasin homolog (Drosophila)
**BF550401**	1.00	-1.80	-1.95	Cct5	chaperonin containing TCP1, subunit 5 (epsilon)
**NM_019238.1**	1.00	-1.80	-2.35	Fdft1	farnesyl-diphosphate farnesyltransferase 1
**AI172271**	1.00	-1.80	-2.70	Emcn	endomucin
**BF290076**	1.00	-1.80	-1.84	Gem_predicted	GTP binding protein overexpressed in skeletal muscle
**AI231444**	1.00	-1.80	-2.06	Loc289233	similar to Peroxisomal biogenesis factor 19 (Peroxin-19)
**NM_053619.1**	1.00	-1.80	-2.00	C5ar1	complement component 5a receptor 1
**BF283084**	1.00	-1.81	-1.76	Sftd2	SFT2 domain containing 2
**BF567872**	1.00	-1.81	-1.39	Null	UI-R-BO0-AHO-B-09-0-UI.R1
**NM_012504.1**	1.00	-1.81	-2.08	Atp1a1	ATPase, Na+/K+ transporting, alpha 1 polypeptide
**AW918182**	1.00	-1.81	-1.54	Null	EST349486 RAT GENE INDEX, NORMALIZED RAT, NORVEGICUS, BENTO SOARES
**AA799329**	1.00	-1.81	-1.92	Fam111a	family with sequence similarity 111, member A
**BF403009**	1.00	-1.81	-1.92	Null	UI-R-CA0-BHL-D-08-0-UI.S1 UI-R-CA0
**NM_017216.1**	1.00	-1.81	-2.01	Slc3a1	solute carrier family 3, member 1
**BF404539**	1.00	-1.81	-1.59	Null	UI-R-CA1-BIG-B-12-0-UI.S1 UI-R-CA1
**AI412002**	1.00	-1.81	-1.87	Trnt1	tRNA nucleotidyl transferase, CCA-adding, 1
**BF402407**	1.00	-1.81	1.02	Rhobtb2	Rho-related BTB domain containing 2
**NM_019305.1**	1.00	-1.82	-1.48	Fgf2	fibroblast growth factor 2 (basic)
**BF405932**	1.00	-1.82	-1.74	Null	UI-R-CA1-BII-G-01-0-UI.S1 UI-R-CA1

Table S1: Aging

Accession	Young	Aged Control	Aged Treated	Symbol	Description
**AA858817**	1.00	-1.83	-2.86	C10orf10	chromosome 10 open reading frame 10
**AW918541**	1.00	-1.83	-2.20	Null	EST349845
**NM_031115.1**	1.00	-1.83	-1.40	Sctr	secretin receptor
**BE109637**	1.00	-1.83	-3.32	Fn3k	fructosamine 3 kinase
**M81639**	1.00	-1.83	-2.06	Snn	stannin
**NM_012511.1**	1.00	-1.83	-1.29	Atp7b	ATPase, Cu++ transporting, beta polypeptide
**AW528971**	1.00	-1.83	-1.20	Traf3ip2	TRAF3 interacting protein 2
**AF002251**	1.00	-1.83	-1.81	Rassf5	Ras association (RalGDS/AF-6) domain family member 5
**AW920324**	1.00	-1.83	-1.88	Fat4	FAT tumor suppressor homolog 4 (Drosophila)
**NM_019186.1**	1.00	-1.84	-1.74	Arl4a	ADP-ribosylation factor-like 4A
**NM_031663.1**	1.00	-1.84	-1.95	Slc18a3	solute carrier family 18 (vesicular acetylcholine), member 3
**BE103440**	1.00	-1.84	-1.98	Null	UI-R-BX0-ARC-B-03-0-UI.S1 UI-R-BX0
**BF565001**	1.00	-1.84	-1.57	Pde4d	phosphodiesterase 4d, cAMP-specific
**BE115519**	1.00	-1.85	-1.42	Sox4	SRY (sex determining region Y)-box 4
**NM_012780.1**	1.00	-1.85	-1.47	Arnt	aryl hydrocarbon receptor nuclear translocator
**AI012456**	1.00	-1.85	-1.48	Spcs3	signal peptidase complex subunit 3 homolog (S. cerevisiae)
**AW919696**	1.00	-1.85	-1.98	Null	EST351000
**NM_021669.1**	1.00	-1.85	-1.87	Ghrl	ghrelin/obestatin prepropeptide
**BF284887**	1.00	-1.85	-1.90	C20orf194	chromosome 20 open reading frame 194
**AI113104**	1.00	-1.85	-2.01	Prc1_predicted	protein regulator of cytokinesis 1
**AI146056**	1.00	-1.86	-1.20	Nxph3	neurexophilin 3
**AI235610**	1.00	-1.86	-1.57	Null	EST232172 NORMALIZED RAT OVARY, BENTO SOARES
**L38615**	1.00	-1.86	-1.53	Gss	glutathione synthetase
**BF390003**	1.00	-1.87	-1.50	C7orf60	chromosome 7 open reading frame 60
**AI011455**	1.00	-1.87	-1.31	Null	EST205906 NORMALIZED RAT OVARY, BENTO SOARES
**AI555237**	1.00	-1.87	-1.43	Null	UI-R-C2P-QU-H-11-0-UI.S1 UI-R-C2P
**AW915491**	1.00	-1.87	-1.18	Cep170	centrosomal protein 170kDa
**BG671716**	1.00	-1.87	-1.80	Nudt7_predicted	nudix (nucleoside diphosphate linked moiety X)-type motif 7
**BF394170**	1.00	-1.87	-2.08	Jdp2	Jun dimerization protein 2
**AA850909**	1.00	-1.88	-2.25	Pvrl2	poliovirus receptor-related 2 (herpesvirus entry mediator B)
**BE113264**	1.00	-1.88	-1.87	Osbpl9_predicted	oxysterol binding protein-like 9

Table S1: Aging

Accession	Young	Aged Control	Aged Treated	Symbol	Description
**NM_017092.1**	1.00	-1.88	-1.32	Tyro3	TYRO3 protein tyrosine kinase
**BE113599**	1.00	-1.88	-1.64	Null	UI-R-BJ1-AWG-B-09-0-UI.S1 UI-R-BJ1
**AI176950**	1.00	-1.88	-1.87	Null	EST220556 NORMALIZED RAT OVARY, BENTO SOARES
**BF396317**	1.00	-1.88	-2.31	Null	UI-R-BS2-BDW-G-05-0-UI.S1 UI-R-BS2
**BF288765**	1.00	-1.88	-1.54	Null	EST453266 RAT GENE INDEX, NORMALIZED RAT,
**BF407675**	1.00	-1.89	-1.88	Null	UI-R-BJ2-BQV-A-11-0-UI.S1 UI-R-BJ2
**NM_013187.1**	1.00	-1.89	-2.59	Plcg1	phospholipase C, gamma 1
**AI600255**	1.00	-1.89	-1.74	C10orf26	chromosome 10 open reading frame 26
**AW531361**	1.00	-1.90	-2.24	Pnmal2	PNMA-like 2
**NM_012944.1**	1.00	-1.90	-1.38	Drd4	dopamine receptor D4
**AW532214**	1.00	-1.90	-1.44	Null	UI-R-C4-ALJ-G-04-0-UI.S1 UI-R-C4
**AI010432**	1.00	-1.90	-2.42	Ppp1r3d	protein phosphatase 1, regulatory (inhibitor) subunit 3D
**NM_022936.1**	1.00	-1.91	-3.55	Ephx2	epoxide hydrolase 2, cytoplasmic
**BF389882**	1.00	-1.91	-1.64	Shprh_predicted	SNF2 histone linker PHD RING helicase
**NM_019303.1**	1.00	-1.91	-2.70	Cyp2f1	cytochrome P450, family 2, subfamily F, polypeptide 1
**NM_012702.1**	1.00	-1.91	-1.97	CAM3 (Includes EG:384	carcinoembryonic antigen-related cell adhesion molecule 3
**NM_019295.1**	1.00	-1.91	-2.74	Cd5	CD5 molecule
**AI177379**	1.00	-1.92	-1.69	Null	EST220999 NORMALIZED RAT PLACENTA, BENTO SOARES
**AI179315**	1.00	-1.92	-2.28	B3gnt2	UDP-GlcNAc:betaGal beta-1,3-N-acetylglucosaminyltransferase 2
**BF564049**	1.00	-1.92	-1.38	Null	UI-R-C4-AKY-A-09-0-UI.R1
**AA955175**	1.00	-1.92	-3.02	Null	UI-R-A1-DU-B-04-0-UI.S1 UI-R-A1
**NM_053988.1**	1.00	-1.93	-1.07	Calb2	calbindin 2
**AI227924**	1.00	-1.93	-1.82	Purg_predicted	purine-rich element binding protein G
**BF550413**	1.00	-1.93	-1.70	Null	UI-R-A1-EG-F-07-0-UI.R1
**AI412740**	1.00	-1.93	-2.18	Null	EST241039 NORMALIZED RAT BRAIN, BENTO SOARES
**NM_031977.1**	1.00	-1.93	-2.04	Src	v-src sarcoma (Schmidt-Ruppin A-2) viral oncogene homolog (avian)
**AA946375**	1.00	-1.94	-1.58	Smc5l1_predicted	structural maintenance of chromosomes 5
**Z83035**	1.00	-1.94	-1.64	C10orf26	chromosome 10 open reading frame 26
**BF407916**	1.00	-1.94	-2.04	Gdf11	growth differentiation factor 11
**AF205635**	1.00	-1.94	-1.20	Cdc42	cell division cycle 42 pseudogene
**BF285068**	1.00	-1.96	-2.06	Ciapin1	cytokine induced apoptosis inhibitor 1

Table S1: Aging

Accession	Young	Aged Control	Aged Treated	Symbol	Description
**AI009530**	1.00	-1.96	-1.91	C4orf18	chromosome 4 open reading frame 18
**U73503**	1.00	-1.96	-1.60	Camk2g	calcium/calmodulin-dependent protein kinase (CaM kinase) II gamma
**U61729**	1.00	-1.97	-1.69	Pnrc1	proline-rich nuclear receptor coactivator 1
**BF289154**	1.00	-1.97	-1.91	Null	EST453745 RAT GENE INDEX, NORMALIZED RAT,
**AI575445**	1.00	-1.98	-3.69	Ccdc65	coiled-coil domain containing 65
**AI176781**	1.00	-1.98	-2.31	Null	EST220376 NORMALIZED RAT OVARY, BENTO SOARES
**AI406984**	1.00	-1.99	-3.22	Null	EST235272 NORMALIZED RAT OVARY, BENTO SOARES
**NM_053403.1**	1.00	-1.99	-1.75	Grb7	growth factor receptor-bound protein 7
**AA858930**	1.00	-2.00	-2.16	Pde4b	phosphodiesterase 4b, cAMP-specific
**BF282381**	1.00	-2.00	-1.97	Null	EST446884 RAT GENE INDEX, NORMALIZED RAT,
**AA892496**	1.00	-2.01	-1.37	Null	EST196299 NORMALIZED RAT KIDNEY, BENTO SOARES
**NM_024158.1**	1.00	-2.02	-1.41	Dck	deoxycytidine kinase
**AW143149**	1.00	-2.03	-1.85	Gnpda2_predicted	glucosamine-6-phosphate deaminase 2
**BF283760**	1.00	-2.03	-1.93	Ldb2_predicted	LIM domain binding 2
**AI411194**	1.00	-2.04	-1.55	Pnpla2_predicted	patatin-like phospholipase domain containing 2
**BG673439**	1.00	-2.05	-2.46	Cldn11	claudin 11 (oligodendrocyte transmembrane protein)
**NM_019372.1**	1.00	-2.06	-2.32	Ppm2c	protein phosphatase 2C, magnesium-dependent, catalytic subunit
**AI171772**	1.00	-2.07	-1.65	Rp5-1022p6.2	hypothetical protein KIAA1434
**AI230591**	**1.00**	**-2.07**	**-1.32**	Ctla2a	**cytotoxic T lymphocyte-associated protein 2 alpha**
**BE108405**	1.00	-2.08	-1.69	Null	UI-R-BS1-AYV-H-06-0-UI.S1 UI-R-BS1
**AA892554**	1.00	-2.08	-2.10	G3bp2	GTPase activating protein (SH3 domain) binding protein 2
**AW915638**	1.00	-2.09	-2.26	Mdfic	MyoD family inhibitor domain containing
**BF556836**	1.00	-2.09	-1.68	Btbd16	BTB (POZ) domain containing 16
**BG670878**	1.00	-2.09	-1.68	Null	DRNBHH09
**AI410818**	1.00	-2.09	-3.80	Null	EST239111 NORMALIZED RAT HEART, BENTO SOARES
**BE111650**	1.00	-2.10	-1.80	Garnl1	GTPase activating Rap/RanGAP domain-like 1
**AI599484**	1.00	-2.11	-2.45	Loc1001308	similar to hCG2030844
**BF395777**	1.00	-2.12	-2.29	Null	UI-R-BT1-BKC-B-10-0-UI.S1 UI-R-BT1
**NM_133387.1**	1.00	-2.12	-2.34	Tmlhe	trimethyllysine hydroxylase, epsilon
**NM_012612.1**	1.00	-2.12	-1.86	Nppa	natriuretic peptide precursor A
**AI511275**	1.00	-2.13	-2.70	Null	UI-R-C3-SV-C-03-0-UI.S1 UI-R-C3

Table S1: Aging

Accession	Young	Aged Control	Aged Treated	Symbol	Description
**BF523712**	1.00	-2.13	-2.12	Null	UI-R-Y0-VB-B-03-0-UI.R1
**BF283600**	1.00	-2.14	-2.63	Papss2_predicted	3'-phosphoadenosine 5'-phosphosulfate synthase 2
**BE104373**	1.00	-2.14	-2.40	Null	UI-R-BX0-ARO-B-01-0-UI.S1 UI-R-BX0
**NM_057127.1**	1.00	-2.14	-2.20	Slc26a2	solute carrier family 26 (sulfate transporter), member 2
**AI639139**	1.00	-2.15	-1.67	Null	RX04483S RAT MIXED-TISSUE LIBRARY
**NM_012972.1**	1.00	-2.15	-2.45	Kcna5	potassium voltage-gated channel, shaker-related subfamily, member 5
**AI705731**	1.00	-2.16	-1.94	Mtus1	mitochondrial tumor suppressor 1
**AI412192**	1.00	-2.16	-2.65	Sar1b	SAR1 gene homolog B (S. cerevisiae)
**NM_138511.1**	1.00	-2.16	-1.96	Gpc2	glypican 2
**AI044229**	1.00	-2.17	-1.43	Cul4b_predicted	cullin 4B
**AW254017**	1.00	-2.17	-1.90	Col5a1	collagen, type V, alpha 1
**BE103689**	1.00	-2.18	-1.83	Zfhx4_predicted	zinc finger homeobox 4
**AW252871**	1.00	-2.18	-1.68	Mki67_predicted	antigen identified by monoclonal antibody Ki-67
**BE099629**	1.00	-2.18	-1.98	C3orf26	chromosome 3 open reading frame 26
**BF406522**	1.00	-2.19	-2.63	Cdr2	cerebellar degeneration-related protein 2, 62kDa
**BF393863**	1.00	-2.19	-2.46	Chd3	chromodomain helicase DNA binding protein 3
**AI233702**	1.00	-2.19	-2.22	Cmbl	carboxymethylenebutenolidase homolog (Pseudomonas)
**NM_053580.1**	1.00	-2.20	-1.86	Slc27a1	solute carrier family 27 (fatty acid transporter), member 1
**NM_053535.1**	1.00	-2.20	-2.20	Enpp1	ectonucleotide pyrophosphatase/phosphodiesterase 1
**X00469**	1.00	-2.22	-1.56	Cyp1a1	cytochrome P450, family 1, subfamily A, polypeptide 1
**NM_053453.1**	1.00	-2.23	-1.58	Rgs2	regulator of G-protein signaling 2, 24kDa
**BF282712**	1.00	-2.23	-1.81	Tmem82	transmembrane protein 82
**U75928**	1.00	-2.24	-2.72	Sparc	secreted protein, acidic, cysteine-rich (osteonectin)
**AW918622**	1.00	-2.25	-1.02	Null	EST349926
**AI598486**	1.00	-2.25	-1.24	Dpysl3	dihydropyrimidinase-like 3
**BF284364**	1.00	-2.25	-2.53	Null	EST448955 RAT GENE INDEX, NORMALIZED RAT,
**NM_053857.1**	1.00	-2.25	-1.71	Eif4ebp1	eukaryotic translation initiation factor 4E binding protein 1
**AW916860**	1.00	-2.26	-2.54	Null	EST348073
**BF557889**	1.00	-2.26	-1.68	Znf202	zinc finger protein 202
**U86635**	1.00	-2.27	-1.20	Gstm3	glutathione S-transferase M3 (brain)
**AI406821**	1.00	-2.29	-1.88	Nrsn1	neurensin 1

Table S1: Aging

Accession	Young	Aged Control	Aged Treated	Symbol	Description
**BF408325**	1.00	-2.29	-4.15	C21orf63	chromosome 21 open reading frame 63
**AA892049**	1.00	-2.30	-3.07	Null	EST195852 NORMALIZED RAT KIDNEY, BENTO SOARES
**AI072251**	1.00	-2.30	-2.02	Null	UI-R-C2-MU-H-04-0-UI.S1 UI-R-C2
**AA800476**	1.00	-2.31	-3.03	Mettl7a	methyltransferase like 7A
**AI102097**	1.00	-2.31	-2.30	Null	EST211386 NORMALIZED RAT BRAIN, BENTO SOARES
**AI406939**	1.00	-2.31	-6.50	G0s2	G0/G1switch 2
**AW251335**	1.00	-2.32	-2.42	Spbc24_predicted	SPC24, NDC80 kinetochore complex component, homolog (S. cerevisiae)
**BE112913**	1.00	-2.32	-2.37	Null	UI-R-BJ1-AWA-C-09-0-UI.S1 UI-R-BJ1
**NM_057192.1**	1.00	-2.33	-2.22	Wipf1	WAS/WASL interacting protein family, member 1
**BF414143**	1.00	-2.34	-1.45	Gprc5c	G protein-coupled receptor, family C, group 5, member C
**BE113132**	1.00	-2.34	-2.54	Rapgef5	Rap guanine nucleotide exchange factor (GEF) 5
**AI549393**	1.00	-2.34	-2.84	Null	UI-R-C3-UA-C-05-0-UI.S1 UI-R-C3
**NM_133395.1**	1.00	-2.37	-1.90	Serinc5	serine incorporator 5
**NM_013045.1**	1.00	-2.37	-1.95	Tnr	tenascin R (restrictin, janusin)
**D10354**	1.00	-2.41	-2.47	Gpt	glutamic-pyruvate transaminase (alanine aminotransferase)
**NM_031798.1**	1.00	-2.43	-2.17	Slc12a2	solute carrier family 12 (sodium/potassium/chloride transporters), member 2
**BF405135**	1.00	-2.44	-1.47	Ankrd42_predicted	ankyrin repeat domain 42
**NM_012929.1**	1.00	-2.44	-1.77	Col2a1	collagen, type II, alpha 1
**AI169829**	1.00	-2.44	-5.51	Masp1	mannan-binding lectin serine peptidase 1
**BF544703**	1.00	-2.48	-2.20	Cplx1	complexin 1
**AI170394**	1.00	-2.48	1.07	Null	EST216320 NORMALIZED RAT LUNG, BENTO SOARES
**BF283631**	1.00	-2.49	-1.58	Stk38	serine/threonine kinase 38
**AA817759**	1.00	-2.49	-5.07	Slc25a25	solute carrier family 25 (mitochondrial carrier; phosphate carrier), member 25
**AI411352**	1.00	-2.50	-2.61	Gja1	gap junction protein, alpha 1, 43kDa
**NM_019123.1**	1.00	-2.50	-2.40	St6galnac3	ST6 -N-acetylgalactosaminide alpha-2,6-sialyltransferase 3
**BF290834**	1.00	-2.51	-2.55	Null	EST455425 RAT GENE INDEX, NORMALIZED RAT,
**NM_019157.1**	1.00	-2.54	-1.86	Aqp7	aquaporin 7
**AA892778**	1.00	-2.55	-3.02	Null	EST196581 NORMALIZED RAT KIDNEY, BENTO SOARES
**AI105417**	1.00	-2.56	-4.64	C5orf13	chromosome 5 open reading frame 13
**BF548601**	1.00	-2.60	-2.79	Null	UI-R-A0-AU-C-02-0-UI.R1
**AF020045**	1.00	-2.60	-2.27	Itgae	integrin, alpha E

Table S1: Aging

Accession	Young	Aged Control	Aged Treated	Symbol	Description
**NM_053487.1**	1.00	-2.62	-2.27	Pex11a	peroxisomal biogenesis factor 11A
**BF404935**	1.00	-2.62	-1.13	Null	UI-R-CA1-BIJ-E-03-0-UI.S1 UI-R-CA1
**NM_012959.1**	1.00	-2.62	-3.16	Gfra1	GDNF family receptor alpha 1
**AA858758**	1.00	-2.65	-2.76	C6orf105	chromosome 6 open reading frame 105
**BE118454**	1.00	-2.68	-5.04	C9orf24	chromosome 9 open reading frame 24
**M59967**	1.00	-2.73	-3.12	Bdkrb2	bradykinin receptor B2
**NM_013101.1**	1.00	-2.76	-6.00	Pde4a	phosphodiesterase 4A, cAMP-specific
**AA957618**	1.00	-2.77	-3.88	Null	UI-R-E1-GB-D-03-0-UI.S1 UI-R-E1
**U23407**	1.00	-2.78	-1.97	Crabp2	cellular retinoic acid binding protein 2
**NM_013101.1**	1.00	-2.80	-5.53	Pde4a	phosphodiesterase 4A, cAMP-specific
**BF522695**	1.00	-2.81	-3.03	Sall2	sal-like 2 (Drosophila)
**NM_017084.1**	1.00	-2.82	-4.95	Gnmt	glycine N-methyltransferase
**AA964289**	1.00	-2.83	-1.76	C1orf192	chromosome 1 open reading frame 192
**AI411897**	1.00	-2.89	-2.52	Null	EST240191 NORMALIZED RAT KIDNEY, BENTO SOARES
**NM_053958.1**	1.00	-2.89	-2.21	Ccr3	chemokine (C-C motif) receptor 3
**AW520812**	1.00	-2.93	-2.51	Phlda3	pleckstrin homology-like domain, family A, member 3
**BF397951**	1.00	-2.94	-3.04	Null	UI-R-BS2-BEF-E-09-0-UI.S1 UI-R-BS2
**BF398605**	1.00	-2.96	-2.92	Acpl2	acid phosphatase-like 2
**AW862656**	1.00	-3.04	-4.16	Pex11a	peroxisomal biogenesis factor 11A
**AW533663**	1.00	-3.04	-3.66	Prodh	proline dehydrogenase (oxidase) 1
**BF387347**	1.00	-3.05	-6.78	Null	UI-R-CA1-BBR-D-06-0-UI.S1 UI-R-CA1
**NM_013167.1**	1.00	-3.09	1.02	Ucp3	uncoupling protein 3 (mitochondrial, proton carrier)
**NM_021760.1**	1.00	-3.17	-2.20	Col5a3	collagen, type V, alpha 3
**M24327**	1.00	-3.21	1.19	Mt1f	metallothionein 1F
**BF283056**	1.00	-3.28	-7.93	Retsat	retinol saturase (all-trans-retinol 13,14-reductase)
**BF550748**	1.00	-3.30	-1.44	Dsp	desmoplakin
**AW916833**	1.00	-3.33	-4.25	Retsat	retinol saturase (all-trans-retinol 13,14-reductase)
**AW917133**	1.00	-3.34	-2.92	Null	EST348437
**J04035**	1.00	-3.39	-2.74	Eln	elastin (supravalvular aortic stenosis, Williams-Beuren syndrome)
**AI180010**	1.00	-3.41	-3.67	Abcb10	ATP-binding cassette, sub-family B (MDR/TAP), member 10
**BF553139**	1.00	-3.42	-2.95	Mylk3	myosin light chain kinase 3

Table S1: Aging

Accession	Young	Aged Control	Aged Treated	Symbol	Description
**NM_031543.1**	1.00	-3.42	-4.12	Cyp2e1	cytochrome P450, family 2, subfamily E, polypeptide 1
**Z78279**	1.00	-3.67	-1.90	COLIA1	collagen, type I, alpha 1
**AF325671**	1.00	-3.69	-8.32	Kcnk2	potassium channel, subfamily K, member 2
**AA818947**	1.00	-3.69	1.12	Null	UI-R-A0-AS-D-12-0-UI.S1 UI-R-A0
**AW918040**	1.00	-3.73	-4.63	Null	EST349344
**BE109138**	1.00	-3.82	-4.10	Znf294	zinc finger protein 294
**AI230287**	1.00	-3.90	-3.31	Eraf_predicted	erythroid associated factor
**L27339.1**	1.00	-3.93	-5.56	Npr3	natriuretic peptide receptor C/guanylate cyclase C (atrionatriuretic peptide receptor C)
**BF558459**	1.00	-4.10	-1.92	C11orf74	chromosome 11 open reading frame 74
**BF405177**	1.00	-4.17	-1.97	Tpd52l1	tumor protein D52-like 1
**AI230431**	1.00	-4.89	-4.93	Null	EST227126 NORMALIZED RAT EMBRYO, BENTO SOARES
**M37394**	1.00	-5.07	-1.74	Egfr	epidermal growth factor receptor
**M33648**	1.00	-6.13	-4.09	Hmgcs2	3-hydroxy-3-methylglutaryl-Coenzyme A synthase 2 (mitochondrial)
**BF394261**	1.00	-8.35	-2.50	Kiaa1604	KIAA1604 protein
**BF417400**	1.00	-11.29	-13.07	Crebl1	cAMP responsive element binding protein-like 1
**AW913978**	1.00	-13.23	-1.07	Null	EST293298
