# Supplementary material for: The Extract of Ginkgo biloba EGb 761 Reactivates a Juvenile Profile in the Skeletal Muscle of Sarcopenic Rats by Transcriptional Reprogramming
Source: PLoS One. 2009 Nov 24;4(11):e7998. doi: 10.1371/journal.pone.0007998 (PMC2778626; doi:10.1371/journal.pone.0007998)
Supplement: Table S2 — (2.19 MB PDF) [file pone.0007998.s002.pdf]

Table S2: EGb 761 effect

Accession	Young	Aged Control	Aged Treated	Symbol	Description
**AA800708**	-1.00	1.00	119.50	Null	EST190205 NORMALIZED RAT LUNG,
**AA818954**	-1.32	1.00	25.59	Null	UI-R-A0-AS-E-08-0-UI.S1 UI-R-A0
**AW913978**	13.23	1.00	12.42	Null	EST293298
**AI178361**	3.21	1.00	7.46	Fam20c	family with sequence similarity 20, member C
**NM_017076.1**	-1.20	1.00	4.35	Pvr	poliovirus receptor
**AA818947**	3.69	1.00	4.12	Null	UI-R-A0-AS-D-12-0-UI.S1 UI-R-A0
**AI234830**	3.08	1.00	3.92	Bxdc1_predicted	brix domain containing 1
**M24327**	3.21	1.00	3.81	Mt1f	metallothionein 1F
**NM_017123.1**	-1.10	1.00	3.77	Areg	amphiregulin (schwannoma-derived growth factor)
**AI180454**	1.33	1.00	3.62	Igf2bp2	insulin-like growth factor 2 mRNA binding protein 2
**NM_053370.1**	-11.47	1.00	3.44	Timm8a	translocase of inner mitochondrial membrane 8 homolog A (yeast)
**NM_013167.1**	3.09	1.00	3.15	Ucp3	uncoupling protein 3 (mitochondrial, proton carrier)
**NM_031561.1**	1.61	1.00	3.06	Cd36	CD36 molecule (thrombospondin receptor)
**BF563403**	1.28	1.00	2.98	Rdh5_predicted	retinol dehydrogenase 5 (11-cis/9-cis)
**BE109711**	3.03	1.00	2.86	Lcp1	lymphocyte cytosolic protein 1 (L-plastin)
**AW917212**	1.25	1.00	2.78	Yipf4	Yip1 domain family, member 4
**NM_031813.1**	-1.71	1.00	2.77	Mybph	myosin binding protein H
**AA894092**	2.76	1.00	2.75	Postn_predicted	periostin, osteoblast specific factor
**BF551318**	1.69	1.00	2.72	Herc4	hect domain and RLD 4
**AI170394**	2.48	1.00	2.65	Null	EST216320 NORMALIZED RAT LUNG,
**U53184**	1.05	1.00	2.64	Litaf	lipopolysaccharide-induced TNF factor
**AI599999**	-1.06	1.00	2.63	Null	EST251702 NORMALIZED RAT EMBRYO,
**AA848639**	-1.98	1.00	2.62	Null	EST191399
**AI411981**	-2.32	1.00	2.60	Null	EST240275 NORMALIZED RAT KIDNEY,
**AW914998**	-1.90	1.00	2.57	Null	EST346302
**AF148210**	1.27	1.00	2.56	Rab6a	RAB6A, member RAS oncogene family
**Z49761**	-1.34	1.00	2.53	Hla-dma	major histocompatibility complex, class II, DM alpha
**NM_012488.1**	-1.36	1.00	2.46	A2m	alpha-2-macroglobulin
**NM_053978.1**	1.16	1.00	2.46	Rab28	RAB28, member RAS oncogene family
**NM_012777.1**	1.35	1.00	2.45	Apod	apolipoprotein D
**AW524724**	-2.39	1.00	2.43	Ryr3	ryanodine receptor 3

Table S2: EGb 761 effect

Accession	Young	Aged Control	Aged Treated	Symbol	Description
**NM_031832.1**	-1.04	1.00	2.42	Lgals3	lectin, galactoside-binding, soluble, 3
**NM_080781.1**	-1.21	1.00	2.33	Copb1	coatomer protein complex, subunit beta 1
**AJ238278**	-1.14	1.00	2.31	Xpo1	exportin 1 (CRM1 homolog, yeast)
**BF396180**	1.74	1.00	2.31	Eif3a	eukaryotic translation initiation factor 3, subunit A
**BF550748**	3.30	1.00	2.29	Dsp	desmoplakin
**AW143008**	-1.30	1.00	2.26	Slc16a6	solute carrier family 16, member 6
**NM_053551.1**	-1.28	1.00	2.25	Pdk4	pyruvate dehydrogenase kinase, isozyme 4
**BE111710**	-1.29	1.00	2.21	Punc_predicted	putative neuronal cell adhesion molecule
**AW918622**	2.25	1.00	2.21	Null	EST349926
**NM_053598.1**	-1.26	1.00	2.21	Nudt4	nudix (nucleoside diphosphate linked moiety X)-type motif 4
**NM_017305.1**	-1.12	1.00	2.19	Gclm	glutamate-cysteine ligase, modifier subunit
**AF158186**	2.87	1.00	2.18	Nip7	nuclear import 7 homolog (S. cerevisiae)
**NM_021693.1**	1.02	1.00	2.17	Snf1lk	SNF1-like kinase
**U30789**	1.67	1.00	2.17	Txnip	thioredoxin interacting protein
**NM_013022.1**	1.27	1.00	2.12	Rock2	Rho-associated, coiled-coil containing protein kinase 2
**BF290638**	1.53	1.00	2.12	Null	EST455229 RAT GENE INDEX, NORMALIZED RAT,
**BE099884**	2.11	1.00	2.11	Null	UI-R-BJ1-ATL-C-08-0-UI.S1 UI-R-BJ1
**BE095605**	1.04	1.00	2.07	Filip1	filamin A interacting protein 1
**NM_031544.1**	-1.82	1.00	2.06	Ampd3	adenosine monophosphate deaminase (isoform E)
**AW917132**	2.23	1.00	2.05	c1qtnf3	C1q and tumor necrosis factor related protein 3
**BF281619**	-1.06	1.00	2.05	Null	EST446210 RAT GENE INDEX, NORMALIZED RAT,
**AA874838**	1.50	1.00	2.05	Null	UI-R-E0-CG-G-03-0-UI.S1
**BE104266**	1.46	1.00	2.04	Kntc1_predicted	kinetochore associated 1
**NM_133298.1**	1.21	1.00	2.03	Gpnmb	glycoprotein (transmembrane) nmb
**BF406646**	1.43	1.00	2.03	Fyttd1	forty-two-three domain containing 1
**NM_019371.1**	-1.10	1.00	2.03	egln3	egl nine homolog 3 (C. elegans)
**NM_053338.1**	1.19	1.00	2.03	Rrad	Ras-related associated with diabetes
**AA892824**	1.75	1.00	2.01	Tnc	tenascin C (hexabrachion)
**AW921149**	-1.26	1.00	1.99	C2	complement component 2
**D88250**	1.81	1.00	1.99	C1s	complement component 1, s subcomponent
**AI102517**	-3.22	1.00	1.98	Null	EST211806 NORMALIZED RAT EMBRYO,

Table S2: EGb 761 effect

Accession	Young	Aged Control	Aged Treated	Symbol	Description
**AI170377**	2.07	1.00	1.97	Null	EST216303 NORMALIZED RAT LUNG,
**NM_019620.1**	1.92	1.00	1.97	Zfp386	zinc finger protein 386 (Kruppel-like)
**AI168935**	-1.06	1.00	1.96	Tmem100	transmembrane protein 100
**BE120729**	-1.27	1.00	1.96	Null	UI-R-CA0-BAY-H-04-0-UI.S1 UI-R-CA0
**AA924945**	1.39	1.00	1.95	Sppl2a	signal peptide peptidase-like 2A
**NM_133398.1**	1.50	1.00	1.94	Mtdh	metadherin
**Z78279**	3.67	1.00	1.93	Col1a1	collagen, type I, alpha 1
**BE101480**	1.76	1.00	1.93	Uimc1	ubiquitin interaction motif containing 1
**AI171162**	1.31	1.00	1.91	Evi2a	ecotropic viral integration site 2A
**AI555466**	1.11	1.00	1.89	Null	UI-R-C2P-QQ-D-12-0-UI.S1 UI-R-C2P
**NM_017317.1**	1.13	1.00	1.87	Rab27a	RAB27A, member RAS oncogene family
**AW141869**	2.70	1.00	1.86	Creg_predicted	cellular repressor of E1A-stimulated genes 1
**NM_024486.1**	-1.12	1.00	1.85	Acvr1	activin A receptor, type I
**BF402407**	1.81	1.00	1.84	Rhobtb2	Rho-related BTB domain containing 2
**NM_012604.1**	-1.93	1.00	1.84	Myh3	myosin, heavy chain 3, skeletal muscle, embryonic
**NM_017031.2**	1.94	1.00	1.84	Pde4b	phosphodiesterase 4B, cAMP-specific
**NM_022236.1**	-2.11	1.00	1.83	Pde10a	phosphodiesterase 10A
**NM_022692.1**	-2.12	1.00	1.83	Rab5a	RAB5A, member RAS oncogene family
**BF525257**	1.06	1.00	1.82	Hspb7	heat shock 27kDa protein family, member 7 (cardiovascular)
**AW251942**	-1.36	1.00	1.82	Null	UI-R-BJ0-ADN-G-12-0-UI.S1 UI-R-BJ0
**NM_053718.1**	-2.41	1.00	1.82	Mllt3	myeloid/lymphoid or mixed-lineage leukemia ; translocated to, 3
**AI008409**	-1.07	1.00	1.81	Null	EST202860 NORMALIZED RAT EMBRYO,
**AW918233**	1.43	1.00	1.81	Null	EST349537
**AI235503**	1.55	1.00	1.80	Null	EST232065 NORMALIZED RAT OVARY,
**AA848821**	1.11	1.00	1.80	Null	EST191582 NORMALIZED RAT LUNG,
**BE111795**	1.59	1.00	1.79	Glrx2	glutaredoxin 2
**BF406312**	-1.35	1.00	1.79	Null	UI-R-CA1-BJF-H-21-0-UI.S1 UI-R-CA1
**BF543359**	1.80	1.00	1.79	Zc3h11a	zinc finger CCCH-type containing 11A
**BF555129**	1.83	1.00	1.79	H2afx	H2A histone family, member X
**AW143467**	1.39	1.00	1.78	Null	EST293867
**NM_013177.1**	1.03	1.00	1.78	Got2	glutamic-oxaloacetic transaminase 2, mitochondrial

Table S2: EGb 761 effect

Accession	Young	Aged Control	Aged Treated	Symbol	Description
**BF283305**	1.09	1.00	1.78	Kctd9_predicted	potassium channel tetramerisation domain containing 9
**NM_020089.1**	2.23	1.00	1.78	null	RIKEN cDNA 4930503B20 gene
**X65747**	-1.32	1.00	1.77	Gnat3	guanine nucleotide binding protein, alpha transducing 3
**NM_013195.1**	1.22	1.00	1.77	Il2rb	interleukin 2 receptor, beta
**NM_131907.1**	1.36	1.00	1.77	Atp2c1	ATPase, Ca++ transporting, type 2C, member 1
**BE110633**	1.02	1.00	1.77	Fam49b	family with sequence similarity 49, member B
**BE112415**	1.93	1.00	1.76	Tmem171	transmembrane protein 171
**BE098743**	1.29	1.00	1.76	C2orf30	chromosome 2 open reading frame 30
**U19516**	-2.42	1.00	1.76	Eif2b5	eukaryotic translation initiation factor 2B, subunit 5 epsilon, 82kDa
**BF550568**	1.66	1.00	1.76	Uba5	ubiquitin-like modifier activating enzyme 5
**AI172189**	-1.34	1.00	1.75	Tmem38b	transmembrane protein 38B
**U06230**	1.88	1.00	1.75	Pros	Rattus norvegicus protein S, mRNA, PARTIAL CDS
**AF015953**	4.53	1.00	1.74	Arntl	aryl hydrocarbon receptor nuclear translocator-like
**NM_052981.1**	-1.28	1.00	1.74	Ccnh	cyclin H
**BF404590**	2.45	1.00	1.74	LOC681004	chromosome 21 open reading frame 66
**M83676**	-1.78	1.00	1.74	Rab12	RAB12, member RAS oncogene family
**BF545958**	-1.64	1.00	1.73	Sf3a1	splicing factor 3a, subunit 1, 120kDa
**BE113190**	1.51	1.00	1.73	Tprkb	TP53RK binding protein
**BE116768**	-1.37	1.00	1.73	Rras2	related RAS viral (r-ras) oncogene homolog 2
**NM_031722.1**	-1.78	1.00	1.73	Tmed2	transmembrane emp24 domain trafficking protein 2
**M92340**	-1.58	1.00	1.72	Il6st	interleukin 6 signal transducer (gp130, oncostatin M receptor)
**AI012074**	1.36	1.00	1.72	Parp16	poly (ADP-ribose) polymerase family, member 16
**BG671536**	1.39	1.00	1.72	Btf3l4	basic transcription factor 3-like 4
**NM_021754.1**	1.16	1.00	1.72	Nop5/Nop58	nucleolar protein NOP5/NOP58
**BE109381**	-1.60	1.00	1.72	H6pd	hexose-6-phosphate dehydrogenase (glucose 1-dehydrogenase)
**U41164**	-1.47	1.00	1.72	Znf180	zinc finger protein 180
**NM_057147.1**	1.25	1.00	1.72	Sec22a	SEC22 vesicle trafficking protein homolog A
**BF282483**	1.61	1.00	1.72	Null	EST447074 RAT GENE INDEX, NORMALIZED RAT,
**NM_022543.1**	1.76	1.00	1.72	Ccdc80	coiled-coil domain containing 80
**BF282700**	-1.35	1.00	1.71	TTc9	tetratricopeptide repeat domain 9
**BE120595**	-1.31	1.00	1.71	Mki67ip	MKI67 (FHA domain) interacting nucleolar phosphoprotein

Table S2: EGb 761 effect

Accession	Young	Aged Control	Aged Treated	Symbol	Description
**AA800382**	1.41	1.00	1.71	Null	EST189879
**AI407095**	1.76	1.00	1.70	Qprt	quinolinate phosphoribosyltransferase
**X57523**	-1.04	1.00	1.70	Tap1	transporter 1, ATP-binding cassette, sub-family B (MDR/TAP)
**NM_024369.1**	1.13	1.00	1.69	Fstl1	follistatin-like 1
**NM_057124.1**	1.22	1.00	1.69	P2ry6	pyrimidinergic receptor P2Y, G-protein coupled, 6
**AI231787**	1.24	1.00	1.69	Null	EST228475 NORMALIZED RAT HEART,
**NM_013111.1**	-1.18	1.00	1.69	Slc7a1	solute carrier family 7 (cationic amino acid transporter, y+ system), member 1
**BF398182**	-1.01	1.00	1.68	Null	UI-R-BS2-BEN-D-03-0-UI.S1 UI-R-BS2
**BF549492**	1.25	1.00	1.67	Rae1	RAE1 RNA export 1 homolog (S. pombe)
**BF409831**	1.06	1.00	1.67	Epm2aip1_predicted	EPM2A (laforin) interacting protein 1
**AW920769**	-1.11	1.00	1.67	Tmem188	transmembrane protein 188
**NM_012601.1**	1.93	1.00	1.66	Mpg	N-methylpurine-DNA glycosylase
**NM_031772.1**	-1.53	1.00	1.66	Polr1a	polymerase (RNA) I polypeptide A, 194kDa
**AI180420**	1.67	1.00	1.65	Tubgcp3	tubulin, gamma complex associated protein 3
**L11007**	2.09	1.00	1.65	Cdk4	cyclin-dependent kinase 4
**NM_012561.1**	-2.27	1.00	1.65	Fst	follistatin
**NM_031837.1**	1.16	1.00	1.65	Sept9	septin 9
**AJ301634**	-2.18	1.00	1.65	Atxn10	ataxin 10
**AI231210**	-1.04	1.00	1.65	Polr3b	polymerase (RNA) III (DNA directed) polypeptide B
**BF387255**	1.30	1.00	1.65	Null	UI-R-CA1-BBL-D-04-0-UI.S1 UI-R-CA1
**AI231089**	1.42	1.00	1.65	Lhfpl2	lipoma HMGIC fusion partner-like 2
**AI013474**	-2.09	1.00	1.64	Abhd2_predicted	abhydrolase domain containing 2
**NM_024368.1**	-1.55	1.00	1.64	Frk	fyn-related kinase
**AW141130**	1.76	1.00	1.63	Null	EST291162
**BE111666**	-1.23	1.00	1.63	Null	UI-R-BJ1-AVV-D-08-0-UI.S1 UI-R-BJ1
**BF282409**	-1.32	1.00	1.63	1190003J15RIK	RIKEN cDNA 1190003J15 gene
**AI172196**	-1.22	1.00	1.63	Tceal8	transcription elongation factor A (SII)-like 8
**NM_053352.1**	-1.20	1.00	1.63	Cxcr7	chemokine (C-X-C motif) receptor 7
**X67788**	1.00	1.00	1.63	Ezr	ezrin
**AF205635**	1.94	1.00	1.62	Cdc42	cell division cycle 42 pseudogene
**AW918231**	-1.19	1.00	1.62	Ms12	musashi homolog 2 (Drosophila)

Table S2: EGb 761 effect

Accession	Young	Aged Control	Aged Treated	Symbol	Description
**NM_053598.1**	1.15	1.00	1.62	Nudt4	nudix (nucleoside diphosphate linked moiety X)-type motif 4
**NM_031520.1**	-1.47	1.00	1.62	Myh10	myosin, heavy chain 10, non-muscle
**AW916287**	-1.15	1.00	1.61	Null	EST347591
**AW527606**	-1.08	1.00	1.61	Null	UI-R-BT1-AJZ-B-10-0-UI.S1 UI-R-BT1
**AA800199**	1.41	1.00	1.61	RGD1307129	proline rich 13
**AI598507**	1.33	1.00	1.60	Gpatc1_predicted	G patch domain containing 1
**BF557821**	-1.33	1.00	1.60	Ankrd28	ankyrin repeat domain 28
**AA943094**	-1.01	1.00	1.60	Null	EST198593 NORMALIZED RAT BRAIN,
**AI410203**	1.03	1.00	1.59	C1orf83	chromosome 1 open reading frame 83
**AW916148**	-1.41	1.00	1.59	Phf20	PHD finger protein 20
**AI170679**	-1.10	1.00	1.59	Ugp2	UDP-glucose pyrophosphorylase 2
**AA851327**	1.21	1.00	1.59	Null	EST194095 NORMALIZED RAT PLACENTA,
**AI230596**	1.27	1.00	1.59	Kpna1	karyopherin alpha 1 (importin alpha 5)
**U21871**	1.33	1.00	1.59	Tomm20	translocase of outer mitochondrial membrane 20 homolog (yeast)
**AW915491**	1.87	1.00	1.59	Cep170	centrosomal protein 170kDa
**AW919685**	-1.80	1.00	1.58	Cstf2t	cleavage stimulation factor, 3' pre-RNA, subunit 2, 64kDa, tau variant
**AI235674**	1.08	1.00	1.58	Null	EST232236 NORMALIZED RAT OVARY,
**BE126380**	-2.26	1.00	1.58	Nmt1	N-myristoyltransferase 1
**BF284341**	-1.43	1.00	1.58	Null	EST448932
**AW536019**	1.09	1.00	1.58	Arid1a_predicted	AT rich interactive domain 1A (SWI-like)
**BE109520**	-1.37	1.00	1.58	Fam134b	family with sequence similarity 134, member B
**BF283631**	2.49	1.00	1.57	Stk38	serine/threonine kinase 38
**AW252087**	-1.80	1.00	1.57	Hspb7	heat shock 27kDa protein family, member 7 (cardiovascular)
**NM_031131.1**	-1.35	1.00	1.57	Tgfb2	transforming growth factor, beta 2
**AW918387**	1.65	1.00	1.57	Abca1	ATP-binding cassette, sub-family A (ABC1), member 1
**AI179974**	-1.60	1.00	1.57	Golt1b_predicted	golgi transport 1 homolog B (S. cerevisiae)
**Y16774**	-1.45	1.00	1.57	Slc30a4	solute carrier family 30 (zinc transporter), member 4
**NM_013177.1**	1.25	1.00	1.57	Got2	glutamic-oxaloacetic transaminase 2, mitochondrial
**NM_013135.1**	1.51	1.00	1.57	Rasa1	RAS p21 protein activator (GTPase activating protein) 1
**AI411995**	1.09	1.00	1.56	Null	EST240289 NORMALIZED RAT KIDNEY,
**NM_031333.1**	1.53	1.00	1.56	Cdh2	cadherin 2, type 1, N-cadherin (neuronal)

Table S2: EGb 761 effect

Accession	Young	Aged Control	Aged Treated	Symbol	Description
**NM_013082.1**	1.26	1.00	1.56	Sdc2	syndecan 2
**BG671658**	1.07	1.00	1.56	Psmd5_predicted	proteasome (prosome, macropain) 26S subunit, non-ATPase, 5
**NM_017196.1**	1.50	1.00	1.56	Aif1	allograft inflammatory factor 1
**NM_019165.1**	1.70	1.00	1.55	Il18	interleukin 18 (interferon-gamma-inducing factor)
**BG663138**	-1.23	1.00	1.55	Exoc1	exocyst complex component 1
**AW141353**	1.24	1.00	1.55	Igsf1	immunoglobulin superfamily, member 1
**BE111755**	-1.23	1.00	1.55	Prmt7	protein arginine methyltransferase 7
**BE120513**	1.35	1.00	1.55	Taf13_predicted	TAF13 RNA polymerase II, TATA box binding protein (TBP)-associated factor, 18kDa
**AI146056**	1.86	1.00	1.55	Nxph3	neurexophilin 3
**AW920575**	1.17	1.00	1.55	Null	EST351879
**NM_021586.1**	1.19	1.00	1.55	Ltbp2	latent transforming growth factor beta binding protein 2
**AI409065**	-1.24	1.00	1.55	Vash2	vasohibin 2
**AI409501**	1.18	1.00	1.55	Null	EST237793 NORMALIZED RAT KIDNEY,
**NM_019242.1**	-1.69	1.00	1.53	Ifrd1	interferon-related developmental regulator 1
**AA946434**	1.09	1.00	1.53	Manba	mannosidase, beta A, lysosomal
**AW918470**	-1.28	1.00	1.53	Ankrd28	ankyrin repeat domain 28
**AF016047**	1.72	1.00	1.53	Pafah1b3	platelet-activating factor acetylhydrolase, isoform Ib, gamma subunit 29kDa
**NM_022229.1**	-2.79	1.00	1.52	Hspd1	heat shock 60kDa protein 1 (chaperonin)
**AW529298**	1.03	1.00	1.52	Trafd1	TRAF-type zinc finger domain containing 1
**BF557668**	-1.38	1.00	1.52	Null	UI-R-C0-HZ-G-04-0-UI.R1
**U77880**	-1.94	1.00	1.52	PDE7-1	phosphodiesterase 7A
**AI008390**	-1.60	1.00	1.52	Vash2	vasohibin 2
**AI170799**	1.11	1.00	1.51	Fbxw2_predicted	F-box and WD repeat domain containing 2
**NM_017356.1**	1.43	1.00	1.51	Hpcal1	hippocalcin-like 1
**AI176970**	-1.37	1.00	1.51	Armc1_predicted	armadillo repeat containing 1
**BE098713**	1.43	1.00	1.51	Null	UI-R-BJ1-ATD-A-11-0-UI.S1 UI-R-BJ1
**NM_019370.1**	1.28	1.00	1.51	Enpp3	ectonucleotide pyrophosphatase/phosphodiesterase 3
**NM_017187.1**	1.11	1.00	1.51	Hmgb2	high-mobility group box 2
**NM_032066.1**	-1.41	1.00	1.51	Hsd17b12	hydroxysteroid (17-beta) dehydrogenase 12
**NM_031735.1**	1.32	1.00	1.50	Stk3	serine/threonine kinase 3 (STE20 homolog, yeast)
**AA850347**	-1.04	1.00	1.50	Chp	calcium binding protein P22

Table S2: EGb 761 effect

Accession	Young	Aged Control	Aged Treated	Symbol	Description
**AA893505**	1.20	1.00	1.50	Null	EST197308 NORMALIZED RAT LIVER,
**AF059530**	-1.53	1.00	1.50	Prmt3	protein arginine methyltransferase 3
**NM_080910.1**	1.16	1.00	1.49	Paics	phosphoribosylaminoimidazole succinocarboxamide synthetase
**AW526352**	1.29	1.00	1.49	Null	UI-R-BO1-AJB-B-08-0-UI.S1 UI-R-BO1
**AI410546**	-1.18	1.00	1.49	Loc686999	similar to M phase phosphoprotein 6
**NM_031831.1**	1.09	1.00	1.49	Rtn4	reticulon 4
**NM_021752.1**	-1.59	1.00	1.49	Birc2	baculoviral IAP repeat-containing 2
**AA892294**	1.30	1.00	1.49	Null	EST196097 NORMALIZED RAT KIDNEY,
**AW918103**	1.16	1.00	1.49	Null	EST349407
**AI454928**	-1.21	1.00	1.49	C2orf60	chromosome 2 open reading frame 60
**AF387513**	1.45	1.00	1.49	Bambi	BMP and activin membrane-bound inhibitor homolog
**BE095474**	1.28	1.00	1.48	Null	UI-R-BU0-APA-A-10-0-UI.S1 UI-R-BU0
**U05989**	-1.40	1.00	1.48	Pawr	PRKC, apoptosis, WT1, regulator
**AI102591**	1.01	1.00	1.48	Snrpd1_predicted	small nuclear ribonucleoprotein D1 polypeptide 16kDa
**AW528778**	-1.58	1.00	1.48	Xpc_predicted	xeroderma pigmentosum, complementation group C
**AW523386**	-1.11	1.00	1.48	Pfdn2	prefoldin subunit 2
**NM_013226.1**	1.09	1.00	1.48	Rpl32	ribosomal protein L32
**NM_012659.1**	1.69	1.00	1.48	Sst	somatostatin
**NM_031553.1**	-1.05	1.00	1.47	Nfyb	nuclear transcription factor Y, beta
**NM_017211.1**	-2.39	1.00	1.47	Glg1	golgi apparatus protein 1
**AA892770**	-1.78	1.00	1.47	Gclc	glutamate-cysteine ligase, catalytic subunit
**NM_020072.1**	1.38	1.00	1.47	Acpp	acid phosphatase, prostate
**AW525285**	1.53	1.00	1.47	LOC691862	hypothetical protein LOC691862
**AA892496**	2.01	1.00	1.47	Null	EST196299 NORMALIZED RAT KIDNEY,
**BF414004**	1.05	1.00	1.46	C18orf37	chromosome 18 open reading frame 37
**NM_031020.1**	-1.09	1.00	1.46	Mapk14	mitogen-activated protein kinase 14
**M13979**	-1.02	1.00	1.46	Slc2a1	solute carrier family 2 (facilitated glucose transporter), member 1
**AW915952**	1.35	1.00	1.46	Gins4	GINS complex subunit 4 (Sld5 homolog)
**NM_019149.1**	-1.43	1.00	1.46	Matr3	matrin 3
**BF399614**	-1.40	1.00	1.45	Ovol1	ovo-like 1(Drosophila)
**NM_053404.1**	1.58	1.00	1.45	Dctn4	dynactin 4 (p62)

Table S2: EGb 761 effect

Accession	Young	Aged Control	Aged Treated	Symbol	Description
**AW523875**	1.01	1.00	1.45	C14orf50	chromosome 14 open reading frame 50
**NM_017131.1**	-2.02	1.00	1.45	Casq2	calsequestrin 2 (cardiac muscle)
**BE111820**	1.24	1.00	1.45	Null	UI-R-BJ1-AVZ-B-07-0-UI.S1 UI-R-BJ1
**NM_021760.1**	3.17	1.00	1.44	Col5a3	collagen, type V, alpha 3
**NM_020083.1**	1.27	1.00	1.44	null	
**BE112781**	1.54	1.00	1.44	C10orf78	chromosome 10 open reading frame 78
**BG665133**	1.67	1.00	1.44	Gng2	guanine nucleotide binding protein (G protein), gamma 2
**AI176515**	-1.66	1.00	1.44	Filip1	filamin A interacting protein 1
**AW144670**	-1.38	1.00	1.44	Mak16	MAK16 homolog (S. cerevisiae)
**AI317841**	1.01	1.00	1.43	Gramd3	GRAM domain containing 3
**X00469**	2.22	1.00	1.42	Cyp1a1	cytochrome P450, family 1, subfamily A, polypeptide 1
**AI231433**	-1.16	1.00	1.42	Arih1	ariadne homolog, ubiquitin-conjugating enzyme E2 binding protein, 1
**NM_017282.1**	1.02	1.00	1.42	Psma5	proteasome (prosome, macropain) subunit, alpha type, 5
**BE113224**	1.52	1.00	1.42	Ssr3	signal sequence receptor, gamma
**AI177753**	1.19	1.00	1.42	Null	EST221396 NORMALIZED RAT PLACENTA,
**NM_012511.1**	1.83	1.00	1.42	Atp7b	ATPase, Cu++ transporting, beta polypeptide
**NM_017092.1**	1.88	1.00	1.42	Tyro3	TYRO3 protein tyrosine kinase
**AW915009**	-1.77	1.00	1.42	Null	EST346313
**BE115058**	1.35	1.00	1.42	Null	UI-R-BJ1-AWU-G-05-0-UI.S1 UI-R-BJ1
**BF567496**	1.06	1.00	1.42	Bc066107	cDNA sequence BC066107
**AW916119**	1.12	1.00	1.41	Null	EST347423 RAT GENE INDEX
**AI231808**	1.25	1.00	1.41	Palmd	palmdelphin
**AA818438**	1.29	1.00	1.41	Null	UI-R-A0-AU-H-04-0-UI.S1 UI-R-A0
**BF283001**	-1.15	1.00	1.41	Mrpl27_predicted	mitochondrial ribosomal protein L27
**L18889**	-1.09	1.00	1.41	Canx	calnexin
**AF056034**	-1.12	1.00	1.41	Nexn	nexilin (F actin binding protein)
**BE113966**	1.50	1.00	1.41	Gmfg	glia maturation factor, gamma
**NM_017326.1**	1.10	1.00	1.41	Calm2	calmodulin 2 (phosphorylase kinase, delta)
**BF550779**	1.24	1.00	1.41	Slc9a6_predicted	solute carrier family 9 (sodium/hydrogen exchanger), member 6
**BF405144**	-1.02	1.00	1.40	Fam105b	family with sequence similarity 105, member B
**AA800241**	-1.38	1.00	1.40	Arl6ip5	ADP-ribosylation-like factor 6 interacting protein 5

Table S2: EGb 761 effect

Accession	Young	Aged Control	Aged Treated	Symbol	Description
**AW143820**	1.36	1.00	1.40	LOC691995	hypothetical protein LOC691995
**AW918273**	-1.12	1.00	1.40	Dazap1	DAZ associated protein 1
**AA925353**	-1.01	1.00	1.40	Laptm5	lysosomal associated multispanning membrane protein 5
**AW919336**	1.00	1.00	1.40	Rad23b	RAD23 homolog B (S. cerevisiae)
**AI410438**	-1.54	1.00	1.40	Null	EST238731 NORMALIZED RAT HEART,
**NM_053534.1**	-1.27	1.00	1.39	Gpr175	G protein-coupled receptor 175
**AF012714**	1.11	1.00	1.39	Minpp1	multiple inositol polyphosphate histidine phosphatase, 1
**AI409899**	-1.18	1.00	1.39	Slc20a2	solute carrier family 20 (phosphate transporter), member 2
**BF398053**	1.01	1.00	1.39	Metap1_predicted	methionyl aminopeptidase 1
**AI412591**	1.09	1.00	1.39	RGD1559740_predicted	
**NM_031630.1**	1.20	1.00	1.39	Ddx25	DEAD (Asp-Glu-Ala-Asp) box polypeptide 25
**NM_012653.1**	1.85	1.00	1.39	Slc9a2	solute carrier family 9 (sodium/hydrogen exchanger), member 2
**BF388434**	-1.48	1.00	1.39	Fam49b	family with sequence similarity 49, member B
**NM_080577.1**	-1.12	1.00	1.38	Nploc4	nuclear protein localization 4 homolog (S. cerevisiae)
**NM_053998.1**	-1.09	1.00	1.38	Rab8a	RAB8A, member RAS oncogene family
**NM_012682.1**	1.30	1.00	1.38	Ucp1	uncoupling protein 1 (mitochondrial, proton carrier)
**BF556693**	1.27	1.00	1.38	Null	UI-R-C0-GZ-B-05-0-UI.R1
**NM_031627.1**	-1.19	1.00	1.38	Nr1h3	nuclear receptor subfamily 1, group H, member 3
**AW918076**	1.03	1.00	1.38	Phospho2	phosphatase, orphan 2
**U21719**	1.16	1.00	1.37	Ddx21b	DEAD (Asp-Glu-Ala-Asp) box polypeptide 21b
**AW140640**	1.07	1.00	1.37	C20orf111	chromosome 20 open reading frame 111
**X62660**	1.38	1.00	1.37	Gst4a	glutathione S-transferase A4
**NM_134455.1**	1.01	1.00	1.37	Cxcl1	chemokine (C-X3-C motif) ligand 1
**BF284127**	-2.16	1.00	1.37	Nif3l1	NIF3 NGG1 interacting factor 3-like 1 (S. pombe)
**AJ011608**	-2.49	1.00	1.37	Prim1	primase, DNA, polypeptide 1 (49kDa)
**AJ010392**	-1.03	1.00	1.37	Vsp54	vacuolar protein sorting 54 homolog (S. cerevisiae)
**NM_031579.1**	1.10	1.00	1.37	Ptp4a1	protein tyrosine phosphatase type IVA, member 1
**AW916216**	-1.18	1.00	1.37	Wipi2	WD repeat domain, phosphoinositide interacting 2
**NM_012998.1**	-2.07	1.00	1.36	P4hb	procollagen-proline, 2-oxoglutarate 4-dioxygenase, beta polypeptide
**NM_031978.1**	-1.30	1.00	1.36	Psmd1	proteasome (prosome, macropain) 26S subunit, non-ATPase, 1
**AA818120**	-1.56	1.00	1.36	Sln	sarcolipin

Table S2: EGb 761 effect

Accession	Young	Aged Control	Aged Treated	Symbol	Description
**AA944549**	-1.35	1.00	1.36	Loc90379	hypothetical protein BC002926
**BF407878**	1.22	1.00	1.36	Sap18	Sin3A-associated protein, 18kDa
**NM_013120.1**	1.24	1.00	1.36	Gckr	glucokinase (hexokinase 4) regulator
**BE116582**	1.13	1.00	1.35	Null	UI-R-BS1-AYJ-H-08-0-UI.S1 UI-R-BS1
**BF550554**	-1.60	1.00	1.34	Null	UI-R-C0-JJ-F-05-0-UI.R1
**BE108745**	-1.08	1.00	1.34	Nsbp1	nucleosomal binding protein 1
**AI011757**	1.11	1.00	1.34	Fcgr3a	Fc fragment of IgG, low affinity IIIa, receptor (CD16a)
**NM_012773.1**	1.04	1.00	1.34	Akap11	A kinase (PRKA) anchor protein 11
**AI230697**	-1.22	1.00	1.33	Ssr1	signal sequence receptor, alpha
**AI170390**	1.06	1.00	1.33	Null	EST216316 NORMALIZED RAT LUNG,
**NM_031562.1**	2.07	1.00	1.33	Csn3	casein kappa
**X83579**	-1.19	1.00	1.32	Cdk7	cyclin-dependent kinase 7
**AW918358**	1.50	1.00	1.31	C123orf1	chromosome 13 open reading frame 1
**L07578**	-1.12	1.00	1.31	Ck1d	Rat casein kinase 1 delta mRNA
**AA963282**	1.10	1.00	1.26	Nmnat3	nicotinamide nucleotide adenylyltransferase 3
**AW919920**	1.15	1.00	-1.30	Hspa12b_predicted	heat shock 70kD protein 12B
**BE110614**	1.23	1.00	-1.32	Null	UI-R-BJ1-AVU-A-07-0-UI.S1 UI-R-BJ1
**BE118972**	1.21	1.00	-1.32	Null	UI-R-BS1-AZW-H-11-0-UI.S1 UI-R-BS1
**AA851256**	1.07	1.00	-1.32	Null	EST194024 NORMALIZED RAT PLACENTA,
**NM_031058.1**	1.01	1.00	-1.33	Msh2	mutS homolog 2, colon cancer, nonpolyposis type 1
**AA866426**	1.18	1.00	-1.33	Null	UI-R-E0-CH-D-05-0-UI.S1 UI-R-E0
**NM_013192.1**	-1.07	1.00	-1.34	Kcnj6	potassium inwardly-rectifying channel, subfamily J, member 6
**AW525211**	-1.01	1.00	-1.34	Pycr2	pyrroline-5-carboxylate reductase family, member 2
**AI407903**	-1.02	1.00	-1.35	Cdc42ep4_predicted	CDC42 effector protein (Rho GTPase binding) 4
**AI137756**	1.19	1.00	-1.35	Hpcal1	hippocalcin-like 1
**NM_031348.1**	1.13	1.00	-1.35	Fcn2	ficolin (collagen/fibrinogen domain containing lectin) 2 (hucolin)
**AI716436**	-1.05	1.00	-1.35	Pdss2	prenyl (decaprenyl) diphosphate synthase, subunit 2
**BE101165**	1.08	1.00	-1.35	Tmub1	transmembrane and ubiquitin-like domain containing 1
**NM_012492.1**	1.26	1.00	-1.35	Adrb2	adrenergic, beta-2-, receptor, surface
**BF396191**	1.38	1.00	-1.35	Null	UI-R-BS2-BDV-G-08-0-UI.S1 UI-R-BS2
**BF404589**	1.60	1.00	-1.35	Loc679272	similar to membrane-associated RING-CH protein IX

Table S2: EGb 761 effect

Accession	Young	Aged Control	Aged Treated	Symbol	Description
**AI058901**	1.27	1.00	-1.36	Ccl27_predicted	chemokine (C-C motif) ligand 27
**BF396371**	-1.37	1.00	-1.36	Null	UI-R-BS2-BDX-D-03-0-UI.S1 UI-R-BS2
**NM_133526.1**	1.09	1.00	-1.36	Tspan8	tetraspanin 8
**BF283743**	1.32	1.00	-1.36	Tie1	tyrosine kinase with immunoglobulin-like and EGF-like domains 1
**AI236776**	1.20	1.00	-1.36	Null	EST233338 NORMALIZED RAT OVARY,
**BF392443**	1.50	1.00	-1.37	Null	UI-R-CA0-BFJ-C-07-0-UI.S1 UI-R-CA0
**BF413204**	1.03	1.00	-1.38	Fam73b	family with sequence similarity 73, member B
**AA849497**	-1.03	1.00	-1.38	Acss2_predicted	acyl-CoA synthetase short-chain family member 2
**AW918637**	1.36	1.00	-1.38	Null	EST349941 RAT GENE INDEX
**AI233818**	1.52	1.00	-1.38	Akap12	A kinase (PRKA) anchor protein (gravin) 12
**AW917015**	-1.16	1.00	-1.38	Tbc1d9b	TBC1 domain family, member 9B (with GRAM domain)
**NM_021676.1**	-1.06	1.00	-1.38	Shank3	SH3 and multiple ankyrin repeat domains 3
**NM_032083.1**	1.17	1.00	-1.38	Chn1	chimerin (chimaerin) 1
**AI044124**	1.30	1.00	-1.38	Plekhg5	pleckstrin homology domain containing, family G (with RhoGef domain) member 5
**D00680**	1.31	1.00	-1.38	Gpx3	glutathione peroxidase 3 (plasma)
**AA859010**	-1.11	1.00	-1.39	Null	UI-R-A0-BI-D-10-0-UI.S1 UI-R-A0
**BF551593**	1.19	1.00	-1.39	Null	UI-R-C0-IV-C-01-0-UI.R1 UI-R-C0
**BF394166**	1.16	1.00	-1.39	Gpm6a	glycoprotein M6A
**NM_021660.1**	-1.08	1.00	-1.39	Ihpk2	inositol hexaphosphate kinase 2
**AW528057**	1.64	1.00	-1.39	Null	UI-R-BT1-AKH-B-11-0-UI.S1 UI-R-BT1
**AI177383**	1.74	1.00	-1.39	C6orf145	chromosome 6 open reading frame 145
**NM_012734.1**	-1.13	1.00	-1.39	Hk1	hexokinase 1
**NM_017272.14**	-1.03	1.00	-1.39	Aldh1a7	aldehyde dehydrogenase family 1, subfamily A7
**BF413334**	-1.02	1.00	-1.40	Mdp-1	magnesium-dependent phosphatase 1
**AW914928**	1.32	1.00	-1.40	Hysl1	hydrolethalus syndrome 1
**AI013475**	1.37	1.00	-1.40	Sort1	sortilin 1
**AW915558**	1.08	1.00	-1.40	Tchp_predicted	trichoplein, keratin filament binding
**AI229821**	-1.23	1.00	-1.40	Null	EST226516 NORMALIZED RAT EMBRYO,
**AW920609**	-1.25	1.00	-1.41	Apln	apelin
**BE103793**	-1.25	1.00	-1.41	Kctd15	potassium channel tetramerisation domain containing 15
**AI179472**	-1.12	1.00	-1.41	Null	EST223186 NORMALIZED RAT SPLEEN,

Table S2: EGb 761 effect

Accession	Young	Aged Control	Aged Treated	Symbol	Description
**AI170948**	-1.06	1.00	-1.41	Gpr4	G protein-coupled receptor 4
**AA956764**	1.05	1.00	-1.41	Rhbdf1	rhomboid 5 homolog 1 (Drosophila)
**BF396179**	1.60	1.00	-1.41	Ttyh3_predicted	tweety homolog 3 (Drosophila)
**BE098266**	1.16	1.00	-1.41	Null	
**NM_013180.1**	1.20	1.00	-1.41	Itgb4	integrin, beta 4
**AI177621**	1.36	1.00	-1.41	Icam2	intercellular adhesion molecule 2
**AW143189**	1.26	1.00	-1.41	Rasgrp3	RAS guanyl releasing protein 3 (calcium and DAG-regulated)
**BE101448**	-1.02	1.00	-1.42	Null	
**BE102505**	-1.03	1.00	-1.42	Null	UI-R-BT1-AQN-D-10-0-UI.S1 UI-R-BT1
**BF285568**	1.93	1.00	-1.42	C1orf2	chromosome 1 open reading frame 2
**NM_013092.1**	-1.78	1.00	-1.42	Cma1	chymase 1, mast cell
**AA799450**	1.08	1.00	-1.42	Null	EST188947 NORMALIZED RAT HEART,
**U70050**	-1.68	1.00	-1.42	Jag2	jagged 2
**BF420183**	1.10	1.00	-1.42	Null	UI-R-BJ2-BPJ-H-09-0-UI.S1 UI-R-BJ2
**BG667965**	-1.16	1.00	-1.43	Arl8a	ADP-ribosylation factor-like 8A
**AI599125**	-1.18	1.00	-1.43	Null	EST250828 NORMALIZED RAT EMBRYO,
**NM_012634.1**	-1.02	1.00	-1.43	Prps2	phosphoribosyl pyrophosphate synthetase 2
**AW535349**	1.29	1.00	-1.43	Centb5_predicted	centaurin, beta 5
**NM_019205.1**	-2.34	1.00	-1.44	Ccl11	chemokine (C-C motif) ligand 11
**BF392911**	-1.57	1.00	-1.44	Wnt5b	wingless-type MMTV integration site family, member 5B
**BF420043**	-1.18	1.00	-1.44	Null	UI-R-BJ2-BPM-C-09-0-UI.S1 UI-R-BJ2
**BE100607**	1.12	1.00	-1.44	Pitpnm1	phosphatidylinositol transfer protein, membrane-associated 1
**AI406506**	-1.15	1.00	-1.44	Null	EST234792 NORMALIZED RAT BRAIN,
**NM_031097.1**	1.09	1.00	-1.44	Rnpep	arginyl aminopeptidase (aminopeptidase B)
**NM_019156.1**	1.11	1.00	-1.44	Vtn	vitronectin
**AI013750**	-1.44	1.00	-1.45	Slc39a7	solute carrier family 39 (zinc transporter), member 7
**BF549525**	-1.05	1.00	-1.45	Abra	actin-binding Rho activating protein
**BF387477**	1.04	1.00	-1.45	Gpr116	G protein-coupled receptor 116
**NM_031557.1**	1.08	1.00	-1.46	Ptgis	prostaglandin I2 (prostacyclin) synthase
**NM_031646.1**	1.15	1.00	-1.46	Ramp2	receptor (G protein-coupled) activity modifying protein 2
**AJ223083.1**	-1.71	1.00	-1.46	Rxrg	retinoid X receptor, gamma

Table S2: EGb 761 effect

Accession	Young	Aged Control	Aged Treated	Symbol	Description
**BE109513**	-1.41	1.00	-1.46	Znf703	zinc finger protein 703
**AI102745**	1.13	1.00	-1.46	Kiaa1949	KIAA1949
**AI177140**	1.25	1.00	-1.46	Npy1r	neuropeptide Y receptor Y1
**NM_032616.1**	-1.71	1.00	-1.46	Lsr	lipolysis stimulated lipoprotein receptor
**AI716491**	1.29	1.00	-1.47	Yipf1	Yip1 domain family, member 1
**AA859343**	-1.13	1.00	-1.47	Srpk1	SFRS protein kinase 1
**AI231193**	-1.05	1.00	-1.47	Null	EST227881 NORMALIZED RAT EMBRYO,
**AJ225626**	1.09	1.00	-1.47	Null	RAT LIVER ESTS (E.OLIVIER)
**BE102814**	1.37	1.00	-1.47	Null	UI-R-BT1-AQT-E-11-0-UI.S1 UI-R-BT1
**AI717140**	-2.82	1.00	-1.47	Tmem179	transmembrane protein 179
**BF404344**	1.14	1.00	-1.48	Dkfzp434B0335	DKFZP434B0335 protein
**NM_053336.1**	1.16	1.00	-1.48	Ager	advanced glycosylation end product-specific receptor
**AI227912**	-1.39	1.00	-1.49	Null	EST224607 NORMALIZED RAT BRAIN,
**AW253010**	1.07	1.00	-1.49	Null	UI-R-BJ0-AEQ-C-12-0-UI.S1 UI-R-BJ0
**BF405725**	1.11	1.00	-1.49	Mrgprf	MAS-related GPR, member F
**NM_138535.1**	2.14	1.00	-1.49	Grip2	glutamate receptor interacting protein 2
**AW914770**	1.91	1.00	-1.49	Null	EST346074
**BE108810**	1.20	1.00	-1.49	Notch1	Notch homolog 1, translocation-associated (Drosophila)
**AI030179**	-1.45	1.00	-1.50	Atp6v0e2	ATPase, H+ transporting V0 subunit e2
**AW523746**	1.13	1.00	-1.50	C2orf40	chromosome 2 open reading frame 40
**AI172271**	1.80	1.00	-1.50	Emcn	endomucin
**BF420074**	-1.52	1.00	-1.50	Null	UI-R-BJ2-BPM-F-05-0-UI.S1 UI-R-BJ2
**AI059108**	1.03	1.00	-1.50	Null	UI-R-C1-LR-F-03-0-UI.S1 UI-R-C1
**AW914913**	-2.44	1.00	-1.51	Null	EST346217
**AW915955**	1.01	1.00	-1.51	Fmnl1_predicted	formin-like 1
**NM_030844.1**	1.05	1.00	-1.51	Ica1	islet cell autoantigen 1, 69kDa
**NM_130433.1**	1.58	1.00	-1.51	Acaa2	acetyl-Coenzyme A acyltransferase 2
**AI227996**	1.27	1.00	-1.51	Null	EST224691 NORMALIZED RAT BRAIN,
**NM_054008.1**	1.40	1.00	-1.51	Rgc32	RGC32 protein (RGC32), MRNA
**AI012611**	-1.29	1.00	-1.52	Rasgrp2_predicted	RAS guanyl releasing protein 2 (calcium and DAG-regulated)
**BE109617**	-1.25	1.00	-1.52	Rabl4_predicted	RAB, member of RAS oncogene family-like 4

Table S2: EGb 761 effect

Accession	Young	Aged Control	Aged Treated	Symbol	Description
**AI009796**	1.39	1.00	-1.52	Uckl1	uridine-cytidine kinase 1-like 1
**AW434978**	1.42	1.00	-1.52	Null	UI-R-BJ0P-AFV-B-12-0-UI.S1 UI-R-BJ0P
**AA799656**	1.07	1.00	-1.52	Mrps31_predicted	mitochondrial ribosomal protein S31
**AI102248**	-1.21	1.00	-1.53	Plxnd1_predicted	plexin D1
**AA799981**	1.09	1.00	-1.53	Prkch	protein kinase C, eta
**AW920557**	-1.03	1.00	-1.54	Null	EST351861
**AI170668**	-1.25	1.00	-1.54	Kiaa1191	KIAA1191
**AW142058**	1.30	1.00	-1.55	Fmo2	flavin containing monooxygenase 2 (non-functional)
**Y15054**	-1.45	1.00	-1.55	Coro7	coronin 7
**NM_012519.1**	1.08	1.00	-1.55	Camk2d	calcium/calmodulin-dependent protein kinase (CaM kinase) II delta
**BF407134**	-1.50	1.00	-1.56	Sympk	symplekin
**AW253265**	1.08	1.00	-1.56	Etv6	ets variant gene 6 (TEL oncogene)
**AA818197**	1.25	1.00	-1.56	LOC498331	protein tyrosine phosphatase, non-receptor type 13
**AA946128**	1.14	1.00	-1.56	Null	EST201627 NORMALIZED RAT LUNG,
**AI406271**	1.06	1.00	-1.56	Null	EST234557 NORMALIZED RAT BRAIN,
**NM_012676.1**	-5.49	1.00	-1.57	Tnnt2	troponin T type 2 (cardiac)
**AW918039**	1.50	1.00	-1.57	C16orf7	chromosome 16 open reading frame 7
**AA955175**	1.92	1.00	-1.57	Null	UI-R-A1-DU-B-04-0-UI.S1 UI-R-A1
**J03959**	1.33	1.00	-1.57	Uox	urate oxidase (pseudogene)
**AA899865**	-1.47	1.00	-1.57	Alg2	asparagine-linked glycosylation 2 homolog
**BE104254**	1.22	1.00	-1.57	Ppp1r12c	protein phosphatase 1, regulatory (inhibitor) subunit 12C
**AA997412**	1.29	1.00	-1.57	Null	UI-R-C0-HR-E-10-0-UI.S1 UI-R-C0
**NM_017139.1**	-4.31	1.00	-1.58	Penk	proenkephalin
**AW915595**	-3.98	1.00	-1.58	Adipoq	adiponectin, C1Q and collagen domain containing
**BE117002**	-1.20	1.00	-1.58	Gpsm2	G-protein signaling modulator 2 (AGS3-like, C. elegans)
**NM_053633.1**	-1.10	1.00	-1.58	Egr2	early growth response 2 (Krox-20 homolog, Drosophila)
**AW915996**	1.57	1.00	-1.58	Adamtsl2	ADAMTS-like 2
**AA893584**	1.15	1.00	-1.58	Bloc1s2	biogenesis of lysosome-related organelles complex-1, subunit 2
**AA892522**	1.05	1.00	-1.58	Slc25a30	solute carrier family 25, member 30
**BF555099**	1.11	1.00	-1.58	Ttll5	tubulin tyrosine ligase-like family, member 5
**AA998047**	1.16	1.00	-1.59	Null	UI-R-C0-HX-H-07-0-UI.S1 UI-R-C0

Table S2: EGb 761 effect

Accession	Young	Aged Control	Aged Treated	Symbol	Description
**AI412393**	1.15	1.00	-1.60	Hspa12a_predicted	heat shock 70kDa protein 12A
**AI104125**	-1.83	1.00	-1.60	Mrps27_predicted	mitochondrial ribosomal protein S27
**AA946222**	1.04	1.00	-1.61	Bcar3_predicted	breast cancer anti-estrogen resistance 3
**AA819832**	1.34	1.00	-1.61	Per1	period homolog 1
**BF551250**	-2.09	1.00	-1.61	Fkbp5	FK506 binding protein 5
**BF285185**	-1.42	1.00	-1.62	Null	EST449776 RAT GENE INDEX, NORMALIZED RAT,
**NM_053605.1**	1.79	1.00	-1.62	Smpd3	sphingomyelin phosphodiesterase 3, neutral membrane
**NM_134349.1**	-1.72	1.00	-1.62	Mgst1	microsomal glutathione S-transferase 1
**BE107674**	1.17	1.00	-1.62	Null	UI-R-BT1-AME-E-08-0-UI.S1 UI-R-BT1
**BF522317**	1.26	1.00	-1.62	Null	UI-R-C2P-QN-H-10-0-UI.R1
**AI406984**	1.99	1.00	-1.62	Itga8	integrin, alpha 8
**AW917484**	-1.09	1.00	-1.62	Null	EST348788
**U44948**	-1.05	1.00	-1.62	Csrp2	cysteine and glycine-rich protein 2
**AA848826**	-1.07	1.00	-1.63	Asah3l	N-acylsphingosine amidohydrolase 3-like
**AW142280**	-1.03	1.00	-1.63	Ltbp4	latent transforming growth factor beta binding protein 4
**NM_031747.1**	1.00	1.00	-1.63	Cnn1	calponin 1, basic, smooth muscle
**NM_019285.1**	1.16	1.00	-1.63	Adcy4	adenylate cyclase 4
**D25290**	1.01	1.00	-1.64	Cdh6	cadherin 6, type 2, K-cadherin (fetal kidney)
**NM_031819.1**	-2.35	1.00	-1.64	Fat	FAT tumor suppressor homolog 1 (Drosophila)
**AA945750**	1.66	1.00	-1.64	Null	EST201249 NORMALIZED RAT LUNG,
**AF139830**	-1.34	1.00	-1.64	Igfbp5	insulin-like growth factor binding protein 5
**NM_130829.1**	-1.02	1.00	-1.64	Palm	paralemmin
**AI180252**	1.07	1.00	-1.65	Null	EST223995 NORMALIZED RAT SPLEEN,
**BI282127**	1.17	1.00	-1.65	Hint2_predicted	histidine triad nucleotide binding protein 2
**AW144233**	1.34	1.00	-1.65	St6galnac2	ST6 -N-acetylgalactosaminide alpha-2,6-sialyltransferase 2
**AI105366**	-1.19	1.00	-1.65	C10orf33	chromosome 10 open reading frame 33
**NM_013027.1**	-1.19	1.00	-1.66	Sepw1	selenoprotein W, 1
**AI176680**	1.49	1.00	-1.66	Rbpms2	RNA binding protein with multiple splicing 2
**AI409024**	1.03	1.00	-1.66	Plac8_predicted	placenta-specific 8
**NM_133562.1**	1.16	1.00	-1.66	Pib5pa	phosphatidylinositol (4,5) bisphosphate 5-phosphatase, A
**AI234142**	-1.01	1.00	-1.67	Iqcb1_predicted	IQ motif containing B1

Table S2: EGb 761 effect

Accession	Young	Aged Control	Aged Treated	Symbol	Description
**BE100748**	-5.29	1.00	-1.67	C13orf30	chromosome 13 open reading frame 30
**BF398114**	-1.21	1.00	-1.68	Null	UI-R-BS2-BEM-E-10-0-UI.S1 UI-R-BS2
**BF410042**	1.15	1.00	-1.68	Null	UI-R-CA0-BJS-H-01-0-UI.S1 UI-R-CA0
**M90661**	-1.13	1.00	-1.68	Insrr	insulin receptor-related receptor
**BE109120**	1.46	1.00	-1.68	Dzip1	DAZ interacting protein 1
**BE097102**	-1.57	1.00	-1.68	Null	
**BE116867**	-1.19	1.00	-1.68	Null	UI-R-BS1-AZG-B-09-0-UI.S1 UI-R-BS1
**AF016387**	-1.07	1.00	-1.68	Rxrg	retinoid X receptor, gamma
**AW527473**	1.09	1.00	-1.68	Null	UI-R-BO1-AJT-G-02-0-UI.S1 UI-R-BO1
**NM_017136.1**	1.01	1.00	-1.69	Sqle	squalene epoxidase
**AA943573**	1.11	1.00	-1.69	Gla	galactosidase, alpha
**AW141446**	1.36	1.00	-1.70	Nudt14_predicted	nudix (nucleoside diphosphate linked moiety X)-type motif 14
**BF397956**	-1.14	1.00	-1.70	Null	UI-R-BS2-BEF-F-02-0-UI.S1 UI-R-BS2
**NM_134387.1**	1.87	1.00	-1.70	Dcxr	dicarbonyl/L-xylulose reductase
**BF404472**	1.64	1.00	-1.71	Sh2d3c_predicted	SH2 domain containing 3C
**AI411212**	-1.24	1.00	-1.71	Pdlim2	PDZ and LIM domain 2 (mystique)
**AI228222**	-1.16	1.00	-1.71	Null	EST224917 NORMALIZED RAT BRAIN,
**AF189709**	1.33	1.00	-1.73	Col18a1	collagen, type XVIII, alpha 1
**AI176713**	1.59	1.00	-1.73	Dlc1	deleted in liver cancer 1
**BE111696**	-1.31	1.00	-1.73	Fam36a	family with sequence similarity 36, member A
**AI411141**	1.15	1.00	-1.73	Null	EST239435 NORMALIZED RAT KIDNEY,
**AF000942.1**	1.16	1.00	-1.73	Id3	inhibitor of DNA binding 3, dominant negative helix-loop-helix protein
**NM_012585.1**	-1.26	1.00	-1.73	Htr1a	5-hydroxytryptamine (serotonin) receptor 1A
**AB025017**	1.16	1.00	-1.73	Zfp36	zinc finger protein 36, C3H type, homolog (mouse)
**BE100823**	-1.15	1.00	-1.74	Zdhhc4	zinc finger, DHHC-type containing 4
**NM_024364.1**	-1.12	1.00	-1.75	Hr	hairless homolog (mouse)
**AW526033**	1.12	1.00	-1.75	Null	UI-R-BO1-AIY-E-04-0-UI.S1 UI-R-BO1
**BF414998**	-1.16	1.00	-1.75	Flj12993	hypothetical LOC441027
**AW253408**	-1.12	1.00	-1.75	Amigo2	adhesion molecule with Ig-like domain 2
**NM_017084.1**	2.82	1.00	-1.75	Gnmt	glycine N-methyltransferase
**NM_012731.1**	-1.08	1.00	-1.76	Ntrk2	neurotrophic tyrosine kinase, receptor, type 2

Table S2: EGb 761 effect

Accession	Young	Aged Control	Aged Treated	Symbol	Description
**AI111863**	1.36	1.00	-1.76	Null	UI-R-Y0-MP-F-02-0-UI.S1 UI-R-Y0
**NM_019350.1**	-1.07	1.00	-1.77	Syt5	synaptotagmin V
**AI411222**	1.40	1.00	-1.77	Timp4	TIMP metallopeptidase inhibitor 4
**AW917540**	1.42	1.00	-1.77	Cmtm8	CKLF-like MARVEL transmembrane domain containing 8
**AW144517**	1.16	1.00	-1.78	Null	EST294894 NORMALIZED RAT EMBRYO,
**NM_057207.2**	1.23	1.00	-1.78	Sv2b	synaptic vesicle glycoprotein 2B
**AA945604**	-1.20	1.00	-1.79	Null	EST201103 NORMALIZED RAT LIVER,
**AA944556**	1.14	1.00	-1.79	Nope	neighbor of Punc E11
**NM_031658.1**	1.38	1.00	-1.79	Msln	mesothelin
**NM_133525.1**	-1.71	1.00	-1.81	C6orf108	chromosome 6 open reading frame 108
**BF291161**	1.10	1.00	-1.81	Null	EST455752 RAT GENE INDEX, NORMALIZED RAT,
**AI105417**	2.56	1.00	-1.81	C5orf13	chromosome 5 open reading frame 13
**BE109637**	1.83	1.00	-1.81	Fnsk_predicted	fructosamine 3 kinase
**AI410818**	2.09	1.00	-1.81	Null	EST239111 NORMALIZED RAT HEART,
**NM_032071.1**	-1.68	1.00	-1.82	Sunj2	synaptojanin 2
**AI101322**	-1.04	1.00	-1.83	Hdac11_predicted	histone deacetylase 11
**NM_013197.1**	1.61	1.00	-1.83	Alas2	aminolevulinate, delta-, synthase 2
**AW254246**	1.15	1.00	-1.84	Null	UI-R-BJ0-AEV-D-04-0-UI.S1 UI-R-BJ0
**NM_031549.1**	-1.61	1.00	-1.85	Tagln	transgelin
**BF412664**	1.91	1.00	-1.85	Null	UI-R-BT1-BNQ-F-02-0-UI.S1 UI-R-BT1
**NM_017351.1**	-1.24	1.00	-1.86	Itih3	inter-alpha (globulin) inhibitor H3
**BF389910**	-1.26	1.00	-1.86	Rab3a	RAB3A, member RAS oncogene family
**NM_013058.1**	1.06	1.00	-1.87	Id3	inhibitor of DNA binding 3, dominant negative helix-loop-helix protein
**BE118454**	2.68	1.00	-1.88	C9orf24	chromosome 9 open reading frame 24
**AI232784**	-1.77	1.00	-1.89	Bdh2_predicted	3-hydroxybutyrate dehydrogenase, type 2
**BG664142**	1.02	1.00	-1.89	C1orf71	chromosome 1 open reading frame 71
**BF283898**	2.20	1.00	-1.89	Klhl5	kelch-like 5 (Drosophila)
**AW435010**	1.10	1.00	-1.89	Ptpn3	protein tyrosine phosphatase, non-receptor type 3
**AI406660**	1.50	1.00	-1.90	Thbs1	
**NM_012884.1**	-1.12	1.00	-1.92	Cntn2	contactin 2 (axonal)
**NM_080892.1**	-1.10	1.00	-1.92	Selenbp1	selenium binding protein 1

Table S2: EGb 761 effect

Accession	Young	Aged Control	Aged Treated	Symbol	Description
**NM_057190.1**	-2.01	1.00	-1.92	Nelf	nasal embryonic LHRH factor
**AW916151**	-1.02	1.00	-1.92	Xkr8	XK, Kell blood group complex subunit-related family, member 8
**NM_017332.1**	-1.76	1.00	-1.93	Fasn	fatty acid synthase
**NM_080688.1**	1.44	1.00	-1.93	Plcd4	phospholipase C, delta 4
**AI101924**	-1.00	1.00	-1.94	Heyl	hairy/enhancer-of-split related with YRPW motif-like
**AW143336**	1.00	1.00	-1.94	Null	EST293632 NORMALIZED RAT BRAIN,
**AI406809**	-1.50	1.00	-1.95	Null	EST235096 NORMALIZED RAT BRAIN,
**AW915585**	-1.42	1.00	-1.96	Cdc42ep1_predicted	CDC42 effector protein (Rho GTPase binding) 1
**AI137286**	-1.30	1.00	-1.96	Null	UI-R-C2P-OL-A-06-0-UI.S1 UI-R-C2P
**AI171607**	1.76	1.00	-1.96	Fam10a7	family with sequence similarity 107, member A
**NM_013101.1**	2.80	1.00	-1.97	Pde4a	phosphodiesterase 4A, cAMP-specific
**AI170387**	1.02	1.00	-1.98	Cxcl9	chemokine (C-X-C motif) ligand 9
**X95189**	-1.31	1.00	-2.00	Acox2	acyl-Coenzyme A oxidase 2, branched chain
**BF284693**	1.17	1.00	-2.01	Tob2	transducer of ERBB2, 2
**AW520354**	-1.24	1.00	-2.02	Prr12	proline rich 12
**NM_013190.1**	-1.12	1.00	-2.02	Pfkl	phosphofructokinase, liver
**AW918255**	1.79	1.00	-2.03	Dgat2	diacylglycerol O-acyltransferase homolog 2 (mouse)
**AA817759**	2.49	1.00	-2.04	Slc25a25	solute carrier family 25 (mitochondrial carrier; phosphate carrier), member 25
**AI716642**	-1.36	1.00	-2.05	Lynx1_predicted	Ly6/neurotoxin 1
**AI145761**	1.15	1.00	-2.05	Null	UI-R-BT0-PY-D-07-0-UI.S1 UI-R-BT0
**NM_012797.1**	1.58	1.00	-2.08	Id1	inhibitor of DNA binding 1, dominant negative helix-loop-helix protein
**AF024622**	-1.40	1.00	-2.10	Gucy2g	guanylate cyclase 2g
**NM_013101.1**	2.76	1.00	-2.17	Pde4a	phosphodiesterase 4A, cAMP-specific
**AI230056**	-1.11	1.00	-2.20	Null	EST226751 NORMALIZED RAT EMBRYO,
**BF387347**	3.05	1.00	-2.22	Null	UI-R-CA1-BBR-D-06-0-UI.S1 UI-R-CA1
**BE106816**	-1.07	1.00	-2.23	Null	UI-R-BT1-AST-E-05-0-UI.S1 UI-R-BT1
**AW251849**	2.93	1.00	-2.23	Dscc1	defective in sister chromatid cohesion 1 homolog (S. cerevisiae)
**BF408444**	1.44	1.00	-2.24	Null	UI-R-BJ2-BRC-G-11-0-UI.S1 UI-R-BJ2
**AI169829**	2.44	1.00	-2.26	Masp1	mannan-binding lectin serine peptidase 1
**AF325671**	3.69	1.00	-2.26	Kcnk2	potassium channel, subfamily K, member 2
**NM_012621.1**	1.49	1.00	-2.27	Pfkfb1	6-phosphofructo-2-kinase/fructose-2,6-biphosphatase 1

Table S2: EGb 761 effect

Accession	Young	Aged Control	Aged Treated	Symbol	Description
**AW531805**	-1.72	1.00	-2.27	Ifit3	interferon-induced protein with tetratricopeptide repeats 3
**NM_053608.1**	-2.37	1.00	-2.33	Kcnj13	potassium inwardly-rectifying channel, subfamily J, member 13
**AI137471**	1.23	1.00	-2.33	Rtn4rl1	reticulon 4 receptor-like 1
**NM_017185.1**	1.92	1.00	-2.42	Tnn12	troponin I type 2 (skeletal, fast)
**NM_031747.1**	1.35	1.00	-2.45	Cnn1	calponin 1, basic, smooth muscle
**NM_030998.1**	1.49	1.00	-2.49	Amhr2	anti-Mullerian hormone receptor, type II
**AI235942**	1.40	1.00	-2.56	Aqp4	aquaporin 4
**BE106398**	2.34	1.00	-2.57	Lhfpl3	lipoma HMGIC fusion partner-like 3
**AW918417**	1.23	1.00	-2.62	Null	EST349721
**AI236696**	1.23	1.00	-2.81	Cox18	COX18 cytochrome c oxidase assembly homolog
**AI406939**	2.31	1.00	-2.81	G0s2	G0/G1switch 2
**AW528864**	-1.57	1.00	-2.82	Null	UI-R-BT1-AKJ-E-03-0-UI.S1 UI-R-BT1
**U25746**	-1.19	1.00	-2.85	Ddx46	DEAD (Asp-Glu-Ala-Asp) box polypeptide 46
**M96674**	-2.10	1.00	-3.09	Gcgr	glucagon receptor
**AI412189**	-20.79	1.00	-3.38	Igha_mapped	immunoglobulin heavy chain 2 (serum IgA)
**AA819268**	-1.51	1.00	-3.73	Null	UI-R-A0-AL-D-12-0-UI.S2 UI-R-A0
**BE111361**	-1.31	1.00	-4.61	RGD1306717_predicted	similar to hypothetical protein MGC25461
**AI556066**	-4.35	1.00	-9.43	Mybpc2_predicted	myosin binding protein C, fast type
**BE098709**	-1.05	1.00	-20.49	Null	UI-R-BJ1-ATD-A-06-0-UI.S1 UI-R-BJ1
**AW434308**	-36.90	1.00	-68.49	C12orf44	chromosome 12 open reading frame 44
**NM_017025.1**	1.16	1.00	-291.55	Ldha	lactate dehydrogenase A
