# Supplementary material for: The Extract of Ginkgo biloba EGb 761 Reactivates a Juvenile Profile in the Skeletal Muscle of Sarcopenic Rats by Transcriptional Reprogramming
Source: PLoS One. 2009 Nov 24;4(11):e7998. doi: 10.1371/journal.pone.0007998 (PMC2778626; doi:10.1371/journal.pone.0007998)
Supplement: Table S3 — (1.10 MB PDF) [file pone.0007998.s003.pdf]

Table S3: Cluster A

Accession	Young	Aged Control	Aged Treated	Symbol	Description
**AW913978**	13.23	1	12.42	Dennd1b	DENN/MADD domain containing 1B
**AA818947**	3.69	1	4.12	Null	UI-R-A0-AS-D-12-0-UI.S1 UI-R-A0
**M24327**	3.21	1	3.81	Mt1f	metallothionein 1F
**NM_013167.1**	3.09	1	3.15	Ucp3	uncoupling protein 3 (mitochondrial, proton carrier)
**NM_031561.1**	1.61	1	3.06	Cd36	CD36 molecule (thrombospondin receptor)
**BF551318**	1.69	1	2.72	Herc4	hect domain and RLD 4
**AI170394**	2.48	1	2.65	Null	EST216320 NORMALIZED RAT LUNG, BENTO SOARES
**BF550748**	3.30	1	2.29	Dsp	desmoplakin
**AW918622**	2.25	1	2.21	Null	EST349926
**U30789**	1.67	1	2.17	Vdup1; MGC94673	thioredoxin interacting protein
**AA874838**	1.50	1	2.05	Znf608	zinc finger protein 608
**AA892824**	1.75	1	2.01	Tnc	tenascin C (hexabrachion)
**Z78279**	3.67	1	1.93	Col1a1	collagen, type I, alpha 1
**BF402407**	1.81	1	1.84	Rhobtb2	Rho-related BTB domain containing 2
**AI235503**	1.55	1	1.80	Null	EST232065 NORMALIZED RAT OVARY, BENTO SOARES
**NM_131907.1**	1.36	1	1.77	Atp2c1	ATPase, Ca++ transporting, type 2C, member 1
**U06230**	1.88	1	1.75	Pros	protein S
**BF282483**	1.61	1	1.72	Col6a3_Predicted	collagen, type VI, alpha 3
**AF205635**	1.94	1	1.62	Cdc42	similar to cell division cycle 42
**AW915491**	1.87	1	1.59	Kab	centrosomal protein 170kDa
**BF283631**	2.49	1	1.57	Stk38	serine/threonine kinase 38
**NM_013135.1**	1.51	1	1.57	Rasa1	RAS p21 protein activator (GTPase activating protein) 1
**AI146056**	1.86	1	1.55	Nxph3	neurexophilin 3
**AF016047**	1.72	1	1.53	Pafah1b3	platelet-activating factor acetylhydrolase, isoform Ib, gamma subunit 29kDa
**AF387513**	1.45	1	1.49	Bambi	BMP and activin membrane-bound inhibitor homolog (Xenopus laevis)
**NM_012659.1**	1.69	1	1.48	Sst	somatostatin
**AA892496**	2.01	1	1.47	Null	EST196299 NORMALIZED RAT KIDNEY, BENTO SOARES
**NM_053404.1**	1.58	1	1.45	Dctn4	dynactin 4 (p62)
**NM_021760.1**	3.17	1	1.44	Col5a3	collagen, type V, alpha 3
**BE112781**	1.54	1	1.44	C10orf78	chromosome 10 open reading frame 78
**X00469**	2.22	1	1.42	Ahh	cytochrome P450, family 1, subfamily A, polypeptide 1

Table S3: Cluster A

Accession	Young	Aged Control	Aged Treated	Symbol	Description
**NM_017092.1**	1.88	1	1.42	Tyro3	TYRO3 protein tyrosine kinase
**NM_012511.1**	1.83	1	1.42	Atp7b	ATPase, Cu++ transporting, beta polypeptide
**BE113966**	1.50	1	1.41	Gmfg	glia maturation factor, gamma
**NM_012682.1**	1.30	1	1.38	Ucp1	uncouling protein 1
**AW918358**	1.50	1	1.31	C13orf1	chromosome 13 open reading frame 1
**U70050**	-1.68	1	-1.42	Jag2	jagged 2
**NM_019205.1**	-2.34	1	-1.44	Scya11	chemokine (C-C motif) ligand 11
**NM_032616.1**	-1.71	1	-1.46	Lsr	lipolysis stimulated lipoprotein receptor
**AI717140**	-2.82	1	-1.47	Tmem179	transmembrane protein 179
**AI030179**	-1.45	1	-1.50	Atp6v0e2	ATPase, H+ transporting V0 subunit e2
**BF420074**	-1.52	1	-1.50	Null	UI-R-BJ2-BPM-F-05-0-UI.S1 UI-R-BJ2
**AW914913**	-2.44	1	-1.51	Loc679949	RIKEN cDNA 3110006E14 gene
**Y15054**	-1.45	1	-1.55	Coro7	coronin 7
**NM_012676.1**	-5.49	1	-1.57	Tnnt2	troponin T type 2 (cardiac)
**AW915595**	-3.98	1	-1.58	Adipoq	adiponectin, C1Q and collagen domain containing
**NM_017139.1**	-4.31	1	-1.58	Penk	proenkephalin
**AI104125**	-1.83	1	-1.60	Mrps27_Predicted	mitochondrial ribosomal protein S27
**BF551250**	-2.09	1	-1.61	Fkbp5	FK506 binding protein 5
**NM_031819.1**	-2.35	1	-1.64	Fat	FAT tumor suppressor homolog 1 (Drosophila)
**BE100748**	-5.29	1	-1.67	C13orf30	chromosome 13 open reading frame 30
**BE097102**	-1.57	1	-1.68	Dusp8_Predicted	dual specificity phosphatase 8
**NM_133525.1**	-1.71	1	-1.81	C6orf108	chromosome 6 open reading frame 108
**NM_032071.1**	-1.68	1	-1.82	Synj2	synaptojanin 2
**NM_031549.1**	-1.61	1	-1.85	Tagln	transgelin
**AI232784**	-1.77	1	-1.89	Bdh2_Predicted	3-hydroxybutyrate dehydrogenase, type 2
**NM_057190.1**	-2.01	1	-1.92	Nelf	nasal embryonic LHRH factor
**NM_017332.1**	-1.76	1	-1.93	Fasn	fatty acid synthase
**AW915585**	-1.42	1	-1.96	Cdc42ep1_Predicted	CDC42 effector protein (Rho GTPase binding) 1
**AW531805**	-1.72	1	-2.27	Ifit3	interferon-induced protein with tetratricopeptide repeats 3
**NM_053608.1**	-2.37	1	-2.33	Kcnj13	potassium inwardly-rectifying channel, subfamily J, member 13
**AW528864**	-1.57	1	-2.82	Null	UI-R-BT1-AKJ-E-03-0-UI.S1 UI-R-BT1

Table S3: Cluster A

Accession	Young	Aged Control	Aged Treated	Symbol	Description
**AI412189**	-20.79	1	-3.38	Null	EST240483 NORMALIZED RAT KIDNEY, BENTO SOARES
**AW434308**	-36.90	1	-68.49	C12orf44	chromosome 12 open reading frame 44
