# Supplementary material for: The Extract of Ginkgo biloba EGb 761 Reactivates a Juvenile Profile in the Skeletal Muscle of Sarcopenic Rats by Transcriptional Reprogramming
Source: PLoS One. 2009 Nov 24;4(11):e7998. doi: 10.1371/journal.pone.0007998 (PMC2778626; doi:10.1371/journal.pone.0007998)
Supplement: Table S4 — (0.85 MB PDF) [file pone.0007998.s004.pdf]

Table S4: Cluster B

Accession	Young	Aged Control	Aged Treated	Symbol	Description
**NM_031813.1**	-1.71	1	2.77	Mybph	myosin binding protein H
**NM_012488.1**	-1.36	1	2.46	A2m	alpha-2-macroglobulin
**AW524724**	-2.39	1	2.43	Ryr3	ryanodine receptor 3
**NM_031544.1**	-1.82	1	2.06	Ampd3	adenosine monophosphate deaminase (isoform E)
**AI102517**	-3.22	1	1.98	Null	EST211806 NORMALIZED RAT EMBRYO, BENTO SOARES
**NM_012604.1**	-1.93	1	1.84	Myh3	myosin, heavy chain 3, skeletal muscle, embryonic
**NM_053718.1**	-2.41	1	1.82	Mllt3	myeloid/lymphoid or mixed-lineage leukemia
**BF545958**	-1.64	1	1.73	Null	UI-R-C2P-RD-B-09-0-UI.R1
**M92340**	-1.58	1	1.72	Il6st	interleukin 6 signal transducer (gp130, oncostatin M receptor)
**BE109381**	-1.60	1	1.72	H6pd	hexose-6-phosphate dehydrogenase (glucose 1-dehydrogenase)
**NM_012561.1**	-2.27	1	1.65	Fst	follistatin
**AI013474**	-2.09	1	1.64	Abhd2_Predicted	abhydrolase domain containing 2
**BE109520**	-1.37	1	1.58	Null	UI-R-BJ1-AVP-B-05-0-UI.S1 UI-R-BJ1
**AW252087**	-1.80	1	1.57	Hspb7	heat shock 27kDa protein family, member 7 (cardiovascular)
**NM_031131.1**	-1.35	1	1.57	Tgfb2	transforming growth factor, beta 2
**Y16774**	-1.45	1	1.57	Slc30a4	solute carrier family 30 (zinc transporter), member 4
**NM_019242.1**	-1.69	1	1.53	Ifrd1	interferon-related developmental regulator 1
**U77880**	-1.94	1	1.52	Pde7a	phosphodiesterase 7A
**AI008390**	-1.60	1	1.52	Vash2	vasohibin 2
**AF059530**	-1.53	1	1.50	Prmt3	protein arginine methyltransferase 3
**AA892770**	-1.78	1	1.47	Gclc	glutamate-cysteine ligase, catalytic subunit
**NM_017131.1**	-2.02	1	1.45	Casq2	calsequestrin 2 (cardiac muscle)
**AI176515**	-1.66	1	1.44	Filip1	filamin A interacting protein 1
**AW915009**	-1.77	1	1.42	Null	EST346313
**AA800241**	-1.38	1	1.40	Arl6ip5	ADP-ribosylation-like factor 6 interacting protein 5
**BF388434**	-1.48	1	1.39	Loc690541	hypothetical protein LOC690541
**AA818120**	-1.56	1	1.36	Sln	sarcolipin
**AW528057**	1.64	1	-1.39	Rragb	Ras-related GTP binding B
**AI013475**	1.37	1	-1.40	Sort1	sortilin 1
**AI172271**	1.80	1	-1.50	Emcn	endomucin
**NM_130433.1**	1.58	1	-1.51	Acaa2	acetyl-Coenzyme A acyltransferase 2

Table S4: Cluster B

Accession	Young	Aged Control	Aged Treated	Symbol	Description
**AA955175**	1.92	1	-1.57	Null	UI-R-A1-DU-B-04-0-UI.S1 UI-R-A1
**AW915996**	1.57	1	-1.58	Adamtsl2	ADAMTS-like 2
**NM_053605.1**	1.79	1	-1.62	Smpd3	sphingomyelin phosphodiesterase 3, neutral membrane
**AI406984**	1.99	1	-1.62	Null	EST235272 NORMALIZED RAT OVARY, BENTO SOARES
**AA945750**	1.66	1	-1.64	Null	EST201249 NORMALIZED RAT LUNG, BENTO SOARES
**AI176713**	1.59	1	-1.73	Dlc1	deleted in liver cancer 1
**NM_017084.1**	2.82	1	-1.75	Gnmt	glycine N-methyltransferase
**AI111863**	1.36	1	-1.76	Null	UI-R-Y0-MP-F-02-0-UI.S1 UI-R-Y0
**AI105417**	2.56	1	-1.81	C5orf13	chromosome 5 open reading frame 13
**BE109637**	1.83	1	-1.81	Fn3k	fructosamine 3 kinase
**AI410818**	2.09	1	-1.81	Null	EST239111 NORMALIZED RAT HEART, BENTO SOARES
**BE118454**	2.68	1	-1.88	C9orf24	chromosome 9 open reading frame 24
**AA817759**	2.49	1	-2.04	Slc25a25	solute carrier family 25
**NM_012797.1**	1.58	1	-2.08	Id1	inhibitor of DNA binding 1, dominant negative helix-loop-helix protein
**NM_013101.1**	2.76	1	-2.17	Pde4a	phosphodiesterase 4A, cAMP-specific
**BF387347**	3.05	1	-2.22	Null	UI-R-CA1-BBR-D-06-0-UI.S1 UI-R-CA1
**AI169829**	2.44	1	-2.26	Masp1	mannan-binding lectin serine peptidase 1
**AF325671**	3.69	1	-2.26	Kcnk2	potassium channel, subfamily K, member 2
**NM_012621.1**	1.49	1	-2.27	Pfkfb1	6-phosphofructo-2-kinase/fructose-2,6-biphosphatase 1
**NM_030998.1**	1.49	1	-2.49	Amhr2	anti-Mullerian hormone receptor, type II
