# Supplementary material for: The Extract of Ginkgo biloba EGb 761 Reactivates a Juvenile Profile in the Skeletal Muscle of Sarcopenic Rats by Transcriptional Reprogramming
Source: PLoS One. 2009 Nov 24;4(11):e7998. doi: 10.1371/journal.pone.0007998 (PMC2778626; doi:10.1371/journal.pone.0007998)
Supplement: Table S5 — (1.12 MB PDF) [file pone.0007998.s005.pdf]

Table S5: Cluster C

| Accession | Young | Aged Control | Aged Treated | Symbol | Description |
|---|---|---|---|---|---|
| **U53184** | 1.05 | 1 | 2.64 | Litaf | lipopolysaccharide-induced TNF factor |
| **NM_053978.1** | 1.16 | 1 | 2.46 | Rab28 | RAB28, member RAS oncogene family |
| **NM_031832.1** | -1.04 | 1 | 2.42 | Lgals3 | lectin, galactoside-binding, soluble, 3 |
| **NM_053551.1** | -1.28 | 1 | 2.25 | Pdk4 | pyruvate dehydrogenase kinase, isozyme 4 |
| **NM_021693.1** | 1.02 | 1 | 2.17 | Snf1lk | SNF1-like kinase |
| **BF281619** | -1.06 | 1 | 2.05 | Null | EST446210 RAT GENE INDEX, NORMALIZED RAT, |
| **AI008409** | -1.07 | 1 | 1.81 | Null | EST202860 NORMALIZED RAT EMBRYO, BENTO SOARES |
| **BF283305** | 1.09 | 1 | 1.78 | Kctd9_Predicted | potassium channel tetramerisation domain containing 9 |
| **BE110633** | 1.02 | 1 | 1.77 | Loc690541 | hypothetical protein LOC690541 |
| **X57523** | -1.04 | 1 | 1.70 | Tap1 | transporter 1, ATP-binding cassette, sub-family B (MDR/TAP) |
| **NM_024369.1** | 1.13 | 1 | 1.69 | Fstl1 | follistatin-like 1 |
| **BF398182** | -1.01 | 1 | 1.68 | Null | UI-R-BS2-BEN-D-03-0-UI.S1 UI-R-BS2 |
| **BF409831** | 1.06 | 1 | 1.67 | Epm2aip1_Predicted | EPM2A (laforin) interacting protein 1 |
| **NM_031837.1** | 1.16 | 1 | 1.65 | sept-09 | septin 9 |
| **AI231210** | -1.04 | 1 | 1.65 | Polr3b | polymerase (RNA) III (DNA directed) polypeptide B |
| **NM_053352.1** | -1.20 | 1 | 1.63 | Cxcr7 | chemokine (C-X-C motif) receptor 7 |
| **X67788** | 1.00 | 1 | 1.63 | Ezr | ezrin |
| **AW527606** | -1.08 | 1 | 1.61 | Null | UI-R-BT1-AJZ-B-10-0-UI.S1 UI-R-BT1 |
| **AI170679** | -1.10 | 1 | 1.59 | Ugp2 | UDP-glucose pyrophosphorylase 2 |
| **AA946434** | 1.09 | 1 | 1.53 | Manba | mannosidase, beta A, lysosomal |
| **AW529298** | 1.03 | 1 | 1.52 | Trafd1 | TRAF-type zinc finger domain containing 1 |
| **AA850347** | -1.04 | 1 | 1.50 | Chp | calcium binding protein P22 |
| **NM_031020.1** | -1.09 | 1 | 1.46 | Mapk14 | mitogen-activated protein kinase 14 |
| **BF567496** | 1.06 | 1 | 1.42 | Loc362845 | cDNA sequence BC066107 |
| **L18889** | -1.09 | 1 | 1.41 | Canx | calnexin |
| **AF056034** | -1.12 | 1 | 1.41 | Nexn | nexilin (F actin binding protein) |
| **AW919336** | 1.00 | 1 | 1.40 | Rad23b | RAD23 homolog B (S. cerevisiae) |
| **AI409899** | -1.18 | 1 | 1.39 | Slc20a2 | solute carrier family 20 (phosphate transporter), member 2 |
| **NM_053998.1** | -1.09 | 1 | 1.38 | Rab8a | RAB8A, member RAS oncogene family |
| **NM_031627.1** | -1.19 | 1 | 1.38 | Nr1h3 | nuclear receptor subfamily 1, group H, member 3 |
| **AW919920** | 1.15 | 1 | -1.30 | Hspa12b_Predicted | heat shock 70kD protein 12B |

Table S5: Cluster C

| Accession | Young | Aged Control | Aged Treated | Symbol | Description |
|---|---|---|---|---|---|
| **AA851256** | 1.07 | 1 | -1.32 | Null | EST194024 NORMALIZED RAT PLACENTA, BENTO SOARES |
| **NM_133526.1** | 1.09 | 1 | -1.36 | Tspan8 | tetraspanin 8 |
| **NM_012734.1** | -1.13 | 1 | -1.39 | Hk1 | hexokinase 1 |
| **BF413334** | -1.02 | 1 | -1.40 | Null | UI-R-BT1-BNW-G-06-0-UI.S1 UI-R-BT1 |
| **AW915558** | 1.08 | 1 | -1.40 | Tchp_Predicted | trichoplein, keratin filament binding |
| **AW143189** | 1.26 | 1 | -1.41 | Rasgrp3 | RAS guanyl releasing protein 3 (calcium and DAG-regulated) |
| **NM_012634.1** | -1.02 | 1 | -1.43 | Prps2 | phosphoribosyl pyrophosphate synthetase 2 |
| **BE100607** | 1.12 | 1 | -1.44 | Pitpnm1 | phosphatidylinositol transfer protein, membrane-associated 1 |
| **NM_019156.1** | 1.11 | 1 | -1.44 | Vtn | vitronectin |
| **AI177140** | 1.25 | 1 | -1.46 | Npy1r | neuropeptide Y receptor Y1 |
| **AI231193** | -1.05 | 1 | -1.47 | Null | EST227881 NORMALIZED RAT EMBRYO, BENTO SOARES |
| **AJ225626** | 1.09 | 1 | -1.47 | Null | RAT LIVER ESTS (E.OLIVIER) |
| **AW523746** | 1.13 | 1 | -1.50 | C2orf40 | chromosome 2 open reading frame 40 |
| **AI059108** | 1.03 | 1 | -1.50 | Null | UI-R-C1-LR-F-03-0-UI.S1 UI-R-C1 |
| **NM_030844.1** | 1.05 | 1 | -1.51 | Ica1 | islet cell autoantigen 1, 69kDa |
| **AI227996** | 1.27 | 1 | -1.51 | Gm1123 | gene model 1123, (NCBI) |
| **AA799656** | 1.07 | 1 | -1.52 | Mrps31_Predicted | mitochondrial ribosomal protein S31 |
| **AW253265** | 1.08 | 1 | -1.56 | Etv6 | ets variant gene 6 (TEL oncogene) |
| **AA946128** | 1.14 | 1 | -1.56 | Icoslg | inducible T-cell co-stimulator ligand |
| **J03959** | 1.33 | 1 | -1.57 | Uox-2 | urate oxidase (pseudogene) |
| **BF555099** | 1.11 | 1 | -1.58 | Ttll5 | tubulin tyrosine ligase-like family, member 5 |
| **U44948** | -1.05 | 1 | -1.62 | Csrp2 | cysteine and glycine-rich protein 2 |
| **AA848826** | -1.07 | 1 | -1.63 | Asah3l | N-acylsphingosine amidohydrolase 3-like |
| **NM_031747.1** | 1.00 | 1 | -1.63 | Cnn1 | calponin 1, basic, smooth muscle |
| **D25290** | 1.01 | 1 | -1.64 | Cdh6 | cadherin 6, type 2, K-cadherin (fetal kidney) |
| **BI282127** | 1.17 | 1 | -1.65 | Hint2_Predicted | histidine triad nucleotide binding protein 2 |
| **AI234142** | -1.01 | 1 | -1.67 | Iqcb1_Predicted | IQ motif containing B1 |
| **BF398114** | -1.21 | 1 | -1.68 | Null | UI-R-BS2-BEM-E-10-0-UI.S1 UI-R-BS2 |
| **BF410042** | 1.15 | 1 | -1.68 | Null | UI-R-CA0-BJS-H-01-0-UI.S1 UI-R-CA0 |
| **AW527473** | 1.09 | 1 | -1.68 | Null | UI-R-BO1-AJT-G-02-0-UI.S1 UI-R-BO1 |
| **NM_017136.1** | 1.01 | 1 | -1.69 | Sqle | squalene epoxidase |

Table S5: Cluster C

| Accession | Young | Aged Control | Aged Treated | Symbol | Description |
|---|---|---|---|---|---|
| **AA943573** | 1.11 | 1 | -1.69 | Gla | galactosidase, alpha |
| **AI411141** | 1.15 | 1 | -1.73 | Loc501038 | Ab2-060 |
| **AF000942.1** | 1.16 | 1 | -1.73 | Id3a | inhibitor of DNA binding 3, dominant negative helix-loop-helix protein |
| **AB025017** | 1.16 | 1 | -1.73 | Zfp36 | zinc finger protein 36, C3H type, homolog (mouse) |
| **NM_024364.1** | -1.12 | 1 | -1.75 | Hr | hairless homolog (mouse) |
| **AW526033** | 1.12 | 1 | -1.75 | Null | UI-R-BO1-AIY-E-04-0-UI.S1 UI-R-BO1 |
| **NM_012731.1** | -1.08 | 1 | -1.76 | Ntrk2 | neurotrophic tyrosine kinase, receptor, type 2 |
| **NM_019350.1** | -1.07 | 1 | -1.77 | Syt5 | synaptotagmin V |
| **BF291161** | 1.10 | 1 | -1.81 | Null | EST455752 RAT GENE INDEX, NORMALIZED RAT, |
| **AW254246** | 1.15 | 1 | -1.84 | Null | UI-R-BJ0-AEV-D-04-0-UI.S1 UI-R-BJ0 |
| **NM_013058.1** | 1.06 | 1 | -1.87 | Id3 | inhibitor of DNA binding 3, dominant negative helix-loop-helix protein |
| **BG664142** | 1.02 | 1 | -1.89 | C1orf71 | chromosome 1 open reading frame 71 |
| **AW435010** | 1.10 | 1 | -1.89 | Ptpn3 | protein tyrosine phosphatase, non-receptor type 3 |
| **NM_012884.1** | -1.12 | 1 | -1.92 | Cntn2 | contactin 2 (axonal) |
| **NM_080892.1** | -1.10 | 1 | -1.92 | Selenbp1 | selenium binding protein 1 |
| **AW916151** | -1.02 | 1 | -1.92 | Xkr8 | XK, Kell blood group complex subunit-related family, member 8 |
| **AI137286** | -1.30 | 1 | -1.96 | Null | UI-R-C2P-OL-A-06-0-UI.S1 UI-R-C2P |
| **NM_013190.1** | -1.12 | 1 | -2.02 | Pfkl | phosphofructokinase, liver |
| **AI145761** | 1.15 | 1 | -2.05 | Ldhd | lactate dehydrogenase D |
| **NM_017025.1** | 1.16 | 1 | -291.55 | Ldha | lactate dehydrogenase A |
