# Supplementary material for: The Extract of Ginkgo biloba EGb 761 Reactivates a Juvenile Profile in the Skeletal Muscle of Sarcopenic Rats by Transcriptional Reprogramming
Source: PLoS One. 2009 Nov 24;4(11):e7998. doi: 10.1371/journal.pone.0007998 (PMC2778626; doi:10.1371/journal.pone.0007998)
Supplement: Table S6 — (1.39 MB PDF) [file pone.0007998.s006.pdf]

Table S6: Cluster D

Accession	Young	Aged Control	Aged Treated	Symbol	Description
**AW251681**	1	7.68	7.52	Null	UI-R-BJ0-ADP-F-06-0-UI.S1 UI-R-BJ0 RATTUS NORVEGICUS CDNA CLONE UI-R-BJ0-ADP-F-06-0-UI
**AW915407**	1	6.46	7.69	Null	EST346711 RATTUS NORVEGICUS CDNA
**BE113616**	1	3.73	2.84	C4orf19	chromosome 4 open reading frame 19
**NM_133424.1**	1	3.69	3.43	Actn3	actinin, alpha 3
**AA818377**	1	3.22	4.20	Zfp533_Predicted	zinc finger protein 385B
**AI409738**	1	2.97	3.33	Null	EST238030 NORMALIZED RAT PLACENTA BENTO SOARES RATTUS SP. CDNA CLONE RPLDI30
**NM_012861.1**	1	2.77	2.35	Mgmt	O-6-methylguanine-DNA methyltransferase
**NM_031143.1**	1	2.61	2.21	Dgkz	diacylglycerol kinase, zeta 104kDa
**NM_012827.1**	1	2.39	1.99	Bmp4	bone morphogenetic protein 4
**NM_017288.1**	1	2.39	2.15	Scn1b	sodium channel, voltage-gated, type I, beta
**BF407456**	1	2.35	2.57	Obfc1	oligonucleotide/oligosaccharide-binding fold containing 1
**NM_019298.1**	1	2.28	2.83	Chrnd	cholinergic receptor, nicotinic, delta
**NM_024384.1**	1	2.25	2.25	Thoc6	THO complex 6 homolog (Drosophila)
**AW917185**	1	2.25	2.43	Armet_Predicted	arginine-rich, mutated in early stage tumors
**AW919170**	1	2.23	2.69	Raly	RNA binding protein, autoantigenic (hnRNP-associated with lethal yellow homolog (mouse))
**AW433866**	1	2.19	2.12	Null	UI-R-BJ0P-AFM-E-09-0-UI.S1 UI-R-BJ0P RATTUS NORVEGICUS CDNA CLONE UI-R-BJ0P-AFM-E-09-0-UI
**AI600036**	1	2.19	2.01	Null	EST251739 NORMALIZED RAT EMBRYO BENTO SOARES RATTUS SP. CDNA CLONE REMDO74
**AI230729**	1	2.17	2.16	Zfp513	zinc finger protein 513
**NM_053612.1**	1	2.11	2.62	Hspb8	heat shock 22kDa protein 8
**NM_012531.1**	1	2.09	2.25	Comt	catechol-O-methyltransferase
**BE106888**	1	2.07	2.31	Creld2	cysteine-rich with EGF-like domains 2
**NM_030865.1**	1	2.07	2.05	Myoc	myocilin, trabecular meshwork inducible glucocorticoid response
**AI230728**	1	2.01	1.95	Snrpa	small nuclear ribonucleoprotein polypeptide A
**BE106058**	1	2.00	1.93	Null	UI-R-BO1-ASK-D-07-0-UI.S1 UI-R-BO1 RATTUS NORVEGICUS CDNA CLONE UI-R-BO1-ASK-D-07-0-UI
**AA850544**	1	1.99	2.39	Inpp5a_Predicted	inositol polyphosphate-5-phosphatase, 40kDa
**BE111776**	1	1.96	1.82	Loc311134	hypothetical protein BC014011
**AF112256**	1	1.94	2.29	Ryr1	ryanodine receptor 1, skeletal muscle
**H35261**	1	1.93	1.75	Null	EST104829 RAT PC-12 CELLS UNTREATED RATTUS SP. CDNA CLONE RPCAB71
**AI233343**	1	1.90	1.47	Asrgl1	asparaginase like 1
**AI408984**	1	1.90	1.89	Rfwd3	ring finger and WD repeat domain 3
**AW143263**	1	1.87	2.12	Rich2	Rho-type GTPase-activating protein RICH2

Table S6: Cluster D

Accession	Young	Aged Control	Aged Treated	Symbol	Description
**AI548760**	1	1.87	1.81	Ngdn	neuroguidin, EIF4E binding protein
**NM_020106.1**	1	1.83	1.82	Or1n1	olfactory receptor, family 1, subfamily N, member 1
**AA848958**	1	1.81	1.58	Null	EST191720 NORMALIZED RAT LUNG BENTO SOARES RATTUS SP. CDNA CLONE RLUAI41
**U76557**	1	1.80	1.86	Ogt	O-linked N-acetylglucosamine (GlcNAc) transferase
**NM_021863.1**	1	1.79	1.57	Hspa2	heat shock 70kDa protein 2
**AA946349**	1	1.76	1.49	Nudt3	nudix (nucleoside diphosphate linked moiety X)-type motif 3
**BF282282**	1	1.75	1.58	Psph	phosphoserine phosphatase
**AI600237**	1	1.73	1.70	Eef1e1_Predicted	eukaryotic translation elongation factor 1 epsilon 1
**AI407490**	1	1.73	1.62	Yars	tyrosyl-tRNA synthetase
**AA891839**	1	1.72	1.65	Mrpl50_Predicted	mitochondrial ribosomal protein L50
**NM_012811.1**	1	1.72	1.58	Mgfe8	milk fat globule-EGF factor 8 protein
**AI410050**	1	1.71	1.78	Med24	mediator complex subunit 24
**BE121438**	1	1.71	1.99	Lig4_Predicted	ligase IV, DNA, ATP-dependent
**NM_030868.1**	1	1.71	1.84	Nov	nephroblastoma overexpressed gene
**NM_017288.1**	1	1.69	1.48	Scn1b	sodium channel, voltage-gated, type I, beta
**NM_138548.1**	1	1.67	1.48	Nme1	non-metastatic cells 1, protein (NM23A) expressed in
**U81160**	1	1.67	1.54	Vps45	vacuolar protein sorting 45 homolog (S. cerevisiae)
**AW919062**	1	1.67	1.44	Slc9a8	solute carrier family 9 (sodium/hydrogen exchanger), member 8
**AI412736**	1	1.66	1.65	Tomm34_Predicted	translocase of outer mitochondrial membrane 34
**AA945771**	1	1.66	1.79	Null	EST201270 NORMALIZED RAT LUNG BENTO SOARES RATTUS SP. CDNA CLONE RLUAU09
**AW520823**	1	1.65	1.56	Amotl1_Predicted	angiomotin like 1
**AA818571**	1	1.65	2.07	Null	UI-R-A0-AW-H-09-0-UI.S1 UI-R-A0 RATTUS NORVEGICUS CDNA CLONE UI-R-A0-AW-H-09-0-UI
**NM_031139.1**	1	1.64	1.68	Usf2	upstream transcription factor 2, c-fos interacting
**BF393949**	1	1.63	1.77	Sec13	SEC13 homolog (S. cerevisiae)
**AJ245648**	1	1.63	1.52	Pola2	polymerase (DNA directed), alpha 2 (70kD subunit)
**AI716516**	1	1.63	1.47	Null	UI-R-Y0-ABI-A-06-0-UI.S1 UI-R-Y0 RATTUS NORVEGICUS CDNA CLONE UI-R-Y0-ABI-A-06-0-UI
**AA848834**	1	1.62	2.10	Dhdds	dehydrodolichyl diphosphate synthase
**NM_031967.1**	1	1.61	1.94	Ndrg4	NDRG family member 4
**AF156878**	1	1.61	1.66	Ogfr	opioid growth factor receptor
**NM_053959.1**	1	1.60	1.57	Bin1	bridging integrator 1
**AW143082**	1	1.59	1.82	Null	EST293378 RATTUS NORVEGICUS CDNA

Table S6: Cluster D

Accession	Young	Aged Control	Aged Treated	Symbol	Description
**AA799515**	1	1.58	1.65	Wsb2	WD repeat and SOCS box-containing 2
**BE096387**	1	1.58	1.80	Loc288526	similar to DNA segment on chromosome X and Y (unique) 155 expressed sequence isoform 1
**AW920343**	1	1.58	1.52	Null	EST351647 RAT GENE
**AW918153**	1	1.57	1.75	Null	EST349457 RATTUS NORVEGICUS CDNA
**AW528874**	1	1.57	1.61	Hirip3	HIRA interacting protein 3
**NM_033230.1**	1	1.55	1.71	Akt1	v-akt murine thymoma viral oncogene homolog 1
**BE101435**	1	1.54	1.81	Null	UI-R-BJ1-AUJ-A-02-0-UI.S1 UI-R-BJ1 RATTUS NORVEGICUS CDNA CLONE UI-R-BJ1-AUJ-A-02-0-UI
**BE128566**	1	1.54	1.60	Copz2_Predicted	coatomer protein complex, subunit zeta 2 (predicted)
**BF568015**	1	1.54	1.81	Ebpl_Predicted	emopamil binding protein-like
**AI411991**	1	1.54	1.59	Ndrg1	N-myc downstream regulated gene 1
**AA819192**	1	1.53	1.73	Null	UI-R-A0-AB-H-08-0-UI.S2 UI-R-A0 RATTUS NORVEGICUS CDNA CLONE UI-R-A0-AB-H-08-0-UI
**AW253263**	1	1.53	1.82	Rtel1	regulator of telomere elongation helicase 1
**NM_017117.1**	1	1.53	1.58	Capn3	calpain 3, (p94)
**NM_053543.1**	1	1.52	1.58	Ncdn-Pending	neurochondrin
**AI406310**	1	1.52	1.67	Null	EST234596 NORMALIZED RAT BRAIN BENTO SOARES RATTUS SP. CDNA CLONE RBRDM66
**NM_080886.1**	1	1.52	1.77	Sc4mol	sterol-C4-methyl oxidase-like
**NM_012591.1**	1	1.51	1.47	Irf1	interferon regulatory factor 1
**BE119991**	1	1.50	1.48	Frmpd1_Predicted	FERM and PDZ domain containing 1
**BF398684**	1	1.49	1.55	Null	UI-R-BS2-BER-F-08-0-UI.S1 UI-R-BS2 RATTUS NORVEGICUS CDNA CLONE UI-R-BS2-BER-F-08-0-UI
**AW915012**	1	1.48	1.37	Null	EST346316 RATTUS NORVEGICUS CDNA
**NM_053563.1**	1	1.47	1.56	Ddx39	DEAD (Asp-Glu-Ala-Asp) box polypeptide 39
**NM_032079.1**	1	1.47	1.57	Dnaja2	DnaJ (Hsp40) homolog, subfamily A, member 2
**NM_053929.1**	1	1.46	1.46	Slc7A9	solute carrier family 7 (cationic amino acid transporter, y+ system), member 9
**BF282876**	1	1.46	1.50	Rab11fip3	RAB11 family interacting protein 3 (class II)
**AA800364**	1	1.46	1.53	RGD1564058_Predicted	chromosome 19 open reading frame 56
**AA818759**	1	1.45	1.65	RGD1560745_Predicted	COMM domain containing 6
**AI172302**	1	1.45	1.74	Srxn1	sulfiredoxin 1 homolog (S. cerevisiae)
**BE098848**	1	1.44	1.41	Null	UI-R-BJ1-ATE-E-10-0-UI.S1 UI-R-BJ1 RATTUS NORVEGICUS CDNA CLONE UI-R-BJ1-ATE-E-10-0-UI
**BE109586**	1	1.44	1.38	Chmp6_Predicted	chromatin modifying protein 6
**BF564263**	1	1.43	1.44	Ift81	intraflagellar transport 81 homolog (Chlamydomonas)
**AI410895**	1	1.43	1.36	Null	EST239188 NORMALIZED RAT HEART BENTO SOARES RATTUS SP. CDNA CLONE RHECY77

Table S6: Cluster D

Accession	Young	Aged Control	Aged Treated	Symbol	Description
**AW441131**	1	1.43	1.69	Mycbp2	MYC binding protein 2
**NM_017125.1**	1	1.41	1.70	Cd63	CD63 molecule
**BE113057**	1	1.40	1.61	Traf3	TNF receptor-associated factor 3
**AW918610**	1	1.39	1.58	Npepps	aminopeptidase puromycin sensitive
**NM_133419.1**	1	1.39	1.56	Dkc1	dyskeratosis congenita 1, dyskerin
**AI232065**	1	1.39	1.59	Arhgap18_Predicted	Rho GTPase activating protein 18
**BE103359**	1	1.39	1.41	Kiaa0368	KIAA0368
**NM_031051.1**	1	1.38	1.42	Mif	macrophage migration inhibitory factor (glycosylation-inhibiting factor)
**NM_053373.1**	1	1.36	1.50	Pglyrp	peptidoglycan recognition protein 1
**AA800179**	1	1.34	1.55	Cox4nb	COX4 neighbor
**AI407555**	1	-1.35	-1.60	Fbxw9	F-box and WD repeat domain containing 9
**BE107737**	1	-1.35	-1.43	Crocc_Predicted	ciliary rootlet coiled-coil, rootletin
**NM_133618.1**	1	-1.37	-1.37	Hadhb	hydroxyacyl-Coenzyme A dehydrogenase, beta subunit
**AI556402**	1	-1.38	-1.46	Null	UI-R-C2P-RH-B-09-0-UI.S1 UI-R-C2P RATTUS NORVEGICUS CDNA CLONE UI-R-C2P-RH-B-09-0-UI
**BF396218**	1	-1.39	-1.42	C1orf131	chromosome 1 open reading frame 131
**AW529588**	1	-1.39	-1.37	Null	UI-R-BT1-AKN-A-06-0-UI.S1 UI-R-BT1 RATTUS NORVEGICUS CDNA CLONE UI-R-BT1-AKN-A-06-0-UI
**BF281969**	1	-1.39	-1.67	C10orf57	chromosome 10 open reading frame 57
**NM_080581.1**	1	-1.40	-1.77	Abcc	ATP-binding cassette, sub-family C (CFTR/MRP), member 3
**AB019366**	1	-1.40	-1.36	Parg	poly (ADP-ribose) glycohydrolase
**NM_021751.1**	1	-1.42	-1.55	Prom1	prominin 1
**AF268593**	1	-1.44	-1.47	Itgam	integrin, alpha M (complement component 3 receptor 3 subunit)
**AI710879**	1	-1.44	-1.67	Null	UI-R-AE1-ZH-D-03-0-UI.S1 UI-R-AE1 RATTUS NORVEGICUS CDNA CLONE UI-R-AE1-ZH-D-03-0-UI
**AI175048**	1	-1.44	-1.38	Six1	SIX homeobox 1
**NM_012816.1**	1	-1.45	-1.59	Amacr	alpha-methylacyl-CoA racemase
**AW254375**	1	-1.46	-1.48	Null	UI-R-BJ0-AEI-B-08-0-UI.S1 UI-R-BJ0 RATTUS NORVEGICUS CDNA CLONE UI-R-BJ0-AEI-B-08-0-UI
**BE110545**	1	-1.46	-1.79	Osbp_Predicted	oxysterol binding protein
**AA799358**	1	-1.46	-1.44	Dfnb31	deafness, autosomal recessive 31
**AI406341**	1	-1.46	-1.55	Null	EST234627 NORMALIZED RAT BRAIN BENTO SOARES RATTUS SP. CDNA CLONE RBRDM22
**AW520781**	1	-1.46	-1.62	Asb8_Predicted	ankyrin repeat and SOCS box-containing 8
**BE114154**	1	-1.46	-1.44	Null	UI-R-BJ1-AWJ-G-01-0-UI.S1 UI-R-BJ1 RATTUS NORVEGICUS CDNA CLONE UI-R-BJ1-AWJ-G-01-0-UI
**BF282314**	1	-1.46	-1.48	C18orf8	chromosome 18 open reading frame 8

Table S6: Cluster D

Accession	Young	Aged Control	Aged Treated	Symbol	Description
**AI170769**	1	-1.47	-1.53	RGD1306839_Predicted	chromosome 9 open reading frame 46
**BF548006**	1	-1.47	-1.58	Anapc1_Predicted	anaphase promoting complex subunit 1
**BF556273**	1	-1.47	-1.65	Vezf1_Predicted	vascular endothelial zinc finger 1
**BF399633**	1	-1.48	-1.92	Kiaa0922	KIAA0922
**AB052846**	1	-1.49	-1.57	Sc5dl	sterol-C5-desaturase (ERG3 delta-5-desaturase homolog, S. cerevisiae)-like
**NM_017110.1**	1	-1.49	-1.52	Cartpt	CART prepropeptide
**AW524559**	1	-1.49	-1.69	Null	UI-R-BO0-AHZ-G-12-0-UI.S1 UI-R-BO0 RATTUS NORVEGICUS CDNA CLONE UI-R-BO0-AHZ-G-12-0-UI
**AW531735**	1	-1.50	-1.58	Null	UI-R-C4-ALK-E-03-0-UI.S1 UI-R-C4 RATTUS NORVEGICUS CDNA CLONE UI-R-C4-ALK-E-03-0-UI
**X53232**	1	-1.50	-1.87	Kiaa1688	KIAA1688 protein
**AA817813**	1	-1.50	-1.64	Null	UI-R-A0-AE-C-09-0-UI.S1 UI-R-A0 RATTUS NORVEGICUS CDNA CLONE UI-R-A0-AE-C-09-0-UI
**AA848536**	1	-1.51	-1.58	Glrx2	glutaredoxin 2
**AW915015**	1	-1.51	-1.45	Null	EST346319 RATTUS NORVEGICUS CDNA
**L19699**	1	-1.51	-1.68	Dralb	v-ral simian leukemia viral oncogene homolog B (ras related; GTP binding protein)
**K02816**	1	-1.52	-1.46	Sub1	SUB1 homolog (S. cerevisiae)
**BE104321**	1	-1.52	-1.45	Ankrd13c	ankyrin repeat domain 13C
**BF405581**	1	-1.53	-1.53	Phf3_Predicted	PHD finger protein 3
**BF551118**	1	-1.53	-1.72	Sox17_Predicted	SRY (sex determining region Y)-box 17
**AA925922**	1	-1.53	-1.47	Null	UI-R-A1-ES-G-09-0-UI.S1 UI-R-A1 RATTUS NORVEGICUS CDNA CLONE UI-R-A1-ES-G-09-0-UI
**NM_013105.1**	1	-1.53	-1.57	Cyp3a3	cytochrome P450, family 3, subfamily A, polypeptide 4
**AW916756**	1	-1.54	-1.66	Null	EST348164 RATTUS NORVEGICUS CDNA
**BF389244**	1	-1.54	-1.56	Gins1	GINS complex subunit 1 (Psf1 homolog)
**AI010267**	1	-1.55	-1.71	Cybrd1	cytochrome b reductase 1
**BE099063**	1	-1.55	-1.75	Nradd	neurotrophin receptor associated death domain
**BE112921**	1	-1.55	-1.96	Loc500700	similar to chromosome 14 open reading frame 145
**BG666843**	1	-1.56	-1.45	Null	DRACNC05 RATTUS NORVEGICUS CDNA
**BF548520**	1	-1.56	-1.86	Null	UI-R-A0-AQ-B-01-0-UI.R1 UI-R-A0 RATTUS NORVEGICUS CDNA CLONE UI-R-A0-AQ-B-01-0-UI
**AI703715**	1	-1.56	-1.99	Prkd2	protein kinase D2
**AW917849**	1	-1.56	-1.64	Fzd6	frizzled homolog 6 (Drosophila)
**BF409371**	1	-1.56	-1.93	Eif2ak4_Predicted	eukaryotic translation initiation factor 2 alpha kinase 4
**AI233916**	1	-1.56	-1.76	Hltf	helicase-like transcription factor
**AI012474**	1	-1.57	-1.79	Agpat2_Predicted	1-acylglycerol-3-phosphate O-acyltransferase 2 (lysophosphatidic acid acyltransferase, beta)

Table S6: Cluster D

Accession	Young	Aged Control	Aged Treated	Symbol	Description
**AW523888**	1	-1.57	-1.63	Null	UI-R-BO0-AIE-B-09-0-UI.S1 UI-R-BO0 RATTUS NORVEGICUS CDNA CLONE UI-R-BO0-AIE-B-09-0-UI
**AI233865**	1	-1.57	-1.80	Null	EST230553 NORMALIZED RAT LUNG BENTO SOARES RATTUS SP. CDNA CLONE RLUCS14
**BF558902**	1	-1.57	-1.74	Nid2	nidogen 2 (osteonidogen)
**AI170351**	1	-1.57	-1.49	Dus4l_Predicted	dihydrouridine synthase 4-like (S. cerevisiae)
**NM_019296.1**	1	-1.57	-1.61	Cdc2a	cell division cycle 2, G1 to S and G2 to M
**AI317813**	1	-1.58	-1.64	Null	EST234484 PC12 CELLS UNTREATED PT7T3PAC TIGR RATTUS SP. CDNA CLONE RPPAA18
**AI227742**	1	-1.59	-1.68	Bok	BCL2-related ovarian killer
**AI411742**	1	-1.60	-1.65	Sesn1_Predicted	sestrin 1
**AW435463**	1	-1.60	-1.53	Null	UI-R-BJ0P-AFU-G-06-0-UI.S1 UI-R-BJ0P RATTUS NORVEGICUS CDNA CLONE UI-R-BJ0P-AFU-G-06-0-UI
**AA892273**	1	-1.61	-1.43	Rfx5	regulatory factor X, 5 (influences HLA class II expression)
**AI171651**	1	-1.61	-1.51	Tmem123	transmembrane protein 123
**BE107051**	1	-1.62	-1.47	Null	UI-R-BS1-AYP-C-04-0-UI.S1 UI-R-BS1 RATTUS NORVEGICUS CDNA CLONE UI-R-BS1-AYP-C-04-0-UI
**NM_012793.1**	1	-1.62	-2.02	Gamt	guanidinoacetate N-methyltransferase
**BE108249**	1	-1.63	-1.66	Null	UI-R-BS1-AYX-B-02-0-UI.S1 UI-R-BS1 RATTUS NORVEGICUS CDNA CLONE UI-R-BS1-AYX-B-02-0-UI
**BF409313**	1	-1.64	-1.66	Hddc2_Predicted	HD domain containing 2
**BF386302**	1	-1.64	-1.78	Null	UI-R-CA1-BBC-H-10-0-UI.S1 UI-R-CA1 RATTUS NORVEGICUS CDNA CLONE UI-R-CA1-BBC-H-10-0-UI
**NM_017032.1**	1	-1.65	-1.53	Pde4d	phosphodiesterase 4D, cAMP-specific (phosphodiesterase E3 dunce homolog, Drosophila)
**BF282686**	1	-1.65	-1.78	Kiaa1826	KIAA1826
**AI012390**	1	-1.65	-2.01	Null	EST206841 NORMALIZED RAT PLACENTA BENTO SOARES RATTUS SP. CDNA CLONE RPLAW55
**AI407992**	1	-1.66	-1.71	Atm	ataxia telangiectasia mutated
**BF418913**	1	-1.67	-1.49	Wwtr1	WW domain containing transcription regulator 1
**NM_022943.1**	1	-1.67	-2.04	Mertk	c-mer proto-oncogene tyrosine kinase
**BF562347**	1	-1.68	-1.81	Null	UI-R-BU0-ANC-G-01-0-UI.R1 RATTUS NORVEGICUS CDNA
**AA998252**	1	-1.69	-1.57	Null	UI-R-C0-IE-F-02-0-UI.S1 UI-R-C0 RATTUS NORVEGICUS CDNA CLONE UI-R-C0-IE-F-02-0-UI
**NM_053878.1**	1	-1.69	-1.75	Cplx2	complexin 2
**AI169596**	1	-1.69	-1.87	Loc691543	chromosome 15 open reading frame 29
**AI178196**	1	-1.70	-1.84	Bahd1	bromo adjacent homology domain containing 1
**AA946467**	1	-1.70	-1.99	Null	EST201966 NORMALIZED RAT OVARY BENTO SOARES RATTUS SP. CDNA CLONE ROVAR47
**BE116848**	1	-1.71	-1.43	Null	UI-R-BS1-AZG-A-01-0-UI.S1 UI-R-BS1 RATTUS NORVEGICUS CDNA CLONE UI-R-BS1-AZG-A-01-0-UI
**X61677**	1	-1.72	-1.58	Itpr2	inositol 1,4,5-triphosphate receptor, type 2
**NM_134453.1**	1	-1.72	-1.61	Lbr	lamin B receptor

Table S6: Cluster D

Accession	Young	Aged Control	Aged Treated	Symbol	Description
**NM_013129.1**	1	-1.72	-1.72	Il15	interleukin 15
**AI010721**	1	-1.73	-1.62	Rgd1305288	chromosome 14 open reading frame 94
**BE108162**	1	-1.73	-1.80	Glccl1	glucocorticoid induced transcript 1
**AI555457**	1	-1.73	-1.86	Ctnnal1_Predicted	catenin (cadherin-associated protein), alpha-like 1
**AI171975**	1	-1.73	-2.13	Sec24a	SEC24 related gene family, member A (S. cerevisiae)
**NM_012789.1**	1	-1.74	-1.66	Dpp4	dipeptidyl-peptidase 4 (CD26, adenosine deaminase complexing protein 2)
**BF410183**	1	-1.74	-1.86	Null	UI-R-CA1-BJU-D-12-0-UI.S1 UI-R-CA1 RATTUS NORVEGICUS CDNA CLONE UI-R-CA1-BJU-D-12-0-UI
**X76996**	1	-1.75	-1.77	Gzmb	granzyme B (granzyme 2, cytotoxic T-lymphocyte-associated serine esterase 1)
**AI411809**	1	-1.75	-2.17	Null	EST240103 NORMALIZED RAT KIDNEY BENTO SOARES RATTUS SP. CDNA CLONE RKIDN14
**AW142170**	1	-1.76	-1.92	Rev3l	REV3-like, catalytic subunit of DNA polymerase zeta (yeast)
**BE098855**	1	-1.76	-2.02	Null	UI-R-BJ1-ATE-F-05-0-UI.S1 UI-R-BJ1 RATTUS NORVEGICUS CDNA CLONE UI-R-BJ1-ATE-F-05-0-UI
**NM_080888.1**	1	-1.76	-1.65	Bn1p3l	BCL2/adenovirus E1B 19kDa interacting protein 3-like
**AW917390**	1	-1.77	-1.38	Null	EST348694 RATTUS NORVEGICUS CDNA
**AI178923**	1	-1.77	-1.94	Glcc1	glucocorticoid induced transcript 1
**NM_017161.1**	1	-1.78	-1.58	Adora2b	adenosine A2b receptor
**AW252878**	1	-1.80	-1.66	Pxdn	peroxidasin homolog (Drosophila)
**BF550401**	1	-1.80	-1.95	Cct5	chaperonin containing TCP1, subunit 5 (epsilon)
**BF290076**	1	-1.80	-1.84	Gem_Predicted	GTP binding protein overexpressed in skeletal muscle
**AI231444**	1	-1.80	-2.06	Loc289233	similar to Peroxisomal biogenesis factor 19 (Peroxin-19) (Peroxisomal farnesylated protein)
**BF403009**	1	-1.81	-1.92	Phf3	PHD finger protein 3
**BF405932**	1	-1.82	-1.74	Null	UI-R-CA1-BII-G-01-0-UI.S1 UI-R-CA1 RATTUS NORVEGICUS CDNA CLONE UI-R-CA1-BII-G-01-0-UI
**M81639**	1	-1.83	-2.06	Snn	stannin
**AW918541**	1	-1.83	-2.20	Null	EST349845 RATTUS NORVEGICUS CDNA
**AF002251**	1	-1.83	-1.81	Rassf5	Ras association (RalGDS/AF-6) domain family member 5
**AW920324**	1	-1.83	-1.88	Fat4	FAT tumor suppressor homolog 4 (Drosophila)
**NM_019186.1**	1	-1.84	-1.74	Arl4	ADP-ribosylation factor-like 4A
**NM_021669.1**	1	-1.85	-1.87	Ghrl	ghrelin/obestatin preprohormone
**BF284887**	1	-1.85	-1.90	C20orf194	chromosome 20 open reading frame 194
**BF390003**	1	-1.87	-1.50	Null	UI-R-BS2-BDR-B-09-0-UI.S1 UI-R-BS2 RATTUS NORVEGICUS CDNA CLONE UI-R-BS2-BDR-B-09-0-UI
**BE113264**	1	-1.88	-1.87	Osbpl9_Predicted	oxysterol binding protein-like 9
**BF394170**	1	-1.88	-2.08	Jundp2	Jun dimerization protein 2

Table S6: Cluster D

Accession	Young	Aged Control	Aged Treated	Symbol	Description
**AA850909**	1	-1.88	-2.25	Pvrl2	poliovirus receptor-related 2 (herpesvirus entry mediator B)
**AI176950**	1	-1.88	-1.87	Null	EST220556 NORMALIZED RAT OVARY BENTO SOARES RATTUS SP. CDNA CLONE ROVBZ03
**BF407675**	1	-1.89	-1.88	Null	UI-R-BJ2-BQV-A-11-0-UI.S1 UI-R-BJ2 RATTUS NORVEGICUS CDNA CLONE UI-R-BJ2-BQV-A-11-0-UI
**AI600255**	1	-1.89	-1.75	Rgd1305793	chromosome 10 open reading frame 26
**BF389882**	1	-1.91	-1.64	Shprh_Predicted	SNF2 histone linker PHD RING helicase
**AI179315**	1	-1.92	-2.28	B3gnt2	UDP-GlcNAc:betaGal beta-1,3-N-acetylglucosaminyltransferase 2
**AI412740**	1	-1.93	-2.18	Null	EST241039 NORMALIZED RAT BRAIN BENTO SOARES RATTUS SP. CDNA CLONE RBRDT10
**BF285068**	1	-1.96	-2.06	Ciapin1	cytokine induced apoptosis inhibitor 1
**U61729**	1	-1.97	-1.69	Pnrc1	proline-rich nuclear receptor coactivator 1
**BF289154**	1	-1.97	-1.91	Null	EST453745 RAT GENE
**AI176781**	1	-1.98	-2.30	Null	EST220376 NORMALIZED RAT OVARY BENTO SOARES RATTUS SP. CDNA CLONE ROVBW70
**AA858930**	1	-2.00	-2.16	Pde4b	phosphodiesterase 4B, cAMP-specific (phosphodiesterase E4 dunce homolog, Drosophila)
**AW143149**	1	-2.03	-1.85	Gnpda2_Predicted	glucosamine-6-phosphate deaminase 2
**BF283760**	1	-2.03	-1.93	Ldb2_Predicted	LIM domain binding 2
**NM_019372.1**	1	-2.06	-2.32	Ppm2c	protein phosphatase 2C, magnesium-dependent, catalytic subunit
**AA892554**	1	-2.08	-2.10	G3bp2	GTPase activating protein (SH3 domain) binding protein 2
**AW915638**	1	-2.08	-2.26	Mdf1c	MyoD family inhibitor domain containing
**BE111650**	1	-2.10	-1.80	Garnl1	GTPase activating Rap/RanGAP domain-like 1
**AI599484**	1	-2.11	-2.45	Loc500420	similar to hCG2030844
**NM_012612.1**	1	-2.12	-1.86	Nppa	natriuretic peptide precursor A
**BF395777**	1	-2.12	-2.29	Null	UI-R-BT1-BKC-B-10-0-UI.S1 UI-R-BT1 RATTUS NORVEGICUS CDNA CLONE UI-R-BT1-BKC-B-10-0-UI
**NM_133387.1**	1	-2.12	-2.34	Tmlhe	trimethyllysine hydroxylase, epsilon
**BF283600**	1	-2.14	-2.63	Papss2_Predicted	3'-phosphoadenosine 5'-phosphosulfate synthase 2
**NM_138511.1**	1	-2.16	-1.96	Gpc2	glypican 2
**AW254017**	1	-2.17	-1.90	Col5a1	collagen, type V, alpha 1
**BF393863**	1	-2.19	-2.46	Chd3	chromodomain helicase DNA binding protein 3
**AI233702**	1	-2.19	-2.22	Cmbl	carboxymethylenebutenolidase homolog (Pseudomonas)
**NM_053580.1**	1	-2.20	-1.86	Slc27a1	solute carrier family 27 (fatty acid transporter), member 1
**NM_053535.1**	1	-2.20	-2.20	Enpp1	ectonucleotide pyrophosphatase/phosphodiesterase 1
**U75928**	1	-2.24	-2.72	Sparc	secreted protein, acidic, cysteine-rich (osteonectin)
**BF284364**	1	-2.25	-2.53	Null	EST448955 RAT GENE

Table S6: Cluster D

Accession	Young	Aged Control	Aged Treated	Symbol	Description
**AI072251**	1	-2.30	-2.02	Null	UI-R-C2-MU-H-04-0-UI.S1 UI-R-C2 RATTUS NORVEGICUS CDNA CLONE UI-R-C2-MU-H-04-0-UI
**AI102097**	1	-2.31	-2.30	Null	EST211386 NORMALIZED RAT BRAIN BENTO SOARES RATTUS SP. CDNA CLONE RBRBY84
**BE112913**	1	-2.32	-2.36	Null	UI-R-BJ1-AWA-C-09-0-UI.S1 UI-R-BJ1 RATTUS NORVEGICUS CDNA CLONE UI-R-BJ1-AWA-C-09-0-UI
**NM_057192.1**	1	-2.33	-2.22	Wipf1	WAS/WASL interacting protein family, member 1
**BE113132**	1	-2.34	-2.54	Rapgef5	Rap guanine nucleotide exchange factor (GEF) 5
**NM_031798.1**	1	-2.43	-2.16	Slc12a2	solute carrier family 12 (sodium/potassium/chloride transporters), member 2
**NM_019123.1**	1	-2.50	-2.40	St6galnac3	N-acetylgalactosaminide alpha-2,6-sialyltransferase 3
**AI411352**	1	-2.50	-2.60	Gja1	gap junction protein, alpha 1, 43kDa
**BF548601**	1	-2.60	-2.79	Null	UI-R-A0-AU-C-02-0-UI.R1 RATTUS NORVEGICUS CDNA
**AA858758**	1	-2.65	-2.75	Rgd1305679	chromosome 6 open reading frame 105
**AI411897**	1	-2.89	-2.52	Null	EST240191 NORMALIZED RAT KIDNEY BENTO SOARES RATTUS SP. CDNA CLONE RKIEK02
**BF397951**	1	-2.94	-3.03	Null	UI-R-BS2-BEF-E-09-0-UI.S1 UI-R-BS2 RATTUS NORVEGICUS CDNA CLONE UI-R-BS2-BEF-E-09-0-UI
**NM_031543.1**	1	-3.42	-4.12	Cyp2e1	cytochrome P450, family 2, subfamily E, polypeptide 1
**AI230431**	1	-4.88	-4.93	Null	EST227126 NORMALIZED RAT EMBRYO BENTO SOARES RATTUS SP. CDNA CLONE REMCW43
**BF417400**	1	-11.29	-13.07	Crebl1	cAMP responsive element binding protein-like 1
